# International clinical guideline for the management of classical galactosemia: diagnosis, treatment, and follow-up

**DOI:** 10.1007/s10545-016-9990-5

**Published:** 2016-11-17

**Authors:** Lindsey Welling, Laurie E. Bernstein, Gerard T. Berry, Alberto B. Burlina, François Eyskens, Matthias Gautschi, Stephanie Grünewald, Cynthia S. Gubbels, Ina Knerr, Philippe Labrune, Johanna H. van der Lee, Anita MacDonald, Elaine Murphy, Pat A. Portnoi, Katrin Õunap, Nancy L. Potter, M. Estela Rubio-Gozalbo, Jessica B. Spencer, Inge Timmers, Eileen P. Treacy, Sandra C. Van Calcar, Susan E. Waisbren, Annet M. Bosch, On behalf of the Galactosemia Network (GalNet)

**Affiliations:** 1 Department of Pediatrics, Emma Children’s Hospital, Academic Medical Center, Meibergdreef 9, 1105 AZ Amsterdam, The Netherlands; 2 Section of Clinical Genetics and Metabolism, Inherited Metabolic Disease Nutrition Department, University of Colorado–Denver School of Medicine, The Children’s Hospital Colorado, Aurora, CO USA; 3 Division of Genetics and Genomics, Boston Children’s Hospital, Harvard Medical School, Boston, MA USA; 4 Broad Institute of MIT and Harvard, Cambridge, MA USA; 5 Department of Pediatrics, Metabolic Unit, University Hospital, University of Padova, Padova, Italy; 6 Department of Metabolic Disorders in Children, Antwerp University Hospital UZA, Edegem, Belgium; 7 University Children’s Hospital, Pediatric Endocrinology, Diabetes and Metabolism, and Institute of Clinical Chemistry, Inselspital, University of Bern, Bern, Switzerland; 8 Metabolic Unit, Great Ormond Street Hospital and Institute of Child Health, University College London, London, UK; 9 National Centre for Inherited Metabolic Disorders, Temple St. Children’s University Hospital, Dublin, Ireland; 10 Department of Pediatrics, APHP, Hopital Antoine Béclère, Cedex Clamart, France; 11 Pediatric Clinical Research Office, Emma Children’s Hospital, Academic Medical Center, Amsterdam, The Netherlands; 12 Birmingham Children’s Hospital, Steelhouse Lane, Birmingham, UK; 13 Charles Dent Metabolic Unit, National Hospital for Neurology and Neurosurgery, Queen Square, London, UK; 14 Medical Advisory Panel, Galactosemia Support Group UK, West Midlands, UK; 15 Department of Pediatrics, University of Tartu, Tartu, Estonia; 16 Department of Genetics, Tartu University Hospital, Tartu, Estonia; 17 Department of Speech and Hearing Sciences, Washington State University, Spokane, WA USA; 18 Department of Pediatrics and Laboratory Genetic Metabolic Diseases, Maastricht University Medical Centre, Maastricht, The Netherlands; 19 Department of Gynecology and Obstetrics, School of Medicine, Emory University, Atlanta, Georgia; 20 Department of Cognitive Neuroscience, Maastricht University, Maastricht, The Netherlands; 21 National Centre for Inherited Metabolic Disorders, Temple St. Children’s University Hospital and Mater Misericordiae University Hospital, Dublin, Ireland; 22 Department of Molecular and Medical Genetics, School of Medicine, Oregon Health and Science University, Portland, OR USA; 23 Division of Genetics and Genomics, Boston Children’s Hospital and Harvard Medical School, Boston, MA USA

## Abstract

Classical galactosemia (CG) is an inborn error of galactose metabolism. Evidence-based guidelines for the treatment and follow-up of CG are currently lacking, and treatment and follow-up have been demonstrated to vary worldwide. To provide patients around the world the same state-of-the-art in care, members of The Galactosemia Network (GalNet) developed an evidence-based and internationally applicable guideline for the diagnosis, treatment, and follow-up of CG. The guideline was developed using the Grading of Recommendations Assessment, Development, and Evaluation (GRADE) system. A systematic review of the literature was performed, after key questions were formulated during an initial GalNet meeting. The first author and one of the working group experts conducted data-extraction. All experts were involved in data-extraction. Quality of the body of evidence was evaluated and recommendations were formulated. Whenever possible recommendations were evidence-based, if not they were based on expert opinion. Consensus was reached by multiple conference calls, consensus rounds via e-mail and a final consensus meeting. Recommendations addressing diagnosis, dietary treatment, biochemical monitoring, and follow-up of clinical complications were formulated. For all recommendations but one, full consensus was reached. A 93 % consensus was reached on the recommendation addressing age at start of bone density screening. During the development of this guideline, gaps of knowledge were identified in most fields of interest, foremost in the fields of treatment and follow-up.

**Electronic supplementary material:**

The online version of this article (doi:10.1007/s10545-016-9990-5) contains supplementary material, which is available to authorized users.

## Recommendations

### Diagnosis

**Recommendation #1 (+)**

Clinicians should confirm the diagnosis of CG by the measurement of GALT enzyme activity in red blood cells (absent or significantly decreased), and/or GALT gene analysis. It is enough to confirm the diagnosis by genetic analysis only, if the detected variations are reported as disease causing in genetic variation databases (Calderon et al. 2007; http://www.arup.utah.edu/database/galt/galt_welcome.php) and the biological parents each carry one variation.

**Recommendation #2 (expert opinion, +)**

Clinicians should treat patients with a red blood cell GALT enzyme activity below 10 % and/or pathologic variations on both alleles of the GALT gene, including p.S135L, with a galactose-restricted diet. There is not enough evidence to conclude whether patients with 10–15 % red blood cell residual GALT activity should or should not be treated.

**Recommendation #3 (expert opinion, +)**

We recommend not to treat patients with the Duarte variant.

### Dietary management

**Recommendation #4 (++)**

Clinicians should immediately commence a galactose-restricted diet (e.g., soy-based, casein hydrolysate or elemental formula) if classical galactosemia is suspected in an infant, without waiting for confirmation of the diagnosis.

**Recommendation #5 (expert opinion, +)**

We recommend treating patients with CG with a life-long galactose-restricted diet that only eliminates sources of lactose and galactose from dairy products, but permits galactose from non-milk sources that contribute minimal dietary galactose. Within this definition we accept that small amounts of galactose are present in specific mature cheeses and caseinates. At present there is insufficient evidence to support a specific age-related recommendation for the quantity of galactose allowed in the diet.

**Recommendation #6 (+)**

We recommend allowing any amount and type of fruits, vegetables, legumes, unfermented soy-based products, mature cheeses (with galactose content <25 mg/100 g), and the food additives sodium or calcium caseinate, in the diet for classical galactosemia. Although higher in galactose, all fermented soy-based products can be allowed in the small quantities that are typically used in the diet.

**Recommendation #7 (+)**

We recommend an annual dietary assessment of calcium and vitamin D intake with measurement of plasma total 25-OH-vitamin D levels. Both calcium and vitamin D should be supplemented as necessary following the age-specific recommendations for the general population.

### Biochemical follow-up

**Recommendation #8 (++)**

In the first year of life clinicians should measure red blood cell Gal-1-P levels at diagnosis, and after 3 and 9 months of dietary galactose restriction.

**Recommendation #9 (expert opinion, +)**

We recommend measuring red blood cell Gal-1-P levels yearly after the first year of life until an individual baseline has been established.

**Recommendation #10 (expert opinion, +)**

We recommend measuring red blood cell Gal-1-P levels in case of increase in galactose intake and concern about intoxication.

**Recommendation #11 (expert opinion, +)**

The clinical utility of serial blood or urinary galactitol measurement is limited.

## Long-term complications

### Cognitive development

**Recommendation #12 (++)**

Clinicians should refer patients for testing of developmental quotient (DQ) and intellectual quotient (IQ), to obtain a well-validated measure of development and cognitive abilities. At minimum, testing should be done at:

Age 2–3 years: to assess early speech/language and motor development in time for early intervention, using a standardized test instrument such as the Bayley Scales of Infant and Toddler Development (BSID) or a similar measure.

Age 4–5 years: to assess school readiness and need for occupational therapy and speech-language therapy, using a standardized test instrument such as the Wechsler Preschool and Primary Scale of Intelligence (WPPSI) or a similar measure.

Age 8–10 years: to assess cognitive development, specific areas of strengths and weaknesses and the need for special therapies, using a standardized test instrument such as the Wechsler Intelligence Scale for Children (WISC) or a similar measure.

Age 12–14 years: to assess cognitive development and specific areas of strengths and weaknesses and to assess the need for special therapies, using a standardized test instrument such as the Wechsler Intelligence Scale for Children (WISC) or a similar measure.

Age 15 years and older: according to needs, specific questions.

(consider combining these assessments with speech and language screening, recommendation #15, and psychosocial development screening, recommendation #21)

**Recommendation #13 (expert opinion, +)**

For obtaining a measure of functioning when formalized testing is not possible or when additional assessments are needed between formalized testing points, we recommend using a validated parent/informant questionnaire, such as the Adaptive Behavior Assessment System (ABAS) or a similar measure.

**Recommendation #14 (expert opinion, +)**

We recommend a clinical assessment of executive function, if feasible in the clinic, with specific attention to processing speed and visual spatial comprehension. In children (8–10 years) as a first screening use the Behavior Rating Inventory of Executive Function (BRIEF), and in adolescents (12–14 years) and in young adults (18–20 years) use the Cambridge

Neuropsychological Test Automated Battery (CANTAB), the Amsterdam Neuropsychological Tasks program (ANT) or a similar measure, with follow-up, as needed.

### Speech and language

**Recommendation #15 (++)**

All children with CG should be screened for speech and language delay at ages 7–12 months, 2 years, 3 years, and 5 years (consider combining with screening for cognitive disorders, recommendation #12). If children show low or borderline speech and language development, full assessments should be conducted.

**Recommendation #16 (expert opinion, +)**

We recommend that an assessment of speech and language includes hearing screening, a brief assessment of pre-linguistic communication (<2 years of age) and expressive, receptive, and pragmatic language use, structure-function examination, motor speech (observation of respiration, resonance, voice, articulation), and speech intelligibility for all children not meeting age appropriate milestones. We recommend a cognitive evaluation, as well if, a disorder is suspected.

**Recommendation #17 (expert opinion, +)**

For children who are not meeting age appropriate speech or language milestones, we recommend treatment based on guidelines for treatment of speech, language, and voice disorders in the general population. Therapy should begin during the first year of life and include modeling and training of gestural communication to increase infant and toddler language development. Play-based milieu for language development is recommended during the second year of life. Individual speech therapy focused on high repetition of a small number of targets should begin during the second year of life and continue as needed throughout the preschool and elementary school years. Respiration, phonation, and resonance deficits should also be addressed.

### Neurological complications

**Recommendation #18 (++)**

Clinicians should screen patients with CG for neurological involvement by clinical examination from the age of 2–3 years. Such screening should include examination for ataxia, tremor, dysmetria, and dystonia. If a specific neurological deficit is noted, monitoring of progression with a designated scale is advised. It is suggested to screen adult patients annually and to record progression, if any. Pediatric patients could be screened more frequently (every 6 months) in order to identify potentially modifiable neurological problems.

**Recommendation #19 (+)**

We recommend asking patients or caregivers about onset of seizure and seizure-like activity since previous examination and perform an EEG, if indicated.

**Recommendation #20 (expert opinion, +)**

We do not recommend routine brain and spinal cord imaging in the follow-up of patients with CG.

In those patients with significant or progressive neurological symptoms and signs, imaging may be warranted to (1) determine if a second condition is present or (2) further define the development and progression of neuroradiology findings in individual patients.

### Psychosocial development

**Recommendation #21 (expert opinion, +)**

We recommend screening children for psychosocial deficits, including autism spectrum disorders, sensory integration problems, depression and anxiety, using standardized questionnaires such as the Behavior Assessment System for Children, Second Edition (BASC-2) in English or a similar tool in other language. We recommend performing this screening at age 2 years in combination with screening for speech and language delays (see recommendation #15) and to combine this screening with developmental testing at ages 4–5 years, 8–10 years, and 12–14 years (see recommendation #12).

**Recommendation #22 (+)**

We recommend screening adults for mental health issues with validated questionnaires that include brief scales for Anxiety and Depression, such as the NIH PROMIS Questionnaires, Beck Anxiety Inventory (BAI), Beck Depression Inventory (BDI) or similar measures. With adults, we recommend discussing living situations, work and/or educational situations, satisfaction with social relationships, and sexual intimacy during outpatient clinic visits and to refer for professional consultation, if necessary.

**Statement #23 (expert opinion, ↓)**

We do not recommend routine Health-Related Quality of Life (HRQoL) evaluations.

### Endocrinology/Fertility

**Recommendation #24 (++)**

Girls with CG should be screened for hypergonadotropic hypogonadism if they reach the age of 12 years with insufficient secondary sex characteristics or if they reach the age of 14 years with no regular menses. Screening should include follicle-stimulating hormone and 17-beta-estradiol.

**Recommendation #25 (expert opinion, +)**

We recommend to consider follicle stimulating hormone level, growth, and psychosocial maturity of the individual girl, for determination of age at start of treatment. For puberty inducement, a low dose estrogen in a step-wise escalating dose is used, then later combined with cyclic progesterone for regular withdrawal bleeds. We recommend considering referral to a pediatric endocrinologist.

**Recommendation #26 (expert opinion, +)**

We recommend not using anti-Müllerian hormone and ovarian imaging routinely for follow-up as these have not been shown to accurately predict pubertal development or fertility outcome.

**Recommendation #27 (+)**

We do not recommend endocrine follow-up for Duarte Galactosemia, as there is no evidence that the ovaries are affected.

**Recommendation #28 (expert opinion, +)**

We recommend that girls and women with CG, who have gone through puberty and established regular menstrual periods, should be monitored annually for menstrual abnormalities, secondary Amenorrhea, and symptoms of primary ovarian insufficiency (POI). Changes in menses or POI symptoms should be evaluated with a serum follicle-stimulating hormone level. Anti-Müllerian hormone measurement is not helpful in determining which women will undergo POI, but may be helpful in identifying women at risk for imminent POI when it is undetectable. Imaging by pelvic ultrasound or MRI is not recommended unless otherwise clinically indicated.

**Recommendation #29 (expert opinion, +)**

We recommend that women with hypergonadotropic hypogonadism, or primary ovarian insufficiency should be provided counseling and support about their reproductive options and management of irregular or absent menses. Hormone replacement therapy should be initiated with the onset of secondary amenorrhea to reduce the risk of osteoporosis and other complications of primary ovarian insufficiency.

**Recommendation #30 (++)**

We recommend considering a referral to a reproductive endocrinologist for women who desire pregnancy and have been unable to conceive naturally, or for women who desire additional counseling about fertility treatment options including oocyte donation.

**Recommendation #31 (expert opinion, +)**

We recommend providing counseling about adequate birth control methods for women who do not desire pregnancy. While combined oral or transdermal contraceptives may provide cycle control, bone protection, and attenuate hot flashes, they may fail to provide adequate birth control in women with very elevated follicle-stimulating hormone levels. An intrauterine device may provide the lowest failure rate.

**Recommendation #32 (expert opinion, +)**

Fertility preservation may not be successful. Currently, fertility preservation techniques are not yet readily used in everyday practice. We recommend fertility preservation should only be offered with appropriate institutional research ethics review board approval to girls with classical galactosemia at a young pre-pubertal age.

**Recommendation #33 (+)**

We do not recommend routine endocrinology follow-up in males.

### Bone health

**Recommendation #34 (++)**

Clinicians should assess bone mineral density (BMD) by age appropriate dual-energy X-ray absorptiometry (DXA) scan.

**Recommendation #35 (expert opinion, +)(consensus: 93 %)**

We recommend BMD screening from age 8–10 years. With evidence of reduced bone density (Z-score ≤ −2.0), follow-up according to current pediatric bone health guidelines is advised.

Without evidence of reduced bone density, we recommend performing a repeat dual-energy X-ray absorptiometry scan when puberty is complete. We recommend performing follow-up thereafter every 5 years and treatment instituted according to WHO FRAX recommendations.

**Recommendation #36 (+)**

We recommend comprehensive dietary evaluation, optimization of calcium intake if needed, monitoring and if necessary supplementation of vitamin D, hormonal status evaluation and hormone replacement therapy consideration, as well as a regular exercise and assessment of skeletal problems and clinically significant fractures in all patients with CG. Supplementation of vitamin K might be beneficial when combined with an adequate intake of calcium and vitamin D, but currently there is not enough evidence to recommend the routine use of vitamin K.

**Recommendation #37 (expert opinion, +)**

At present there is not enough evidence to justify routine determination of bone turnover markers in patients with CG.

### Cataract

**Recommendation #38 (++)**

Clinicians should refer all patients to an ophthalmologist for evaluation of cataract at the time of diagnosis.

**Recommendation #39 (+)**

We recommend performing ophthalmological follow-up in patients with a cataract at diagnosis until it has fully resolved.

**Recommendation #40 (+)**

We recommend performing ophthalmological screening in all patients who are non-compliant with diet.

## Closing remarks

The presented guideline is the first international and evidence-based guideline for the diagnosis, treatment, and follow-up of CG, and aimed to be applicable worldwide. This guideline should serve as a guide for clinicians and other experts caring for patients with CG. Though great effort was undertaken to formulate evidence-based recommendations, this was frequently hampered by limited evidence resulting in numerous recommendations based on expert opinion (18/40 recommendations, 45 %). The literature concerning CG available to date mostly consists of studies with an observational study-design. In the current era of evidence-based medicine these studies are labeled as having a low to very low level of evidence. Therefore strength of recommendation is ‘discretionary’ for a majority of recommendations in the guidelines, (32/40 recommendations, 80 %) including the recommendations labeled expert opinion. However, as other study designs (such as RCTs or cohort studies) are usually not feasible or may not provide the best design to study characteristics of rare diseases, the strength of the recommendation was upgraded to ‘strong’ when results were consistent across multiple studies, and experts had confidence in the validity of these results (9/40 recommendations, 23 %).

### Future perspectives

Following this conclusion, it is not unexpected that gaps of knowledge were identified in most discussed fields of interest, foremost in the fields of treatment and follow-up. Topics of major importance for future research include: further assessment of which patients should be treated (cut-off enzyme activity), exploration for possible further relaxation of the diet for patients after childhood, exploration of new biomarkers for biochemical follow-up as well as reproductive function, assessment of executive functions in children and adults, and further exploration of bone turnover markers in relation to BMD.

### Guideline update

Revision of this guideline is important as it only represents evidence in predefined areas up to October 2015. Since research in the field of CG is flourishing, it is expected that new information will be gained in the next decade. This guideline is scheduled to be updated in the next 10 years by representatives of the GalNet.

## Electronic supplementary material

Below is the link to the electronic supplementary material.

ESM 1 (PDF 378 kb)

ESM 2 (PDF 441 kb)

ESM 3 (PDF 688 kb)
