# Supplementary material for: International clinical guideline for the management of classical galactosemia: diagnosis, treatment, and follow-up
Source: J Inherit Metab Dis. 2016 Nov 17;40(2):171–6. doi: 10.1007/s10545-016-9990-5 (PMC5306419; doi:10.1007/s10545-016-9990-5)
Supplement: Supplementary file 1 — (PDF 378 kb) [file 10545_2016_9990_MOESM1_ESM.pdf]

International clinical guideline for the management of classical galactosemia: diagnosis, treatment, and follow-up

Lindsey Welling[1] · Laurie E. Bernstein[2] · Gerard T. Berry[3,4] · Alberto B. Burlina[5] · François Eyskens[6] · Matthias Gautschi[7] · Stephanie Grünewald[8] · Cynthia S. Gubbels[3,4] · Ina Knerr[9] · Philippe Labrune[10] · Johanna H. van der Lee[11] · Anita MacDonald[12] · Elaine Murphy[13] · Pat A. Portnoi[14] · Katrin Õunap[15,16] · Nancy L. Potter[17] · M. Estela Rubio-Gozalbo[18] · Jessica B. Spencer[19] · Inge Timmers[20] · Eileen P. Treacy[21] · Sandra C. Van Calcar[22] · Susan E. Waisbren[23] · Annet M. Bosch[1,24] · On behalf of the Galactosemia Network (GalNet)

Received: 16 February 2016 / Revised: 17 August 2016 / Accepted: 29 September 2016
© The Author(s) 2016. This article is published with open access at Springerlink.com

Abstract Classical galactosemia (CG) is an inborn error of galactose metabolism. Evidence-based guidelines for the treatment and follow-up of CG are currently lacking, and treatment and follow-up have been demonstrated to vary worldwide. To provide patients around the world the same state-of-the-art in care, members of The Galactosemia

Communicated by: Georg Hoffmann

The online version of this article (doi:10.1007/s10545-016-9990-5) contains supplementary material, which is available to authorized users.

✉ Annet M. Bosch
a.m.bosch@amc.uva.nl

On behalf of the Galactosemia Network (GalNet)

1 Department of Pediatrics, Emma Children's Hospital, Academic Medical Center, Meibergdreef 9, 1105 AZ Amsterdam, The Netherlands

2 Section of Clinical Genetics and Metabolism, Inherited Metabolic Disease Nutrition Department, University of Colorado–Denver School of Medicine, The Children's Hospital Colorado, Aurora, CO, USA

3 Division of Genetics and Genomics, Boston Children's Hospital, Harvard Medical School, Boston, MA, USA

4 Broad Institute of MIT and Harvard, Cambridge, MA, USA

5 Department of Pediatrics, Metabolic Unit, University Hospital, University of Padova, Padova, Italy

6 Department of Metabolic Disorders in Children, Antwerp University Hospital UZA, Edegem, Belgium

7 University Children's Hospital, Pediatric Endocrinology, Diabetes and Metabolism, and Institute of Clinical Chemistry, Inselspital, University of Bern, Bern, Switzerland

8 Metabolic Unit, Great Ormond Street Hospital and Institute of Child Health, University College London, London, UK

9 National Centre for Inherited Metabolic Disorders, Temple St. Children's University Hospital, Dublin, Ireland

10 Department of Pediatrics, APHP, Hopital Antoine Béclère, Cedex Clamart, France

11 Pediatric Clinical Research Office, Emma Children's Hospital, Academic Medical Center, Amsterdam, The Netherlands

12 Birmingham Children's Hospital, Steelhouse Lane, Birmingham, UK

13 Charles Dent Metabolic Unit, National Hospital for Neurology and Neurosurgery, Queen Square, London, UK

14 Medical Advisory Panel, Galactosemia Support Group UK, West Midlands, UK

15 Department of Pediatrics, University of Tartu, Tartu, Estonia

16 Department of Genetics, Tartu University Hospital, Tartu, Estonia

17 Department of Speech and Hearing Sciences, Washington State University, Spokane, WA, USA

18 Department of Pediatrics and Laboratory Genetic Metabolic Diseases, Maastricht University Medical Centre, Maastricht, The Netherlands

19 Department of Gynecology and Obstetrics, School of Medicine, Emory University, Atlanta, Georgia

20 Department of Cognitive Neuroscience, Maastricht University, Maastricht, The Netherlands

Network (GalNet) developed an evidence-based and internationally applicable guideline for the diagnosis, treatment, and follow-up of CG. The guideline was developed using the Grading of Recommendations Assessment, Development, and Evaluation (GRADE) system. A systematic review of the literature was performed, after key questions were formulated during an initial GalNet meeting. The first author and one of the working group experts conducted data-extraction. All experts were involved in data-extraction. Quality of the body of evidence was evaluated and recommendations were formulated. Whenever possible recommendations were evidence-based, if not they were based on expert opinion. Consensus was reached by multiple conference calls, consensus rounds via e-mail and a final consensus meeting. Recommendations addressing diagnosis, dietary treatment, biochemical monitoring, and follow-up of clinical complications were formulated. For all recommendations but one, full consensus was reached. A 93 % consensus was reached on the recommendation addressing age at start of bone density screening. During the development of this guideline, gaps of knowledge were identified in most fields of interest, foremost in the fields of treatment and follow-up.

Introduction

Classical galactosemia (CG, MIM 230400) is an autosomal recessive inborn error of galactose metabolism caused by a profound (absent or barely detectable) deficiency of galactose-1-phosphate-uridyltransferase (GALT; EC 2.7.7.12), which leads to the accumulation of the metabolites galactose-1-phosphate (Gal-1-P), galactitol, and galactonate. The human *GALT* gene maps to chromosome 9p13 (Flanagan 2009). The incidence of CG widely varies worldwide, with an estimated incidence of 1:19,000 to 1:44,000 in Europe (with a higher incidence in the Irish Traveller population) and the USA (Bosch 2006; Ounap et al 2010; Waisbren et al 2012; Coss et al 2013). After the ingestion of galactose from breast milk or infant formula, affected neonates develop a life-threatening illness with feeding difficulties, liver failure, *E. coli* sepsis, and bilateral cataract in the first weeks of life. While the acute symptoms resolve rapidly upon initiation of a lactose-free and galactose-restricted diet, such as a soy-based formula, many patients, irrespective of the severity of the illness in the newborn period (Hughes et al 2009), suffer from long-term complications such as cognitive deficits, speech and language deficits, neurological abnormalities, and hypergonadotropic hypogonadism in females (Donnell et al 1961; Komrower and Lee 1970; Kaufman et al 1981; Waisbren et al 1983; Kaufman et al 1994). The phenotypic spectrum of the disease is extremely wide, varying from normal development to severe complications affecting independence. It is debated whether these complications are progressive. The disease mechanism is not fully understood. Endogenous production of galactose is significant, causing a persistent elevation of Gal-1-P and galactitol in patients with CG, even on a galactose restricted diet (Ning et al 2001; Schadewaldt et al 2004; Huidekoper et al 2005). Elevated Gal-1-P levels competitively inhibit several metabolic pathways including those involved in the galactosylation of proteins and lipids (Fridovich-Keil and Walter 2008). Both Gal-1-P and galactitol levels have a high inter- and intra-personal variability and do not seem to predict outcome, limiting their usefulness for biochemical monitoring (Hutchesson et al 1999).

The UK Galactosemia Steering Group established general national recommendations (Walter et al 1999), but did so over a decade ago and without a formal assessment of the evidence. No other guidelines, meeting current standards of evidence-based medicine, have been published to date. Treatment and follow-up of CG vary significantly worldwide (Jumbo-Lucioni et al 2012). To provide patients around the world the same state-of-the-art care, we developed an evidence-based and internationally applicable guideline. This guideline addresses all important topics with regard to diagnosis, treatment, and follow-up of CG, and can be used as a reference. The authors have chosen not to address newborn screening (NBS) in this guideline. Additionally, a summary of all recommendations is provided as a supplement for easy use in clinical setting. The target users of this guideline are medical doctors, dieticians, psychologists, speech and language therapists, and other multidisciplinary team members involved in care for patients with CG. At this time we propose that this guideline may be applied to all patients with a GALT enzyme activity below 15 %. While CG is defined by a profound impairment of GALT enzyme activity (absent or barely detectable) and/or the presence of two null or severe missense variations, through newborn screening patients with low but not profoundly deficient GALT enzyme activities up to 15 % are detected. Future research is necessary for evidence based advise on treatment in these children.

[21] National Centre for Inherited Metabolic Disorders, Temple St. Children's University Hospital and Mater Misericordiae University Hospital, Dublin, Ireland

[22] Department of Molecular and Medical Genetics, School of Medicine, Oregon Health and Science University, Portland, OR, USA

[23] Division of Genetics and Genomics, Boston Children's Hospital and Harvard Medical School, Boston, MA, USA

[24] Department of Pediatrics, Emma Children's Hospital, Academic Medical Center, room H7-270, ZIP code: 22660, 1100 DD Amsterdam, The Netherlands

Methodology

The Grading of Recommendations Assessment, Development, and Evaluation (GRADE) system was used to methodologically design and develop this guideline (The GRADE Working Group 2009).

Guideline participants and key questions

The development of this guideline was initiated by the Galactosemia Network (GalNet). Important topics and problems in the field of diagnosis, treatment, and follow-up of CG were explored. Ten different fields of interest were identified: 1) diagnostics, 2) biochemical follow-up, 3) dietary management, 4) cognitive development, 5) speech and language development, 6) neurological complications, 7) psychosocial development and mental health, 8) endocrinology and fertility, 9) bone health, and 10) ophthalmological complications. At the start of the GalNet in 2012, all Society for the Study of Inborn Errors of Metabolism (SSIEM) members were invited to participate in the network. All who expressed their interest in the GalNet were invited to a first meeting in January 2014 in Maastricht (the Netherlands), where key questions in each field of interest were formulated by the experts of the GalNet, in collaboration with representatives of the European Galactosemia Society (patient organization) (Table 1). Experts attending this meeting were invited to participate in guideline development, and a 21-member guideline expert panel was formed. Based on their specialty, experts from this panel participated in working groups focusing on key questions related to their field of interest.

Information sources and search strategy

The first author and an experienced clinical librarian conducted formalized literature searches, using a different search strategy for each set of key questions belonging to a specific field of interest (for example: 'Bone health'). Databases searched included MEDLINE, EMBASE, PsychInfo, Web of Science, and Cochrane library, as applicable per set of key questions. No filters were used for the searches. Search strategies are provided in Supplement 1.

Eligibility criteria of studies

Study design Studies with the following design were included: randomized controlled trials (RCT), non-RCT, cohort studies, case–control studies, case series, cross-sectional studies, and experimental studies. Case reports and conference abstracts were excluded. Studies in humans and in vitro studies with human tissue were included, animal studies were excluded.

Characteristics Studies published in any year and written in English were included. Studies reported in any other language were excluded. Full-text version of the articles had to be available.

Study selection

Titles of the identified articles were screened (by first and last author) and immediately discarded when clearly not on the topic or not meeting the inclusion criteria. Abstracts of the remaining articles were read (by first and last author) and relevant articles meeting the inclusion criteria were included. When necessary the entire article was read (by first author) before deciding to include or exclude the article.

Data-extraction

The first author and one of the working group experts conducted data-extraction (identification of key data elements) per manuscript. All experts were involved in data-extraction for one or multiple key questions. Evidence was summarized per recommendation (see Summary of evidence Tables, provided in Supplement 2). Based on this summary, each recommendation was categorized as "supported by evidence" or as "expert opinion". If the recommendation was categorized as 'expert opinion', this was mentioned after the statement.

Critical appraisal and risk of bias assessment

Risk of bias was assessed with the appropriate checklist from SIGN when available. To our knowledge no standardized critical appraisal checklists exist to date for articles with a descriptive study design (case series, cross-sectional studies, experimental studies). Therefore we did not formally assess risk of bias, but did acknowledge the low level of evidence available in these observational, descriptive studies. We recognized in advance that almost all evidence in the field of galactosemia is from descriptive studies. This assumption was confirmed. Thus, the body of evidence in our guideline was uniformly rated as 'low to very low' in terms of the GRADE system. Individual studies were not assigned a level of evidence. Major issues as noted by the investigators were reported in the 'Remarks' section of the Summary of evidence Table and were taken into account when making recommendations.

Strength of recommendation

The body of evidence for each recommendation was 'low to very low'. Accordingly all recommendations (also the recommendations labeled 'expert opinion') were assigned a 'discretionary' strength of recommendation. Only if highly consistent results were found across multiple studies, and if experts had confidence in these results, was the strength of recommendation

Table 1 Key questions

Field of interest	Key questions
Diagnostics (recommendations 1 to 3)	• What is the gold standard for the diagnosis of Classical Galactosemia? (enzyme activity and *GALT* gene mutation analysis, is enzyme alone enough, is mutation alone enough?)<br>• Who needs to be treated? (cut-off enzyme activity?)
Diet (recommendations 4 to 7)	• What is the safe amount of dietary galactose (for the different age groups)?<br>• Based on the answer to above question: should fruit/vegetables/mature cheese/offal/legumes be restricted in the diet?<br>• Should the diet be evaluated regularly for deficiencies? Which deficiencies and how frequently?
Biochemical follow-up (recommendations 8 to 11)	• What parameters need to be followed until stabilization in the first year of life and how frequently?<br>• What (if any) parameters need to be followed up after age 1 year? What is the value of the parameters? At what ages, with what frequency?
Developmental follow-up (recommendations 12 to 14)	• Should IQ be tested? If so, how? At what ages?<br>• Should executive functions be tested? If so how? At what ages?
Speech and language (recommendations 15 to 17)	• Should speech and language be evaluated? If so, how? At what ages?<br>• What treatment should be advised in case of speech and language disorders?
Neurology (recommendations 18 to 20)	• Should patients be screened for neurological pathology? (ataxia, tremor) How? What age and frequency?<br>• Should MRI scan be included in the follow-up of patients?
Psychosocial development/mental health (recommendations 21 to 23)	• Should patients be screened for psychosocial deficits? How? What ages?<br>• Should patients be screened for mental health issues? How? What ages?<br>• Should Quality of Life (QoL) be regularly evaluated?
Endocrinology/fertility follow-up (recommendations 24 to 33)	• How should girls be screened for endocrine dysfunction, and at what ages? (What markers? Is there a role for ultrasound/MRI?)<br>• When should hormonal supplementation be started? Which supplementation is best? Up to what age?<br>• What should be the endocrine follow-up in females at adult age?<br>• Is there a need for endocrine follow-up in males?<br>• Counselling fertility: what do we say?<br>• Fertility preservation: what do we recommend?
Bone (recommendations 34 to 37)	• Should bone health be assessed? How? From what age? How frequently?<br>• What is the clinical relevance of a decrease of -2SD in bone mass? (later in the process this key question was omitted, because this is a general question not concerning CG)<br>• What is advised treatment for bone mass below -1 SD, bone mass -2SD?<br>• Which bone parameters are relevant for follow-up and treatment assessment?
Ophthalmological complications (recommendations 38 to 40)	• In the newborn period which patients need ophthalmological examination?<br>• Which patients need ophthalmological follow-up? At what age, with what frequency?

upgraded to ‘strong’. The strength of recommendation is mentioned after the recommendation: Strong recommendation: ++; discretionary recommendation: +. The body of evidence supporting a recommendation is presented in the Summary of evidence Tables (Supplement 2). Evidence is summarized per key question or set of key questions, and not per recommendation, due to overlap in evidence for multiple key questions. Also, in some cases, multiple recommendations were formulated based on one key question.

Consensus procedures

Experts in speech and language, gynecology, psychology, and nutrition participated in separate working groups that developed recommendations and achieved consensus on topics related to their discipline. An 11-person clinical consensus committee comprised of physicians overseeing care of patients with CG (AB, AMB, FE, IK, EPT, EM, GTB, KO, MERG, MG, PL) not only participated in one or more working groups, but also participated in the consensus process of the recommendations of all the other topics. After the recommendations from each working group were completed on specific topics, and the working group members all agreed with the recommendations, the clinical consensus committee reviewed them to identify potential disagreements. The first and last author made minor revisions and incorporated major revisions for review by the specific working group as well as the clinical

consensus committee. A third review took place at a final in-person consensus meeting, to which all members of the clinical consensus committee were invited. During this final meeting a mediator guided the sessions. Recommendations that were adapted were sent for approval to all experts of the relevant working groups and to Clinical Consensus Committee members not present during the meeting. All authors endorsed the final manuscript prior to its submission.

External review

This guideline was externally reviewed by two independent experts; a pediatric neurologist and internal medicine specialist for endocrinology and metabolic disorders, both with experience in CG and rare disorders. In addition, representatives of the European Galactosemia Patient Society reviewed the guideline. The goal of this review by independent experts was to improve the quality of the guideline and to assess applicability and feasibility. This external review was undertaken with open-ended questions. The main findings of the reviewers were 1) Lengthy but easy to read manuscript 2) Clear, concise, and feasible recommendations 3) Suggestions to improve quality and readability of the text. The suggestions of the reviewers were taken into account by incorporating major revisions to several paragraphs, to shorten the text and improve the quality.

Implementation of this guideline

This guideline is aimed for worldwide adoption and implementation. During the development of the guideline, it was recognized that not all centers would have state-of-the-art facilities or test instruments. Thus, alternatives are provided. All participating experts, the GalNet (www.galactosemianetwork.org/) as well as the European Galactosemia Society (www.galactosaemia.eu/) and the USA Galactosemia Foundation (www.galactosemia.org/), have agreed to be involved in the implementation of this guideline. A short version of all recommendations, easy to utilize in the clinic, is provided as a supplement.

Results

Study selection process

The results of the different search strategies and the results of the study selection processes are presented in Supplement 1.

Risk of bias assessment

Only one study was identified for which an appropriate critical appraisal and risk of bias assessment checklist from SIGN was available. This study, a RCT, was scored to be of high quality with low risk of bias (Panis et al 2006b). Two studies with a descriptive study design were excluded as evidence (Pesce and Bodourian 1982, Milánkovics et al 2010), as determined by the authors, for reasons reported in the Summary of evidence Tables (Supplement 2).

Consensus procedures

E-mail rounds

A total of 40 recommendations were formulated. After the clinical consensus committee reviewed the recommendations via one or two e-mail rounds, a 100 % consensus was reached for all recommendations with regard to dietary management, diagnostics, neurology, and speech and language. Less than a full consensus was reached for one recommendation in each of the fields of bone health, developmental follow-up and endocrinology/fertility follow-up, and for two recommendations in each of the fields of biochemical follow-up, cataract, and psychosocial development/mental health.

Final consensus meeting

The final consensus meeting took place in October 2015 in Amsterdam, the Netherlands. Nine of 11 clinical consensus committee members attended the meeting. All nine recommendations for which no consensus was reached during the e-mail rounds were discussed, adapted, and adopted during the meeting. All members attending the meeting agreed to eight of these nine recommendations, and the two members not attending the meeting provided consensus for adoption of all nine recommendations later via e-mail. Experts that were not part of clinical consensus committee, but who were involved in formulating these nine recommendations, were also contacted afterward via e-mail and all gave consensus for the recommendations that were adapted during the final consensus meeting (and adopted by the clinical consensus committee members attending this meeting). One member did not provide consensus for one recommendation (#35) with regard to bone health. Therefore a 93 % consensus was reached for this particular recommendation.

Recommendations

Diagnosis

CG is defined by a profound impairment of GALT enzyme activity (absent or barely detectable) and/or the presence of two null or severe missense variations. Untreated patients demonstrate a multi-organ toxicity in the newborn period that is lactose intake- and duration-dependent. Through newborn

screening (NBS), patients with low but not profoundly deficient GALT enzyme activities up to 15 % are detected who do not demonstrate the p.S135L variation (c.404C>T (p.Ser135Leu)) or Duarte genotypes. Future research is necessary for evidence-based advise on treatment in these children. At this time we propose that this guideline may be applied to all patients with a GALT enzyme activity below 15 %.

In some countries, CG patients are identified through NBS, but other countries chose not to include CG in their NBS program due to ongoing uncertainties about the balance between risks and benefits (Jumbo-Lucioni et al 2012). NBS prevents development of critical illness and death, but it probably does not change frequency of long-term complications. Varela-Lema et al recently concluded that existing evidence remains insufficient to establish the appropriateness of NBS for CG (Varela-Lema et al 2016). NBS is not further addressed in this guideline.

The most commonly used methods to diagnose CG, after clinical suspicion or identification through NBS, are measurement of GALT enzyme activity in red blood cells (RBC), and (confirmation by) *GALT* genetic analysis. Usually GALT activity is expressed as the percentage of the activity of healthy non-carrier controls. In the database of Calderon et al, last updated in January 2013 (www.arup.utah.edu/database/GALT/GALT_welcome.php), 336 different variations had been reported (Calderon et al 2007). Only one study reports on the diagnostic process in CG with a combination of GALT enzyme activity measurement and genetic analysis of the most common variations in the *GALT* gene (Calderon et al 2007). Detection of genetic variations accorded with enzyme activity measurement in 93 % of samples, increasing to 99 % after samples with discordant results were fully sequenced. For measurement of GALT enzyme activity most laboratories use radioactive assays, which are laborious and/or are incapable of measuring low enzyme activity (Li et al 2011). Other methods have been developed, including assays using ultra performance liquid chromatography-tandem mass spectrometry, liquid chromatography-tandem mass spectrometry, and reversed-phase high performance liquid chromatography with UV detection (Xu et al 1995; Ko et al 2010; Li et al 2010; Lindhout et al 2010; Li et al 2011). Measurement of GALT activity in RBC is unreliable after blood transfusion, and genetic analysis or enzyme activity measurement in lymphocytes should be performed. Supportive diagnostic methods (before the final diagnosis is made) include measurements of total blood galactose, RBC Gal-1-P, and/or urinary galactitol.

Recommendation #1 (+)

Clinicians should confirm the diagnosis of CG by the measurement of GALT enzyme activity in red blood cells (absent or significantly decreased), and/or *GALT* gene analysis. It is enough to confirm the diagnosis by genetic analysis only, if the detected variations are reported as disease causing in genetic variation databases (Calderon et al 2007; http://www.arup.utah.edu/database/galt/galt_welcome.php) and the biological parents each carry one variation.

Treatment

There is worldwide consensus that patients with the classical form of galactosemia should be treated with a galactose-restricted diet (Jumbo-Lucioni et al 2012).

p.S135L variant

A well-known variant with a GALT activity <15 %, is the p.S135L variation. Worldwide patients homozygous for this variation, which is most often seen in people of African descent, are treated with a galactose-restricted diet. Homozygous patients have RBC GALT activities with values between 0.2 and 1.7 % of normal activity, with enzyme activities of up to 10 % in other tissues such as liver and intestinal mucosa, and may have better long-term clinical outcomes than patients with CG (Elsas et al 1994; Wang et al 1998). Genotype was not confirmed in the patients in the cited studies.

One study showed a lower galactitol excretion in four patients with p.S135L/p.S135L than in patients homozygous for the p.Q188R (c.563A>G (p.Gln188Arg)) variation and p.Q188R/other patients, but the levels were still above the reference range (Palmieri et al 1999). In vivo galactose oxidation capacity in patients with p.S135L/p.S135L is comparable to healthy controls (Berry et al 1995; Lai et al 1996; Berry et al 1997; Berry et al 2000). In vitro galactose oxidation capacity in lymphoblastic cells lines of two patients homozygous for p.S135L was significantly higher than in patients homozygous for p.Q188R, but reduced compared to control cells (Yager et al 2001), and after incubation with 1-^{13}C galactose Gal-1-P levels in p.Q188R/p.Q188R and p.S135L/p.S135L lymphoblastic cells were fully comparable to control cells (Wehrli et al 2002). There is no difference in the UDPgal and UDPglu levels between p.S135/p.S135L and p.Q188R/p.Q188R cells (Wehrli et al 2002). IgG N-glycans from one pediatric patient with p.S135L/p.S135L (on a galactose intake of 300 mg/day) showed decreased galactosylation in comparison with healthy children, similar to p.Q188R/p.Q188R patients (a galactose intake of <50 mg/day) (Coss et al 2014). This is indicative of ongoing N-glycan processing defects in these patients.

Duarte galactosemia

Patients with Duarte variant galactosemia (DG) have one GALT allele that is severely impaired, and a second GALT allele (Duarte-2, D2) that is partially impaired. At least five sequence changes on D2 alleles have been demonstrated so far: a p.N314D (c.940A>G (p.Asn314Asp) missense substitution, three intronic base changes, and a 4 bp deletion in the 50

proximal sequence (Kozak et al 1999; Carney et al 2009). DG is associated with a mean residual enzyme activity of 14–25 % (Fridovich-Keil et al 2014), in contrast to most patients homozygous for the classical p.Q188R variation, who usually have a severely deficient (<1 %) residual GALT enzyme activity (Wang et al 1998). Individuals with DG have a galactose oxidation capacity comparable to healthy controls (Berry et al 1995; Lai et al 1996; Berry et al 1997; Berry et al 2000). Children with the DG variant are not known to present with clinical symptoms, but are detected by newborn screening, and since the start of these programs there is debate in the USA about whether or not these children need treatment and/or follow-up. Long-term clinical outcome in untreated affected individuals is assumed to be normal, but data are scarce with a limited number of studies with regard to clinical outcome and biochemical follow-up of DG. To our knowledge, currently the most common practice in Europe is not to treat and follow-up individuals with DG, while in the USA some metabolic centers prescribe a galactose-restricted diet in the first year of life (Ficicioglu et al 2008; Fridovich-Keil et al 2014).

Three papers reported on neonatal symptoms in DG variants (genotype confirmed). One paper reported mild unspecified symptoms in DG variants, however, this manuscript was excluded as evidence as the five reported DG variant children suffered from multiple pathologies such as cardiac disease and dysmorphic features (Milánkovics et al 2010). Two other papers reported no symptoms and no abnormalities of liver function (Badawi et al 1996; Ficicioglu et al 2008). Reports of long-term outcomes in DG indicate normal IQ scores, language skills, FSH values, and ophthalmologic examinations in untreated children aged 1–6 years with DG, as well as in those treated with a galactose-restricted diet in the first year of life (Ficicioglu et al 2008; Ficicioglu et al 2010). Levels of FSH in female children with DG (up to 10.5 years) are comparable to healthy controls (Badik et al 2011). One study reported a higher percentage of children with DG enrolled in special education services, primarily speech and language, compared to the general population, but these results were not significant (Powell et al 2009), and detailed information about the nature of the special educational services was not available for all the children with DG. A pilot study assessed developmental outcome in ten children with DG compared to five unaffected siblings from the same group of families (all children aged 6–11 years) (Lynch et al 2015). In this small sample, some differences in socio-emotional development, in delayed recall, and in auditory processing speed between children with DG and the unaffected siblings were found.

During the first year of life, children with DG who are untreated have significantly higher levels of RBC Gal-1-P, galactitol, and galactonate when compared to those children with DG started on a diet after diagnosis (who have levels of Gal-1-P and galactitol within the reference range at the age of 4 weeks) and also have higher levels than patients with CG on a galactose-restricted diet (Ficicioglu et al 2005; Ficicioglu et al 2008). In children with DG, who are untreated, levels of Gal-1-P and galactitol gradually decrease to a level within the reference range at the age of 1 year without intervention (Ficicioglu et al 2008). After children with DG have RBC Gal-1-P values within the reference range, they still demonstrate increased levels of other galactose metabolites, including RBC galactitol (<10 years) and RBC galactonate (1–6 years), that correlate with galactose intake (Schwarz et al 1985; Ficicioglu et al 2010).

Recommendation #2 (expert opinion, +)

Clinicians should treat patients with a red blood cell GALT enzyme activity below 10 % and/or pathologic variations on both alleles of the *GALT* gene, including p.S135L, with a galactose-restricted diet. There is not enough evidence to conclude whether patients with 10–15 % red blood cell residual GALT activity should or should not be treated.

Recommendation #3 (expert opinion, +)

We recommend not to treat patients with the Duarte variant.

Dietary management

Ingestion of galactose derived from lactose in breast milk or whey-based formula causes life-threatening symptoms in the first weeks of life in patients with a severe deficiency of the GALT enzyme activity. These symptoms quickly resolve upon initiation of a galactose-restricted diet. While in some countries CG is part of the newborn screening panel, many patients will have presented with symptoms before referral for abnormal newborn screening. For most infants, the galactose-restricted diet includes discontinuation of breast milk or whey-based infant formulas and initiation of a soy-based formula, but an elemental formula may also be chosen (Jumbo-Lucioni et al 2012). There is an ongoing debate about the safety of soy-based formulas, due to the mild estrogenicity of soy. However, a recent review and meta-analysis demonstrated no effects on long-term growth, bone health and metabolic, reproductive, endocrine, immune and neurological functions, and neurocognitive parameters in non-galactosemic children treated with soy-based formulas (Vandenplas et al 2014). Elemental formulas containing L-amino acids are more expensive than soy-based formulas and, at this time, there is no evidence that consuming an elemental formula provides a clinical benefit for infants with CG. Casein hydrolysate formulas, containing medium-chain fatty acids, may be beneficial for infants with significant liver disease. Casein protein hydrolysate formula (derived from cow's milk) does contain traces of residual lactose (<10 mg/100 mL), but this is considered safe in CG. In contrast, whey-based hydrolysates contain more residual lactose and

Table 2 Examples of galactose content of food products, adapted from (Portnoi and MacDonald 2009; Van Calcar et al 2014; Portnoi and MacDonald 2015)

	United States (Van Calcar et al 2014) Galactose content (mg/100 g food)[a] Mean ± SD (range[b])	United Kingdom (Portnoi and MacDonald 2009, 2015) Lactose[e] content (mg/100 g food) Mean (range) (l.o.d. = limit of detection)
Dairy based products		
Cheddar cheese aged traditional	9.5 ± 17.9 (<2.8 to 104.3)	
UK west country Cheddar aged traditional		3.6 (<2.8–11.4)
Gruyere	4.1 ± 1.2 (<2.8 to 5.1)	
UK Gruyere		Not detectable (<3.5 l.o.d.)
Emmentaler/Swiss	3.5 ± 1.2 (<2.8 to 7.4)	
UK Emmentaler		Not detectable (<3.5 l.o.d.)
Jarlsberg	<2.8 (all <2.8)	
UK Jarlsberg		Not detectable (<10 l.o.d.)
American Parmesan, brick (aged >10 month) momo)months)	18.3 ± 33.3 (<2.8 to 156)	
American Parmesan, grated	9.7 ± 12.0 (<2.8 to 23.6)	
UK Italian Parmesan (usually 2 year old) block/grated		Not detectable
Sodium or calcium caseinate	35.5 ± 37.7 (<5.1 to 95.5)	
UK Comte cheese		Not detectable (0.43 (0.05–1.86)))
Butter oil/milkfat		3 samples mean 0.9 mg/100 g Need median 4.32 and range <0.05–1.86
UK butter oil/milk fat		<0.05 to 2.3
UK ghee		<0.05 to 2.9
UK butter		685 to 688
Plant-based products[c]		
Various fruits (raw or processed)	9.7 ± 7.9 (1.0 to 44.5)	
Various vegetables (raw or processed)	9.3 ± 11.4 (ND to 77.2)	
Fruit and vegetable juices	18.3 ± 14.0 (4.0 to 46.4)	
Legumes		
Garbanzo beans (cooked or processed)	149.5 ± 197.(24.6 to 443.8) t443.8)443.8443.8)	
Other legumes (cooked or processed)[d]	46.2 ± 63.1 (ND to 174.8)	
Soy products		
Soy beans, whole	43.8	
Soy milk (made from whole soy beans)	5.1 ± 0.4 (4.8 and 5.3)	
Tofu, silken	90 (dry wt)	
Fermented soy products		
Miso paste	290.7 ± 121.2 (139 to 433)	
Soy sauce[a]	361.7 ± 147.3 (240 to 590)	
Sufu (fermented tofu)	912 (dry wt)	

[a] All values are reported as mg galactose in 100 g of product except for soy sauce values which were reported as mg galactose in 100 mL. All reported values are based on 100 g wet weight; values for dried weight were not considered in the determination of means and ranges. The only exceptions to this are for tofu and sufu since wet weights were not given in the references

[b] The lower detection limit varied depending on the methodology utilized in each paper and is reported with a "b" sign or ND (not detected). For any sample containing lactose, 53 % of total lactose was considered galactose, based on molecular weight of 342 for lactose and 180 for galactose

[c] For all plant products, only the reported free galactose content was considered in the determination of mean ± SD. Any galactose in a bound form was not considered to contribute to the galactose content of any food

[d] Other legumes include one or more analyses for kidney beans, pinto beans, black beans, white beans, lentils, and pink-mottled cream beans

[e] The amount (in mg) of galactose is approximately half the amount of lactose

are not advocated for infants with CG. Due to the high galactose content of all animal milks and other dairy products (cow's milk contains 2400 mg galactose/100 mL) all clinics eliminate these products from the diet (Jumbo-Lucioni et al 2012; Adam et al 2015), but extent of galactose and lactose restriction varies between countries and even from clinic-to-clinic within the same country. Also there is a variation in the extent of restriction of less obvious sources of galactose (e.g.,

fruits and vegetables that contain free galactose or foods containing trace amounts of lactose).

Recently, Van Calcar et al reviewed the available literature on the galactose content of fruits, vegetables, legumes, dairy products, aged cheeses, and caseinates (Van Calcar et al 2014). In this section of the guideline we refer to Table 2 summarizing the reported galactose contents of food products, as reviewed by Van Calcar et al 2014 and Portnoi and MacDonald 2009. The free galactose content of most fresh or processed fruits, vegetables, and legumes is less than 50 mg/100 g serving (Van Calcar et al 2014), and an adult diet enriched in fruits and vegetables was found to contain only 54 mg of galactose per day (Berry et al 1993). This galactose intake is negligible compared to the endogenous galactose production in humans, which is thought to contribute to development of long-term complications. The endogenous production is strongly age-dependent, with the highest production in newborns (>24.8 mg/kg/day) decreasing to a minimum of 8.4 mg/kg/day in adults (Berry et al 1995; Berry et al 1997; Ning et al 2000; Schadewaldt et al 2004; Berry et al 2004; Huidekoper et al 2005; Schadewaldt et al 2014). Thus, for a 70 kg adult, endogenous galactose production would be more than 580 mg/day. In addition, endogenous production of galactose does not appear to be affected by exogenous intake of galactose (Huidekoper et al 2005). The disparity between dietary intake and endogenous production has prompted many countries to recommend a galactose-restricted diet without restrictions of fruit, vegetables, and legumes. There is no evidence to suggest that consumption of these minor sources of galactose has any adverse effects on long-term clinical status (Bosch et al 2004a; Krabbi et al 2011; Van Calcar et al 2014). Importantly, Portnoi and MacDonald 2009 and Van Calcar et al 2014 demonstrated that the galactose content is low or even negligible in various aged cheeses including Gruyere, mature Parmesan, and Emmentaler cheese (alternative spellings are Emmenthaler, Emmental, Emmenthal) produced in both Europe and North America, although the galactose content in the same type of cheese can vary due to variation in maturation and other biological and processing factors. Cheese is an excellent source of calcium, and many clinics allow and encourage including aged cheese in the diet of patients with galactosemia (Portnoi and MacDonald 2009). However, low-lactose milk aimed at the lactose-intolerant population is contra-indicated in patients with CG. In these products lactose has been hydrolyzed to glucose and galactose by addition of lactase, but still contains considerable galactose content. There is a continuing debate as to whether galactose restriction could be relaxed further with increasing age, especially since there is concern that an overly strict galactose restriction might be harmful (Coss et al 2012). In adolescents and adults with CG, intake of oral galactose of up to 200 mg over 3 weeks, 600 mg over 6 weeks, and 4000 mg galactose over 14 weeks had no effect on RBC Gal-1-P concentrations and these subjects did not develop any clinical manifestations over this short time frame (Berry et al 1993; Bosch et al 2004a; Coss et al 2012). Coss et al further demonstrated that patients with more severe complications have more abnormal IgG glycan patterns and, with exposure up to 2000 mg galactose/day for 16 weeks, the abnormal glycosylation of serum IgG improved in some of the subjects; however, the improvement in glycosylation was highly individual, especially at higher galactose intakes (Coss et al 2012). There are also two published case reports of adults, both homozygous for the p.Q188R variation, who have been off-diet since 3 years of age. These patients ingested approximately 2500 and 9000 mg of galactose per day, yet their clinical outcome and biochemical parameters were comparable to those seen in treated adult patients (Lee et al 2003; Panis et al 2006a). However, experience is limited and there is little evidence to support the safety of discontinuation of the galactose-restricted diet (Table 3).

Recommendation #4 (++)

Clinicians should immediately commence a galactose-restricted diet (e.g., soy-based, casein hydrolysate or elemental formula) if classical galactosemia is suspected in an infant, without waiting for confirmation of the diagnosis.

Recommendation #5 (expert opinion, +)

We recommend treating patients with classical galactosemia with a life-long galactose-restricted diet that only eliminates sources of lactose and galactose from dairy products, but permits galactose from non-milk sources that contribute minimal dietary galactose. Within this definition we accept that small amounts of galactose are present in specific mature cheeses and caseinates. At present there is insufficient evidence to support a specific age-related recommendation for the quantity of galactose allowed in the diet.

Recommendation #6 (+)

We recommend allowing any amount and type of fruits, vegetables, legumes, unfermented soy-based products, mature cheeses (with galactose content <25 mg/100 g), and the food additives sodium or calcium caseinate, in the diet for classical galactosemia. Although higher in galactose, all fermented soy-based products can be allowed in the small quantities that are typically used in the diet.

The opinion about whether to restrict offal in the diet is divided; its galactose content is unknown, but there is no direct evidence of harm. It is a theoretical risk only, therefore it has been decided to put offal in the 'in moderation' section in the 'Current diet restriction for classical galactosemia' table.

Deficiencies

With elimination of dairy products from the diet, patients with CG are at risk for calcium and vitamin D deficiency. The majority of studies report serum calcium levels in the reference range in children and adolescent patients with CG (Rubio-Gozalbo et al 2002; Panis et al 2004; Gajewska et al 2006; Gajewska et al 2008). Only one study with five patients with CG reports significantly lowered concentrations of calcium compared to healthy controls, but the age of these patients was not reported (El-Bassyouni et al 2006). Vitamin D is required for optimal calcium utilization, and normal concentrations of both 1,25-OH vitamin D and 25-OH-vitamin D have been measured in children and adolescents with CG (Rubio-Gozalbo et al 2002; Panis et al 2004; Gajewska et al 2006; Gajewska et al 2008). However, in 80 % of adults with CG, 25-OH vitamin D levels were reported to be below the reference range (Waisbren et al 2012). While some studies report an adequate daily intake of calcium and vitamin D in children and adolescents (Rubio-Gozalbo et al 2002; Panis et al 2004), others report deficient intakes of calcium (Wiesmann et al 1995; Rutherford et al 2002). One study reported that 75 % of adult patients have an intake of vitamin D below the daily recommended intake (Waisbren et al 2012).

Table 3 Current diet restriction for classical galactosemia (adapted from Bernstein et al, Children's Hospital Colorado in collaboration with the Galactosemia Foundation Task Force) (Bernstein et al 2014)

**Allowed foods and ingredients***
Soy-based infant formulas containing soy protein isolate, amino acid-based elemental infant formulas
All fruits, vegetables and their juices, pickled fruit and vegetables
All legumes (e.g., navy beans, kidney beans, garbanzo beans/chick peas, soybeans)
Soy-based products that are not fermented (soy milk, tofu, textured soy protein, hydrolyzed vegetable protein, soy protein concentrate, meat analogs)
Aged cheeses[1]: Jarlsberg, Emmentaler, Swiss, Gruyere, Tilsiter, mature Parmesan, mature Cheddar cheese
Sodium and calcium caseinate
All cacao products except milk chocolate
Eggs
Additional ingredients: natural and artificial flavorings, all gums, including carrageenan
**Foods used in moderation** *
Soy sauce, soy products that are fermented (e.g., miso, natto, tempeh, sufu)
Meat by-products
Offal
**Restricted foods and ingredients***
Breast milk, all milk-based infant formulas
Processed meats using lactose
All milk-based foods and beverages, including low lactose milk, except for caseinates and aged cheeses, listed above
All milk-based ingredients including buttermilk solids, casein, dry milk protein, dry milk solids, hydrolyzed whey protein, hydrolyzed casein protein, lactose, lactalbumin, whey
All cheese and cheese-based products except those listed above
Butter

[1] Galactose content and consequently allowed types of cheese may vary in different countries

* All manufactured foods need to be checked for the presence of milk by reading food ingredient labels

Recommendation #7 (+)

We recommend an annual dietary assessment of calcium and vitamin D intake with measurement of plasma total 25-OH-vitamin D levels. Both calcium and vitamin D should be supplemented as necessary following the age-specific recommendations for the general population.

Biochemical follow-up

Biochemical monitoring in CG is aimed at the follow-up of abnormal parameters of galactose metabolism and evaluation of adherence to the galactose-restricted diet. Currently, monitoring varies widely between centers, and markers measured most frequently include blood galactose, RBC Gal-1-P, and/or urinary galactitol levels (Jumbo-Lucioni et al 2012). Levels of RBC Gal-1-P and urinary galactitol are raised at birth, decrease rapidly after initiation of a galactose-restricted diet, and then stabilize, but remain elevated compared to healthy controls (Waggoner et al 1990; Schweitzer et al 1993; Hutchesson et al 1999; Palmieri et al 1999; Ning et al 2000; Yager et al 2003; Schadewaldt et al 2004; Krabbi et al 2011). There are serious doubts regarding the usefulness of these markers in monitoring the disease and adherence to the diet. There is no clear association/correlation between galactose metabolites and other markers and the development of both acute and long-term complications in patients with CG (Waggoner et al 1990; Schweitzer et al 1993; Cleary et al 1995; Hughes et al 2009). There are no prospective longitudinal studies assessing the predictive value of these markers for the development of long-term complications. Also, several studies have demonstrated no clear increase in RBC Gal-1-P and urinary galactitol levels after short-term oral galactose loading (up to 4000 mg) was given to patients, thus questioning the usefulness of these markers in monitoring (short-term) adherence to the diet (Berry et al 1993; Bosch et al 2004a; Coss et al 2012). It also has been reported that blood Gal-1-P and urinary galactitol have a high biological variability with high inter- and intra-individual variation, making single measurements of little value (Hutchesson et al 1999). Gal-1-P is useful in detecting gross dietary deviations and acute intoxication (Bosch et al 2004a). There is (limited)

evidence that monitoring of galactosylation may be an effective parameter in the future (Coss et al 2012; Knerr et al 2015). At this time Gal-1-P measurement, using each patient as his own reference, appears the best parameter for monitoring patients. There is, however, a strong need for improved monitoring biomarkers.

Recommendation #8 (++)

In the first year of life clinicians should measure red blood cell Gal-1-P levels at diagnosis, and after 3 and 9 months of dietary galactose restriction.

Recommendation #9 (expert opinion, +)

We recommend measuring red blood cell Gal-1-P levels yearly after the first year of life until an individual baseline has been established.

Recommendation #10 (expert opinion, +)

We recommend measuring red blood cell Gal-1-P levels in case of increase in galactose intake and concern about intoxication.

Recommendation #11 (expert opinion, +)

The clinical utility of serial blood or urinary galactitol measurement is limited.

Long-term complications

Cognitive development

Despite initiation of a lactose- and galactose-restricted diet early in life, patients are at risk for decreased intellectual ability. Therefore, intellectual quotient (IQ) tests are frequently performed in the follow-up of patients with CG. IQ scores can also serve as a baseline for interpreting deficits in other areas; for example, poor executive functioning is common in children with low IQ. In addition, periodic evaluation of cognitive abilities can serve to assess the following: adequacy of treatment in preventing cognitive decline, the risk for developmental delay in specific domains (such as verbal abilities, reasoning abilities, memory, and processing speed), and the potential need for early intervention and special education services. In CG, most studies report poor cognitive outcomes with mean IQ scores below average or in the low average range, but there is considerable variability between individual patients and scores range from very low to above average (Donnell et al 1961; Komrower and Lee 1970; Waggoner et al 1990; Schweitzer et al 1993; Kaufman et al 1994; Kaufman et al 1995; Hansen et al 1996; Rasmussen et al 1996; Badawi et al 1996; Shield et al 2000; Widhalm et al 2002; Antshel et al 2004; Doyle et al 2010; Schadewaldt et al 2010; Coss et al 2013; Rubio-Agusti et al 2013). The reported percentage of patients with an IQ score below 85 varies from 45 to 72 % (Schweitzer et al 1993; Rasmussen et al 1996; Shield et al 2000; Hoffmann et al 2011). A subject of debate in the follow-up of CG is whether the cognitive impairment is progressive. Some studies report a negative correlation between age and performance (Komrower and Lee 1970; Waggoner et al 1990; Doyle et al 2010). This finding may be an artifact of these studies' cross-sectional design and not measuring scores within the same patients over time. Other cross-sectional studies reported no significant difference in IQ scores between older and younger patients (Hoffmann et al 2011; Coss et al 2013). In longitudinal studies, no deterioration of cognitive function over time was reported during childhood/adolescence (Fishler et al 1966; Waggoner et al 1990; Manis et al 1997) and adulthood (Waggoner et al 1990; Schadewaldt et al 2010). A number of studies report significantly lower IQ scores in females compared to males (Komrower and Lee 1970; Waggoner et al 1990), yet other studies could not confirm this finding (Fishler et al 1966; Kaufman et al 1995; Hoffmann et al 2011). Mean IQ scores and range of IQ for patients with DG (aged 1–6 years), both on an early-initiated lactose-free diet and on a regular diet, were found to be comparable to the general population (Ficicioglu et al 2008).

Recommendation #12 (++)

Clinicians should refer patients for testing of developmental quotient (DQ) and intellectual quotient (IQ), to obtain a well-validated measure of development and cognitive abilities. At minimum, testing should be done at:

Age 2–3 years: to assess early speech/language and motor development in time for early intervention, using a standardized test instrument such as the Bayley Scales of Infant and Toddler Development (BSID) or a similar measure.

Age 4–5 years: to assess school readiness and need for occupational therapy and speech-language therapy, using a standardized test instrument such as the Wechsler Preschool and Primary Scale of Intelligence (WPPSI) or a similar measure.

Age 8–10 years: to assess cognitive development, specific areas of strengths and weaknesses, and the need for special therapies, using a standardized test instrument such as the Wechsler Intelligence Scale for Children (WISC) or a similar measure.

Age 12–14 years: to assess cognitive development and specific areas of strengths and weaknesses, and to assess the need for special therapies, using a standardized test instrument such as the Wechsler Intelligence Scale for Children (WISC) or a similar measure.

Age 15 years and older: according to needs, specific questions.

(consider combining these assessments with speech and language screening, recommendation #15, and psychosocial development screening, recommendation #21)

Recommendation #13 (expert opinion, +)

For obtaining a measure of functioning when formalized testing is not possible or when additional assessments are needed between formalized testing points, we recommend using a validated parent/informant questionnaire, such as the Adaptive Behavior Assessment System (ABAS) or a similar measure.

There is no correlation between IQ and the time of initiation of dietary treatment as long as treatment is started within the first 8 weeks of life (Schweitzer et al 1993; Schadewaldt et al 2010). Correlation with genotype is unclear as some studies suggest that IQ is not correlated to the p.Q188R variation (Lee 1972; Kaufman et al 1994; Cleary et al 1995), while another study reports that patients with homozygosity for p.Q188R have lower IQ scores than patients with less common genotypes (Shield et al 2000).

Executive functions Only a few studies have evaluated the executive function of patients with CG. Mean scores of executive functioning in adult patients with CG are below the average, but there is considerable variability between individual patients (Doyle et al 2010). Overall, 15 % of adult patients demonstrate deficits in executive functioning as self-reported on the Behavior Rating Inventory of Executive Function (BRIEF) (Waisbren et al 2012). As evidenced by direct evaluation of children and parent responses on the BRIEF, children with CG exhibit less well-developed executive functioning compared to peers (Antshel et al 2004). Sustained attention and information processing may also be impaired (Widhalm et al 2002).

Recommendation #14 (expert opinion, +)

We recommend a clinical assessment of executive function, if feasible in the clinic, with specific attention to processing speed and visual spatial comprehension. In children (8–10 years) as a first screening use the Behavior Rating Inventory of Executive Function (BRIEF), and in adolescents (12–14 years) and in young adults (18–20 years) use the Cambridge Neuropsychological Test Automated Battery (CANTAB), the Amsterdam Neuropsychological Tasks program (ANT) or a similar measure, with follow-up, as needed.

Speech and language impairment

Various speech and language disorders have been reported in CG. Language is the tool by which we communicate thoughts, feelings, and ideas either spoken or written, and speech is the tool by which we verbally communicate with others. In some reports on speech and language disorders in CG the disorder is well-defined such as childhood apraxia of speech (also called developmental verbal dyspraxia, a motor speech disorder with problems saying sounds, syllables, and words), articulation disorders, dysarthria, and receptive language disorders. In many reports more general speech/language delays or disorders are reported, relating primarily to producing rather than understanding speech and language. Few studies have used standardized and validated test instruments, but overall 24–88 % of children and adults with CG are reported to have a speech and language disorder (Waggoner et al 1990; Nelson et al 1991; Schweitzer et al 1993; Hansen et al 1996; Rasmussen et al 1996; Webb et al 2003; Potter et al 2008; Hughes et al 2009; Hoffmann et al 2011; Shriberg et al 2011; Waisbren et al 2012; Coss et al 2013; Rubio-Agusti et al 2013). Speech motor function may be affected as well, specifically reduced tongue strength (73 %), decreased breath support for speech (32–64 %) (Waisbren et al 2012; Potter et al 2013), and disturbed vocal quality to laryngeal insufficiency (33 % of children with CG and speech disorders) (Potter 2011). Almost 10 % of patients with CG are affected by vocal tremors of unknown origin (Potter 2011). Children with CG and a history of speech disorders also have a four- to sixfold greater relative risk for co-occurring language disorders (Potter et al 2008). Patients with CG show difficulties in language production tasks, both behaviorally (less accurate and slower) and in their brain's signature measured by functional magnetic resonance imaging (fMRI) and by event-related potentials (ERPs), compared to healthy controls. The ERP differences continue throughout consecutive linguistic preparation phases, which indicates an affected lexical access and impaired syntactic planning (Timmers et al 2012), while the fMRI findings point toward both affected linguistic preparation and motor speech planning (Timmers et al 2015a). Many individuals with CG (up to 86 %) have received speech therapy, with most children receiving direct speech therapy from a speech and language therapist or, less frequently, indirect speech therapy with the speech and language therapist working with the child's family (Waggoner et al 1990; Schweitzer et al 1993; Potter et al 2008; Timmers et al 2012). There is no evidence in the literature addressing therapeutic options or therapeutic efficacy. In CG, language disorders and type of language disorder are associated with, but not fully explained by, cognitive function (Waggoner et al 1990; Nelson et al 1991; Hoffmann et al 2011). More than half (56 %) of patients with CG with average cognition and most (88 %) patients with CG with borderline-low cognition have co-occurring speech and language disorders (Potter et al 2008). Nelson et al (1991) reported that the presence or severity of CAS is not related to age at start of diet, the presence of neonatal symptoms, gender or age at the time of speech evaluation. Another study reported that the number of days consuming milk prior to diagnosis is associated with poorer speech outcomes in males, not females, with CG (Potter et al 2013).

Patients with the p.Q188R/p.Q188R genotype are reported to be at greater risk for speech and language impairment than participants with p.Q188R/other genotypes (Potter et al 2008, Robertson et al 2000). Two-thirds of children with CG and speech disorders have co-occurring coordination disorders and children with CG and CAS or dysarthria have poorer balance and manual dexterity (Potter et al 2013).

Recommendation #15 (++)

All children with CG should be screened for speech and language delay at ages 7–12 months, 2, 3, and 5 years (consider combining with screening for cognitive disorders, recommendation #12). If children show low or borderline speech and language development, full assessments should be conducted.

Recommendation #16 (expert opinion, +)

We recommend that an assessment of speech and language includes hearing screening, a brief assessment of pre-linguistic communication (<2 years of age) and expressive, receptive, and pragmatic language use, structure-function examination, motor speech (observation of respiration, resonance, voice, articulation), and speech intelligibility for all children not meeting age appropriate milestones. We recommend a cognitive evaluation as well if a disorder is suspected.

Recommendation #17 (expert opinion, +)

For children who are not meeting age appropriate speech or language milestones, we recommend treatment based on guidelines for treatment of speech, language, and voice disorders in the general population. Therapy should begin during the first year of life and include modeling and training of gestural communication to increase infant and toddler language development. Play-based milieu for language development is recommended during the second year of life. Individual speech therapy focused on high repetition of a small number of targets should begin during the second year of life and continue as needed throughout the preschool and elementary school years. Respiration, phonation, and resonance deficits should also be addressed.

Neurological complications

Patients with CG are at risk for central nervous system dysfunction, not only manifesting itself as neurological symptoms and motor problems, but also as cognitive impairment, speech and language problems, and psychiatric symptoms. The latter three are discussed in a separate part of this guideline.

Affected newborns may develop encephalopathy and signs of increased intracranial pressure with cerebral edema after ingestion of galactose (Huttenlocher et al 1970; Belman et al 1986; Najafi et al 2005). In the long-term it is known that patients may develop neurological complications, with an overall frequency of motor dysfunction of up to 66 % (Hughes et al 2009; Milánkovics et al 2010; Rubio-Agusti et al 2013; Viggiano et al 2015). The studies addressing specific neurologic symptoms all report on different symptoms and have very heterogeneous populations with large variation in sample sizes, making it difficult to reliably estimate the percentage of patients that suffer from each of these symptoms. Frequently noted signs are mild to severe ataxia, tremor, dystonia, dysarthria, and dysmetria (Waggoner et al 1990; Nelson et al 1992; Schweitzer et al 1993; Kaufman et al 1994; Kaufman et al 1995; Dubroff et al 2008; Krabbi et al 2011; Waisbren et al 2012; Coss et al 2013; Rubio-Agusti et al 2013). Epilepsy is reported in a minority of patients (Schweitzer et al 1993; Krabbi et al 2011; Rubio-Agusti et al 2013; Aydin-Özemir et al 2014). One study, that followed 22 patients with CG diagnosed early, did not find any cases with ataxia or dysmetria in young patients aged 0–6 years (Karadag et al 2013). One study reported eye movement abnormalities and pyramidal signs in some patients (including brisk tendon reflexes and clonus, extensor plantar response, spastic paraparesis, and pseudobulbar signs) (Rubio-Agusti et al 2013).

Recommendation #18 (++)

Clinicians should screen patients with CG for neurological involvement by clinical examination from the age of 2–3 years. Such screening should include examination for ataxia, tremor, dysmetria, and dystonia. If a specific neurological deficit is noted, monitoring of progression with a designated scale is advised. It is suggested to screen adult patients annually and to record progression, if any. Pediatric patients could be screened more frequently (every 6 months) in order to identify potentially modifiable neurological problems.

Recommendation #19 (+)

We recommend asking patients or caregivers about onset of seizure and seizure-like activity since previous examination and perform an EEG, if indicated.

Cerebral imaging An abnormal white matter signal is present in the majority of MRI scans (>75 %) of patients with CG, indicative of abnormal myelination (Nelson et al 1992; Kaufman et al 1995; Moller et al 1995; Wang et al 2001; Hughes et al 2009; Krabbi et al 2011). Additional reported MRI findings are focal white matter lesions, white matter volume loss, (mild) cerebral atrophy, enlargement of the fourth ventricle and cerebellar sulci (suggesting cerebellar atrophy), and enlargement of lateral ventricles (Nelson et al 1992; Kaufman et al 1995; Moller et al 1995; Wang et al 2001;

Hughes et al 2009; Krabbi et al 2011; Rubio-Agusti et al 2013; Timmers et al 2015b). One study followed patients over time with MRI scans (Nelson et al 1992). All eight patients younger than 1 year of age had normal white matter signaling. All patients over the age of 1 year either had an abnormal peripheral myelin pattern, or developed abnormalities within 1–2 years without apparent progression in time. From this study 22/63 patients had mild lateral ventricular enlargement at the initial MRI. This enlargement was unchanged in four patients that had follow-up MRIs 1–2 years later. Of the 20/63 patients without lateral ventricular enlargement that had follow-up examinations, none developed lateral ventricular enlargement. In some studies only patients with neurological symptoms had MRI examination, and in other studies clinical status was not reported. It is unclear if the abnormalities on MRI-scans are representative for the whole population of patients with CG and if the findings are correlated with clinical symptoms. One study also performed magnetic resonance spectroscopy (MRS) in addition to brain MRI to assess in vivo brain metabolism (Moller et al 1995). MRS showed a normal spectrum of metabolites, with no indications of elevated Gal-1-P levels or an impairment of energy supply in the brain. No relationship between MRS and clinical data was found. A study on white matter microstructure pathology using neurite orientation dispersion and density imaging (NODDI) showed extensive white matter abnormalities with a lower neurite density index and increased orientation dispersion index in the regions involved with higher order cognitive functions and language and motor functions (Timmers et al 2015b).

Recommendation #20 (expert opinion, +)

We do not recommend routine brain and spinal cord imaging in the follow-up of patients with CG. In those patients with significant or progressive neurological symptoms and signs, imaging may be warranted to (1) determine if a second condition is present or (2) further define the development and progression of neuroradiology findings in individual patients.

Psychosocial development

Due to the chronicity of the disease, the need to adhere to a life-long diet and the impact of the long-term complications, patients with CG are at risk for problems in psychosocial development, including personality, social relationships, and emotional well-being.

Psychosexual and social development, marital status Specific testing of social and psychosocial milestones in adult patients showed that patients achieved fewer developmental milestones in the psychosexual and social domain (having friends and engaging in social activities) (Bosch et al 2009; Gubbels et al 2011a). Multiple studies assessed marital status in adult patients with CG, with the percentage of patients married or living in stable partnership varying between 13 and 57 % (Bhat et al 2005; Bosch et al 2009; Waisbren et al 2012; Hoffmann et al 2012). Bosch et al 2009 reported that the percentage of patients married (14.3 %) was significantly lower compared to the reference population (39.1 %). Hoffmann et al 2012 found a difference in marital status between sexes with more married females (57 %) compared to males (11 %), with the percentage of married males much lower than in the reference population (47.2 %). In one study assessing males only, 5 % of patients were married, compared to 30.9 % in the reference group of males that did not differ in age, though this difference was not statistically significant (Gubbels et al 2011a). Few patients with CG had children (0–5 %), while a desire to have children was reported by nearly half of patients (Bhat et al 2005; Hoffmann et al 2012). The percentage of patients actually trying to conceive a child or the percentage successful was not reported. While primary ovarian insufficiency (present in the majority of female patients) might be an important contributing factor to the minority of females having children, there is no evidence of decreased fertility in males (see the Endocrinology and fertility part of this guideline) (Rubio-Gozalbo et al 2006; Gubbels et al 2011a). The percentage of patients trying to conceive might also be low, due to the fact that fertility counselling in the recent past was often focused on the expected low chances of achieving pregnancy.

Educational attainment and employment Bosch et al reported that 44 % of children with CG, aged 6–11 years, attended special schools as opposed to 3 % of the general population (Bosch et al 2004b). Educational attainment was significantly lower than the general population with 61.5 % completing basic school and low vocational training only, compared with 27.2 % of the general population (Bosch et al 2004b). Lower education attainment was confirmed in another study which reported that fewer individuals with CG (10.8 %) had a school leaving certificate, compared to 3.5 % in the general population, and a minority achieved a university entrance diploma (8.1 %) compared to the general population (28.6 %). However, a higher percentage of individuals with CG (81 %) earned a secondary school degree compared to the general population (59.5 %) (Hoffmann et al 2012). In adulthood, up to 30 % of individuals with CG were unemployed, with no differences between males and females (Bosch et al 2009; Waisbren et al 2012; Hoffmann et al 2012). A recent survey of 60 adult patients with galactosemia in the UK revealed that 58 % were in paid employment, compared with 74 % of the general population (unpublished data, Charles Dent Metabolic Unit, UK, 2015).

Psychiatric symptoms and emotional problems Patients with CG have been reported to suffer more frequently from

psychiatric symptoms and emotional disturbance, including depression, anxiety, obsessive-compulsive disorder, and autism spectrum disorder (Lee 1972; Rubio-Agusti et al 2013). Parents of children with CG report their children exhibit more internalizing symptoms (e.g., depression and anxiety) without elevated levels of dysphoric mood compared to controls (Antshel et al 2004). Depression (as detected with the Beck Depression Inventory), was present in 12 % of adult patients, and anxiety (as measured with the Beck Anxiety Inventory) was present in 52 % (Waisbren et al 2012).

Coping with CG Coping with CG by patients was assessed in two studies, both using condition specific but non-validated questionnaires. It was demonstrated that over 75 % of the patients rated their coping with CG as 'very good' or 'good'. The remainder had difficulties in coping with their condition. CG was seen as a burden by 39 % of patients (Bosch et al 2004b; Hoffmann et al 2012). Many patients (42 %) had a problem maintaining the diet, and patients reported that 'diet/nutrition' was the primary aspect of life influenced by galactosemia, followed by 'school/work' and 'friends/leisure'. Many parents of patients with CG (60 %) considered it a burden to take care of a child with CG, and many believed that CG influenced their relationship with their child. More than half of parents of girls frequently worried about possible infertility. However, a high percentage of parents (86 %) believed that one could live a good life with this disorder (Bosch et al 2004b) and only 7.7 % of adults with CG reported that galactosemia had a negative effect on family life (Hoffmann et al 2012).

Screening for psychosocial deficits Currently no universal and validated checklist is available to screen for psychosocial deficits.

Recommendation #21 (expert opinion, +)

We recommend screening children for psychosocial deficits, including autism spectrum disorders, sensory integration problems, depression and anxiety, using standardized questionnaires such as the Behavior Assessment System for Children, Second Edition (BASC-2) in English or a similar tool in other languages. We recommend performing this screening at age 2 years in combination with screening for speech and language delays (see recommendation #15) and to combine this screening with developmental testing at ages 4–5, 8–10, and 12–14 years (see recommendation #12).

Recommendation #22 (+)

We recommend screening adults for mental health issues with validated questionnaires that include brief scales for anxiety and depression, such as the NIH PROMIS Questionnaires, Beck Anxiety Inventory (BAI), Beck Depression Inventory (BDI) or similar measures. With adults, we recommend discussing living situations, work and/or educational situations, satisfaction with social relationships, and sexual intimacy during outpatient clinic visits and to refer for professional consultation, if necessary.

Health-related quality of life (HRQoL) Three studies report on HRQoL in patients with CG. One study demonstrated that having CG negatively affects HRQoL of both children and adults (Bosch et al 2004b). Children aged 6–15 years had a lower HRQoL in the domains of cognitive function and of motor function. Patients >16 years of age reported significantly lower scores in the domains of cognitive function and social function. Hoffmann et al showed that adult patients with CG scored significantly lower on the domains 'positive mood' and 'social well-being' when compared to the general population and compared to PKU patients (Hoffmann et al 2012). Finally, a study evaluating HRQoL in patients >6 years of age reported that patients with CG did not differ from their peers in their physical activities, mobilization, overall health, and their self-esteem, but that they did have difficulties in their relationships with others. Despite feeling 'not as good as most people', all patients had been happy at some point in the 4 weeks preceding the interview (Lambert and Boneh 2004). The HRQoL of parents of children with CG did not differ from the HRQoL of parents of healthy children (ten Hoedt et al 2011).

Recommendation #23 (expert opinion, +)

We do not recommend routine health-related quality of life (HRQoL) evaluations.

Fertility Over 80 % of females with CG develop primary ovarian insufficiency (POI) (Kaufman et al 1981; Kaufman et al 1988; Waggoner et al 1990; Sanders et al 2009; Rubio-Gozalbo et al 2010; Fridovich-Keil et al 2011; Coss et al 2013; Rubio-Agusti et al 2013). The clinical spectrum of POI in women with CG varies from primary amenorrhea with or without lack of development of secondary sexual characteristics, to normal pubertal development followed by irregular menses, oligomenorrhea or secondary amenorrhea. Most patients experience subfertility, and often show a diminished ovarian reserve. Many females need puberty induction and/or hormone replacement therapy to prevent sequelae of POI (Gubbels et al 2008). The mechanisms underlying POI and the timing of the ovarian damage in CG are not understood to date. Possible underlying pathophysiological mechanisms of POI in CG include direct toxicity of accumulated galactose or one of its metabolites, abnormal glycosylation of glycoproteins or glycolipids (including hormones such as follicle stimulating hormone (FSH)), and wrongful activation of follicular apoptosis (Liu et al 2000; Forges et al 2006; Rubio-Gozalbo et al

2010; Fridovich-Keil et al 2011). One study reported that female patients with CG have additional FSH isoforms besides normal acidic FSH isoform, (Prestoz et al 1997), but another study did not confirm these findings (Gubbels et al 2011b). FSH-inactivity seems not to be a probable cause of POI, as most female patients with CG do not respond significantly to stimulation with exogenous FSH and/or luteinizing hormone (LH) (O'Herlihy and Danks 1985; Gubbels et al 2013a).

Biomarkers for POI Corresponding to the incidence of POI, most (>80 %) female patients with CG demonstrate raised levels of FSH (Waggoner et al 1990; Guerrero et al 2000), while estradiol levels are decreased in adolescent girls and women (Rubio-Gozalbo et al 2006). Levels of anti-Müllerian hormone (AMH), a marker for ovarian reserve which is produced by granulosa cells of (healthy) pre-antral and small antral follicles of the ovary (Broer et al 2014), are reported to be abnormally low among girls and women with CG across all age groups, even in patients <1 years of age (Sanders et al 2009; Gubbels et al 2013a; Spencer et al 2013). However there is no significant difference in AMH levels between girls with CG with spontaneous menarche compared to hormone replacement therapy assisted menarche (Spencer et al 2013). Female patients with CG demonstrated normal gonadotropin levels that increase as POI becomes apparent, normal levels of prolactin, total cholesterol, triglycerides, high-density lipoprotein (HDL) cholesterol, low-density lipoprotein (LDL) cholesterol, thyroid-stimulating hormone (TSH), thyroid-binding globulin (TBG), free thyroxine (FT4), basal testosterone, free testosterone, andostenedione, and dehydroepiandrosterone sulfate(DHEAS) (Kaufman et al 1981; O'Herlihy and Danks 1985; Rubio-Gozalbo et al 2006). Also, no ovarian antibodies were found (Kaufman et al 1981).

Imaging Ovaries of girls and women with CG measured on MRI were significantly smaller when compared to age-matched controls, but did not differ significantly from post-menopausal controls (Gubbels et al 2013a). In more than half of patients with CG ovaries could not be visualized on ultrasound, compared to 10 % in healthy controls. On ultrasound most patients had antral follicle counts below the control range (Spencer et al 2013).

Recommendation #24 (++)

Girls with CG should be screened for hypergonadotropic hypogonadism if they reach the age of 12 years with insufficient secondary sex characteristics or if they reach the age of 14 years with no regular menses. Screening should include follicle-stimulating hormone and 17-beta-estradiol.

Recommendation #25 (expert opinion, +)

We recommend considering follicle stimulating hormone level, growth, and psychosocial maturity of the individual girl, for determination of age at start of treatment. For puberty inducement, a low dose estrogen in a step-wise escalating dose is used, then later combined with cyclic progesterone for regular withdrawal bleeds. We recommend considering referral to a pediatric endocrinologist.

Recommendation #26 (expert opinion, +)

We recommend not using anti-Müllerian hormone and ovarian imaging routinely for follow-up as these have not been shown to accurately predict pubertal development or fertility outcome.

Recommendation #27 (+)

We do not recommend endocrine follow-up for Duarte Galactosemia, as there is no evidence that the ovaries are affected.

Recommendation #28 (expert opinion, +)

We recommend that girls and women with CG, who have gone through puberty and established regular menstrual periods, should be monitored annually for menstrual abnormalities, secondary amenorrhea, and symptoms of primary ovarian insufficiency (POI). Changes in menses or POI symptoms should be evaluated with a serum follicle-stimulating hormone level. Anti-Müllerian hormone measurement is not helpful in determining which women will undergo POI, but may be helpful in identifying women at risk for imminent POI when it is undetectable. Imaging by pelvic ultrasound or MRI is not recommended unless otherwise clinically indicated.

Recommendation #29 (expert opinion, +)

We recommend that women with hypergonadotropic hypogonadism, or primary ovarian insufficiency should be provided counseling and support about their reproductive options and management of irregular or absent menses. Hormone replacement therapy should be initiated with the onset of secondary amenorrhea to reduce the risk of osteoporosis and other complications of primary ovarian insufficiency.

Recommendation #30 (++)

We recommend considering a referral to a reproductive endocrinologist for women who desire pregnancy and have been unable to conceive naturally, or for women who desire

additional counseling about fertility treatment options including oocyte donation.

Recommendation #31 (expert opinion, +)

We recommend providing counseling about adequate birth control methods for women who do not desire pregnancy. While combined oral or transdermal contraceptives may provide cycle control, bone protection, and attenuate hot flashes, they may fail to provide adequate birth control in women with very elevated follicle-stimulating hormone levels. An intrauterine device may provide the lowest failure rate.

Risk factors for POI The risk of POI in CG is positively associated with higher mean Gal-1-P levels after 1 year of dietary treatment, reduced galactose oxidation capacity, and homozygosity for the p.Q188R variation (16-fold increased risk) compared to heterozygosity for this variation or two different variations (Guerrero et al 2000). Homozygosity for the p.Q188R variation, however, is not predictive for the development of primary amenorrhea versus secondary amenorrhea (Kaufman et al 1994). Because of the high frequency of the mild p.S135L variation in African American patients, there is an association between ethnicity and the outcome of POI, with a high proportion of the Caucasian females but no African American females diagnosed with POI (Guerrero et al 2000). Residual GALT activity might be a modifier of ovarian function as well, as female patients with >0.4 % predicted wild-type GALT activity are more likely to show AMH levels of >0.1 ng/ml when compared to girls with <0.4 % GALT activity (Spencer et al 2013). There is no association between POI and the age at initiation of dietary treatment, degree of dietary control, highest erythrocyte Gal-1-P level (Guerrero et al 2000), and urinary galactitol levels (Kaufman et al 1981).

Pregnancy, counselling and fertility preservation Chances of pregnancy are reduced in POI, but pregnancies in women with CG have been reported, and appear not to be as rare as is generally assumed (reviewed by Gubbels et al 2008). As recommendations on fertility preservation were lacking, Van Erven et al issued recommendations for physicians based on current knowledge concerning galactosemia and fertility preservation (Van Erven et al 2013). Oocyte donation may be used to establish pregnancy in women with CG and POI, but has some psychological disadvantages (Sauer et al 1991). Three fertility preservation techniques are currently offered to patients in need of fertility preservation: ovarian tissue, mature oocyte and/or embryo cryopreservation. The application of fertility preservation in these patients is complicated however, because the underlying mechanisms and onset of POI in CG are not fully understood. Also the experience with fertility preservation is mainly derived from cancer patients with previously unaffected ovaries, and some procedures like cryopreservation of ovarian tissue are still experimental (Van Erven et al 2013).

Recommendation #32 (expert opinion, +)

Fertility preservation may not be successful. Currently, fertility preservation techniques are not yet readily used in everyday practice. We recommend fertility preservation should only be offered with appropriate institutional research ethics review board approval to girls with classical galactosemia at a young pre-pubertal age.

Males In contrast to female patients, fertility does not seem to be affected in males, but there is a paucity of data about reproductive function and male patients fathering children, possibly due to psychosocial rather than biological reasons. One study reports a confirmed delayed onset of puberty in one of 18 males over 12 years of age (Schweitzer et al 1993). A higher rate of cryptorchidism (Rubio-Gozalbo et al 2006; Gubbels et al 2011b), lower semen volumes as a group, and sperm concentrations in individuals are reported (Gubbels et al 2011b). Males demonstrated normal levels of gonadotropins, total cholesterol, triglycerides, HDL cholesterol, LDL cholesterol, prolactin, TSH, TBG, FT4, basal testosterone, free testosterone, androstenedione, and DHEAS (Rubio-Gozalbo et al 2006; Gubbels et al 2013b).

Recommendation #33 (+)

We do not recommend routine endocrinology follow-up in males.

Bone health

Patients with CG might be at risk for impaired bone health due to various reasons, such as restrictions in dietary intake, decreased physical activity in some patients, POI in females, and currently unknown pathophysiologic factors intrinsic to the disease. The preferred and most frequently used method to measure BMD in children is dual-energy X-ray absorptiometry (DXA) (Crabtree et al 2014). Other types of bone densitometry measurements, such as quantitative computed tomography (CT) and quantitative ultrasound, are also available. BMD can be measured at different sites and is frequently reported as a T- or Z-score, which are both units of standard deviation. In children the preferred sites to measure BMD are the lumbar spine and the total body less head (TBLH); in adults the lumbar spine, total hip and/or femoral neck (ISCD 2015). Two-dimensional cross-sectional area measurements (areal BMD (aBMD)) or estimated volumetric BMD

(vBMD) measurements can be performed. The diagnosis of osteoporosis in children, premenopausal women, and males under 50 years of age should be based on densitometric criteria combined with fracture history. The definition of osteoporosis for these groups is a BMD Z-score < -2 ('BMD below the expected range for age' or 'low BMD for chronological age') combined with a significant fracture history (Crabtree et al 2014).

Bone health in CG Three studies in adult patients report vBMD Z-scores of −1.9 and −1.4 in males. (Kaufman et al 1993); aBMD Z-scores −1.19 in females (lumbar spine) and −1.25 (total hip) in females and −0.80 (lumbar spine) and −0.81 (total hip) in males (Batey et al 2013); and BMD Z-scores greater than 2 standard deviations below the normative mean in 8 of 33 patients (Waisbren et al 2012).

Four studies reported on BMD Z-scores in children and adolescents, three using DXA scanning BMD (Rubio-Gozalbo et al 2002; Panis et al 2004; Doulgeraki et al 2014) and one using quantitative CT scanning (Kaufman et al 1993). Two studies demonstrated decreased lumbar spine aBMD Z-scores (−0.65 and −0.6) (Panis et al 2004; Doulgeraki et al 2014), and two other studies found decreased total body aBMD Z-scores (−0.3 and −0.99) (Rubio-Gozalbo et al 2002; Doulgeraki et al 2014). Panis et al 2004 and Rubio-Gozalbo et al 2002 found decreased femoral neck vBMD Z-scores as well (−0.28 and −1.76). It should be noted that recent insight is that Z-scores should not be used for volumetric measurements. Kaufman et al found a significantly decreased mean BMD of the lumbar spine (assessed with quantitative CT) when compared to age-matched healthy controls, in both children and adult patients (Kaufman et al 1993). Karadag et al demonstrated normal BMD in all patients aged 0–6 years, tested with DXA and quantitative ultrasound, but decreased BMD was not defined (Karadag et al 2013). Coss and colleagues demonstrated osteopenia or osteoporosis (in children >10 years old and adults) in 14.7 % of the Traveller population and in 39.6 % of the non-Traveller population, using Z-scores generated from DXA scans, but it is unclear how the authors defined osteopenia and osteoporosis (Coss et al 2013). A randomized controlled trial of 2 years in children with CG evaluated the effect of calcium and vitamin K1 and D3 supplementation versus placebo on bone mineral content (BMC), and showed a significant increase in BMC of lumbar spine in the treatment group compared to placebo group, but only in prepubertal children (Panis et al 2006b). Self-report of fractures in adult patients with CG showed that 63 % of women and 31 % of men sustained at least one lifetime fracture (Batey et al 2013), which seems to be comparable to the general population (Jones et al 2002). However, further studies will need to validate these results.

Recommendation #34 (++)

Clinicians should assess bone mineral density (BMD) by age appropriate dual-energy X-ray absorptiometry (DXA) scan.

Recommendation #35 (expert opinion, +)(consensus: 93 %)

We recommend BMD screening from age 8–10 years. With evidence of reduced bone density (Z-score ≤ -2.0), follow-up according to current pediatric bone health guidelines is advised. Without evidence of reduced bone density, we recommend performing a repeat dual-energy X-ray absorptiometry scan when puberty is complete. We recommend performing follow-up thereafter every 5 years and treatment instituted according to WHO FRAX recommendations.

Recommendation #36 (+)

We recommend comprehensive dietary evaluation, optimization of calcium intake if needed, monitoring and if necessary supplementation of vitamin D, hormonal status evaluation and hormone replacement therapy consideration, as well as regular exercise and assessment of skeletal problems and clinically significant fractures in all patients with CG. Supplementation of vitamin K might be beneficial when combined with an adequate intake of calcium and vitamin D, but currently there is not enough evidence to recommend the routine use of vitamin K.

Bone metabolism Measurement of bone metabolism parameters, such as minerals, vitamins, hormones, bone formation markers, and bone resorption markers, might be helpful in understanding underlying mechanisms of a decreased BMD and evaluation of treatment (e.g., supplementation of calcium and vitamin D). Studies measuring these parameters in patients with CG have only been performed in children and adolescents. Levels of carboxylated osteocalcin, N-terminal telopeptide, C-terminal telopeptide, and IGF-1 Z-scores were found to be significantly decreased in patients with CG, while values of bone alkaline phosphatase, osteocalcin, and C-terminal telopeptide were found to be higher than in controls (Rubio-Gozalbo et al 2002; Panis et al 2004; Gajewska et al 2006; Gajewska et al 2008). All other tested parameters in these four studies were in the reference range or comparable to healthy controls.

Recommendation #37 (expert opinion, +)

At present there is not enough evidence to justify routine determination of bone turnover markers in patients with CG.

Cataracts

Cataracts are a frequently encountered complication of CG, mainly in the newborn period. Cataracts always occur bilaterally, and the enhancement of nuclear or perinuclear refractive power leads to the appearance of a refractile ring or a drop of oil in the crystalline center, which is an early sign. Later stages appear as nuclear or zonular opacities, and in all stages vacuoles might be present. The cause of cataract is accumulation of galactitol in the crystalline lens, the result of activation of the aldose reductase shunt (Widger et al 2010). Cataracts are typically already present in the first weeks of life, with a prevalence which varies from 6 to 25 % across several studies (Kossowicz and Zbieg Sendecka 1977; Burke et al 1988; Burke et al 1989; Badawi et al 1996). In a retrospective case series, 14/100 patients with CG were diagnosed with cataracts, with the average age at cataract diagnosis of 6.3 years (Widger et al 2010). However, this article did not report on age of diagnosis, previous ophthalmologic examinations or dietary adherence. A study on long-term complications in adulthood reported the presence of cataracts in 21 % of adult patients with CG, as noted in medical records or reported during the medical history (Waisbren et al 2012). Overall prevalence of cataracts, regardless of age at start of diet, is extremely variable across different studies, with the larger retrospective case series reporting 7.7 % (Coss et al 2013) and 14 % (Widger et al 2010). A smaller study reported cataract in 17/22 patients, including 13/18 patients who were diagnosed before 17 days, and in 4/4 patients who were diagnosed after 17 days. One prospective case series with a mean follow-up period of 8.5 years reported an overall prevalence of cataracts of 36 % in 33 patients (Beigi et al 1993), but the time of detection of the cataracts was not reported, except for two patients in this cohort who developed reversible cataract at the age of 2.5 and 3.7 years respectively, both after a 3-month period off diet (Burke et al 1988; Burke et al 1989; Beigi et al 1993).

Severity of the cataracts reported in patients with CG varies, but in the vast majority of cases visual acuity is not affected, and lens opacities frequently resolve spontaneously over time in patients on a galactose-restricted diet (Kossowicz and Zbieg Sendecka 1977; Burke et al 1988; Burke et al 1989; Waggoner et al 1990; Beigi et al 1993; Schweitzer et al 1993; Badawi et al 1996). Reports of cataract necessitating surgery are rare: one study reports on a 33 year old male suffering from blindness due to cataract, but age of diagnosis and dietary adherence were not reported (Schweitzer et al 1993). Karadag et al reported four patients diagnosed after age 17 days required cataract surgery (Karadag et al 2013). Waggoner et al reported eight cases (out of 314 patients) requiring surgery, including one patient that had been treated from birth but with unknown adherence (Waggoner et al 1990).

Widger et al could not demonstrate a direct relationship between dietary adherence and cataract formation, however in this study dietary nonadherence was defined by the relatively low galactose intake of >50 mg/day (Widger et al 2010). Levels of Gal-1-P have not been demonstrated to correlate with cataract formation (Beigi et al 1993).

The current available literature is inconclusive regarding which patients will develop cataracts and at what age, and if adherence to the diet plays a direct role in the development of cataracts. There are however strong suggestions that cataracts in the neonatal period usually do not affect visual acuity and, with dietary adherence, will often resolve spontaneously. Furthermore it seems that, patients with a good dietary adherence do not develop cataracts later in life or if they do develop cataracts, visual acuity is not affected.

Recommendation #38 (++)

Clinicians should refer all patients to an ophthalmologist for evaluation of cataract at the time of diagnosis.

Recommendation #39 (+)

We recommend performing ophthalmological follow-up in patients with a cataract at diagnosis until it has fully resolved.

Recommendation #40 (+)

We recommend performing ophthalmological screening in all patients who are non-compliant with diet.

Closing remarks

The presented guideline is the first international and evidence-based guideline for the diagnosis, treatment, and follow-up of CG, and aimed to be applicable worldwide. This guideline should serve as a guide for clinicians and other experts caring for patients with CG. Though great effort was undertaken to formulate evidence-based recommendations, this was frequently hampered by limited evidence resulting in numerous recommendations based on expert opinion (18/40 recommendations, 45 %). The literature concerning CG available to date mostly consists of studies with an observational study-design. In the current era of evidence-based medicine these studies are labeled as having a low to very low level of evidence. Therefore strength of recommendation is 'discretionary' for a majority of recommendations in the guidelines, (32/40 recommendations, 80 %) including the recommendations labeled expert opinion. However, as other study designs (such as RCTs or cohort studies) are usually not feasible or may not provide the best design to study characteristics of rare diseases, the strength of the recommendation was upgraded to 'strong' when results were consistent across multiple studies, and experts had confidence in the validity of these results (9/40 recommendations, 23 %).

Future perspectives

Following this conclusion, it is not unexpected that gaps of knowledge were identified in most discussed fields of interest, foremost in the fields of treatment and follow-up. Topics of major importance for future research include: further assessment of which patients should be treated (cut-off enzyme activity), exploration for possible further relaxation of the diet for patients after childhood, exploration of new biomarkers for biochemical follow-up as well as reproductive function, assessment of executive functions in children and adults, and further exploration of bone turnover markers in relation to BMD.

Guideline update

Revision of this guideline is important as it only represents evidence in predefined areas up to October 2015. Since research in the field of CG is flourishing, it is expected that new information will be gained in the next decade. This guideline is scheduled to be updated in the next 10 years by representatives of the GalNet.

Acknowledgments The authors would like to thank Arnold G.E. Leenders for the time and effort invested in developing the search strategies for this guideline. We would also like to thank the representatives of the European Galactosemia Society, Mr. Jeroen van Kempen (chairman) and Mrs. Maaike van Kempen (commission member), for their contributions to this guideline.

Compliance with ethical standards

Conflict of interest Annet M. Bosch is in receipt of research grants from Nutricia and was a member of an advisory board for Nutricia.

Laurie E. Bernstein declares that she has no conflict of interest.

Gerard T. Berry declares that he has no conflict of interest.

Alberto B. Burlina declares that he has no conflict of interest.

Francois Eyskens declares that he has no conflict of interest.

Matthias Gautschi declares that he has no conflict of interest.

Stephanie Grünewald declares that she has no conflict of interest.

Cynthia S. Gubbels declares that she has no conflict of interest.

Ina Knerr declares that she has no conflict of interest.

Philippe Labrune declares that he has no conflict of interest.

Johanna H. van der Lee declares that she has no conflict of interest.

Anita MacDonald is in receipt of research grants from Nutricia and Vitaflo International, and is a member of the Nutricia IMD Advisory Board and Arla Advisory Board.

Elaine Murphy is in receipt of clinical trial funding from Vitaflo and received travel support to attend meetings from Vitaflo.

Katrin Õunap declares that she has no conflict of interest.

Pat A. Portnoi received grants from Nutricia and Vitaflo to attend conferences, meetings or to give lectures in the past 5 years.

Nancy L. Potter declares that she has no conflict of interest.

M. Estela Rubio-Gozalbo declares that she has no conflict of interest.

Jessica B. Spencer declares that she has no conflict of interest.

Inge Timmers declares that she has no conflict of interest.

Eileen P. Treacy declares that she has no conflict of interest.

Sandra C. Van Calcar declares that she has no conflict of interest.

Susan E. Waisbren declares that she has no conflict of interest.

Lindsey Welling declares that she has no conflict of interest.

Details of funding The initial GalNet meeting was financially supported by The Netherlands Organisation for Scientific Research (NWO; http://www.nwo.nl). The final consensus meeting was financially supported by the United States Galactosemia Foundation Inc. (patient organization; http://www.galactosemia.org/). The authors confirm independence from the sponsors; the content of the article has not been influenced by the sponsors.

Open Access This article is distributed under the terms of the Creative Commons Attribution 4.0 International License (http://creativecommons.org/licenses/by/4.0/), which permits unrestricted use, distribution, and reproduction in any medium, provided you give appropriate credit to the original author(s) and the source, provide a link to the Creative Commons license, and indicate if changes were made.

References

Adam S, Akroyd R, Bernabei S et al (2015) How strict is galactose restriction in adults with galactosaemia? International practice. Mol Genet Metab 115:23–6. doi:10.1016/j.ymgme.2015.03.008

Antshel KM, Epstein IO, Waisbren SE (2004) Cognitive strengths and weaknesses in children and adolescents homozygous for the galactosemia Q188R mutation: a descriptive study. Neuropsychology 18:658–664

Aydin-Özemir Z, Tekturk P, Uyguner ZO, Baykan B (2014) Galactosemia and phantom absence seizures. J Pediatr Neurosci 9:253–256

Badawi N, Cahalane SF, McDonald M et al (1996) Galactosaemia—a controversial disorder. Screening & outcome. Ireland 1972–1992. Ir Med J 89:16–17

Badik JR, Castaneda U, Gleason TJ et al (2011) Ovarian function in Duarte galactosemia. Fertil Steril 96:469–473

Batey LA, Welt CK, Rohr F et al (2013) Skeletal health in adult patients with classic galactosemia. Osteoporos Int 24:501–509

Beigi B, O'Keefe M, Bowell R et al (1993) Ophthalmic findings in classical galactosaemia—prospective study. Br J Ophthalmol 77:162–164

Belman AL, Moshe SL, Zimmerman RD (1986) Computed tomographic demonstration of cerebral edema in a child with galactosemia. Pediatrics 78:606–9

Bernstein LE, Van Calcar SC, Rohr F et al (2014) Galactosemia: the diet. Children's Hospital Colorado in collaboration with the Galactosemia Foundation Task Force

Berry GT, Palmieri M, Gross KC et al (1993) The effect of dietary fruits and vegetables on urinary galactitol excretion in galactose-1-phosphate uridyltransferase deficiency. J Inherit Metab Dis 16:91–100

Berry GT, Nissim I, Lin Z et al (1995) Endogenous synthesis of galactose in normal men and patients with hereditary galactosaemia. Lancet 346:1073–4

Berry GT, Nissim I, Gibson JB et al (1997) Quantitative assessment of whole body galactose metabolism in galactosemic patients. Eur J Pediatr 156(Suppl):S43–9

Berry GT, Singh RH, Mazur AT et al (2000) Galactose breath testing distinguishes variant and severe galactose-1-phosphate uridyltransferase genotypes. Pediatr Res 48:323–8. doi:10.1203/00006450-200009000-00010

Berry GT, Moate PJ, Reynolds RA et al (2004) The rate of de novo galactose synthesis in patients with galactose-1-phosphate uridyltransferase deficiency. Mol Genet Metab 81:22–30

Bhat M, Haase C, Lee PJ (2005) Social outcome in treated individuals with inherited metabolic disorders: UK study. J Inherit Metab Dis 28:825–830

Bosch AM (2006) Classical galactosaemia revisited. J Inherit Metab Dis 29:516–525

Bosch AM, Bakker HD, Wenniger-Prick LJ et al (2004a) High tolerance for oral galactose in classical galactosaemia: dietary implications. Arch Dis Child 89:1034–1036. doi:10.1136/adc.2003.037671

Bosch AM, Grootenhuis MA, Bakker HD et al (2004b) Living with classical galactosemia: health-related quality of life consequences. Pediatrics 113:e423–8

Bosch AM, Maurice-Stam H, Wijburg FA, Grootenhuis MA (2009) Remarkable differences: the course of life of young adults with galactosaemia and PKU. J Inherit Metab Dis 32:706–12. doi:10.1007/s10545-009-1253-2

Broer SL, Broekmans FJM, Laven JSE, Fauser BCJM (2014) Anti-Mullerian hormone: ovarian reserve testing and its potential clinical implications. Hum Reprod Update. doi:10.1093/humupd/dmu020

Burke JP, O'Keefe M, Bowell R, Naughten ER (1988) Cataracts in children with classical galactosaemia and in their parents. J Inherit Metab Dis 11(Suppl 2):246–8

Burke JP, O'Keefe M, Bowell R, Naughten ER (1989) Ophthalmic findings in classical galactosemia–a screened population. J Pediatr Ophthalmol Strabismus 26:165–168

Calderon FRO, Nelson L, Dobrowolski P et al (2007) Combination of enzyme analysis, allele-specific PCR and sequencing to detect mutations in the GALT gene. J Inherit Metab Dis 30: 818. doi:10.1007/s10545-007-0461-x

Carney AE, Sanders RD, Garza KR et al (2009) Origins, distribution and expression of the Duarte-2 (D2) allele of galactose-1-phosphate uridylyltransferase. Hum Mol Genet 18:1624–1632. doi:10.1093/hmg/ddp080

Cleary MA, Heptinstall LE, Wraith JE, Walter JH (1995) Galactosaemia: relationship of IQ to biochemical control and genotype. J Inherit Metab Dis 18:151–2

Coss KP, Byrne JC, Coman DJ et al (2012) IgG N-glycans as potential biomarkers for determining galactose tolerance in Classical Galactosaemia. Mol Genet Metab 105:212–220. doi:10.1016/j.ymgme.2011.10.018

Coss KP, Doran PP, Owoeye C et al (2013) Classical Galactosaemia in Ireland: incidence, complications and outcomes of treatment. J Inherit Metab Dis 36:21–7. doi:10.1007/s10545-012-9507-9

Coss KP, Hawkes CP, Adamczyk B et al (2014) N-glycan abnormalities in children with galactosemia. J Proteome Res 13:385–94. doi:10.1021/pr4008305

Crabtree NJ, Arabi A, Bachrach LK et al (2014) Dual-energy X-ray absorptiometry interpretation and reporting in children and adolescents: the revised 2013 ISCD Pediatric Official Positions. J Clin Densitom 17:225–42. doi:10.1016/j.jocd.2014.01.003

Donnell GN, Collado M, Koch R (1961) Growth and development of children with galactosemia. J Pediatr 58:845–851. doi:10.1016/S0022-3476(61)80140-6

Doulgeraki A, Monopolis I, Deligianni D et al (2014) Body composition in young patients with galactose metabolic disorders: a preliminary report. J Pediatr Endocrinol Metab 27:81–6. doi:10.1515/jpem-2013-0161

Doyle CM, Channon S, Orlowska D, Lee PJ (2010) The neuropsychological profile of galactosaemia. J Inherit Metab Dis 33:603–609. doi:10.1007/s10545-010-9154-y

Dubroff JG, Ficicioglu C, Segal S et al (2008) FDG-PET findings in patients with galactosaemia. J Inherit Metab Dis 31:533–539

El-Bassyouni HT, Ashour AM, Ezzat A et al (2006) The effect of diet on antioxidant status in patients with galactosemia. J Med Sci 6:452–457

Elsas LJ, Dembure PP, Langley S et al (1994) A common mutation associated with the Duarte galactosemia allele. Am J Hum Genet 54:1030–1036

Ficicioglu C, Yager C, Segal S (2005) Galactitol and galactonate in red blood cells of children with the Duarte/galactosemia genotype. Mol Genet Metab 84:152–9

Ficicioglu C, Thomas N, Yager C et al (2008) Duarte (DG) galactosemia: a pilot study of biochemical and neurodevelopmental assessment in children detected by newborn screening. Mol Genet Metab 95:206–212

Ficicioglu C, Hussa C, Gallagher PR et al (2010) Monitoring of biochemical status in children with Duarte galactosemia: utility of galactose, galactitol, galactonate, and galactose 1-phosphate. Clin Chem 56: 1177–82. doi:10.1373/clinchem.2010.144097

Fishler K, Koch R, Donnell G, Graliker BV (1966) Psychological correlates in galactosemia. Am J Ment Defic 71:116–125

Forges T, Monnier-Barbarino P, Leheup B, Jouvet P (2006) Pathophysiology of impaired ovarian function in galactosaemia. Hum Reprod Update 12:573–584

Fridovich-Keil JL, Walter JH (2008) Galactosaemia chapter 72. Valle D, Vogelstein B, Kinzler KW, Antonarakis SE, Ballabio A et al (eds) The Online Metabolic and Molecular Bases of Inherited Disease, OMMBID, Part 7: Carbohydrates. McGraw Hill, New York

Fridovich-Keil JL, Gubbels CS, Spencer JB et al (2011) Ovarian function in girls and women with GALT-deficiency galactosemia. J Inherit Metab Dis 34:357–366. doi:10.1007/s10545-010-9221-4

Fridovich-Keil JL, Gambello M, Singh R, Sharer J (2014) Duarte variant galactosemia. Pagon RA, Adam MP, Ardinger HH, Wallace SE, Amemiya A, Bean LJH, Bird TD, Fong CT, Mefford HC, Smith RJH, Stephens K (eds) GeneReviews® [Internet]. Seattle Univ. Washington, Seattle 1993–2015

Gajewska J, Ambroszkiewicz J, Radomyska B, Laskowska-Klita T (2006) Bone turnover markers in prepubertal children with classical galactosemia. Indian J Gastroenterol 25:221–2

Gajewska J, Ambroszkiewicz J, Radomyska B et al (2008) Serum markers of bone turnover in children and adolescents with classic galactosemia. Adv Med Sci 53:214–220. doi:10.2478/v10039-008-0026-8

Gubbels CS, Land JA, Rubio-Gozalbo ME (2008) Fertility and impact of pregnancies on the mother and child in classic galactosemia. Obstet Gynecol Surv 63:334–43. doi:10.1097/OGX.0b013e31816ff6c5

Gubbels CS, Maurice-Stam H, Berry GT et al (2011a) Psychosocial developmental milestones in men with classic galactosemia. J Inherit Metab Dis 34:415–419. doi:10.1007/s10545-011-9290-z

Gubbels CS, Thomas CMG, Wodzig WKWH et al (2011b) FSH isoform pattern in classic galactosemia. J Inherit Metab Dis 34:387–90. doi:10.1007/s10545-010-9180-9

Gubbels CS, Land JA, Evers JLH et al (2013a) Primary ovarian insufficiency in classic galactosemia: role of FSH dysfunction and timing of the lesion. J Inherit Metab Dis 36:29–34. doi:10.1007/s10545-012-9497-7

Gubbels CS, Welt CK, Dumoulin JC et al (2013b) The male reproductive system in classic galactosemia: cryptorchidism and low semen volume. J Inherit Metab Dis 36:779–786

Guerrero NV, Singh RH, Manatunga A et al (2000) Risk factors for premature ovarian failure in females with galactosemia. J Pediatr 137:833–41

Hansen TW, Henrichsen B, Rasmussen RK et al (1996) Neuropsychological and linguistic follow-up studies of children with galactosaemia from an unscreened population. Acta Paediatr 85:1197–1201

Hoffmann B, Wendel U, Schweitzer-Krantz S (2011) Cross-sectional analysis of speech and cognitive performance in 32 patients with classic galactosemia. J Inherit Metab Dis 34:421–427. doi:10.1007/s10545-011-9297-5

Hoffmann B, Dragano N, Schweitzer-Krantz S (2012) Living situation, occupation and health-related quality of life in adult patients with classic galactosemia. J Inherit Metab Dis 35:1051–1058. doi:10.1007/s10545-012-9469-y

Hughes J, Ryan S, Lambert D et al (2009) Outcomes of siblings with classical galactosemia. J Pediatr 154:721–726

Huidekoper HH, Bosch AM, Van Der Crabben SN et al (2005) Short-term exogenous galactose supplementation does not influence rate of appearance of galactose in patients with classical galactosemia. Mol Genet Metab 84:265–272. doi:10.1016/j.ymgme.2004.09.013

Hutchesson AC, Murdoch-Davis C, Green A et al (1999) Biochemical monitoring of treatment for galactosaemia: biological variability in metabolite concentrations. J Inherit Metab Dis 22:139–48

Huttenlocher PR, Hillman RE, Hsia YE (1970) Pseudotumor cerebri in galactosemia. J Pediatr 76:902–5

ISCD (2015) http://www.iscd.org/official-positions/6th-iscd-position-development-conference-adult/. In: Date last accessed 2015-11-29

Jones IE, Williams SM, Dow N, Goulding A (2002) How many children remain fracture-free during growth? A longitudinal study of children and adolescents participating in the Dunedin Multidisciplinary Health and Development Study. Osteoporos Int 13:990–5. doi:10.1007/s001980200137

Jumbo-Lucioni PP, Garber K, Kiel J et al (2012) Diversity of approaches to classic galactosemia around the world: a comparison of diagnosis, intervention, and outcomes. J Inherit Metab Dis 35:1037–49. doi:10.1007/s10545-012-9477-y

Karadag N, Zenciroglu A, Eminoglu FT et al (2013) Literature review and outcome of classic galactosemia diagnosed in the neonatal period. Clin Lab 59:1139–1146

Kaufman FR, Kogut MD, Donnell GN et al (1981) Hypergonadotropic hypogonadism in female patients with galactosemia. N Engl J Med 304:994–8. doi:10.1056/NEJM198104233041702

Kaufman FR, Xu YK, Ng WG, Donnell GN (1988) Correlation of ovarian function with galactose-1-phosphate uridyl transferase levels in galactosemia. J Pediatr 112:754–6

Kaufman FR, Loro ML, Azen C et al (1993) Effect of hypogonadism and deficient calcium intake on bone density in patients with galactosemia. J Pediatr 123:365–370

Kaufman FR, Reichardt JK, Ng WG et al (1994) Correlation of cognitive, neurologic, and ovarian outcome with the Q188R mutation of the galactose-1-phosphate uridyltransferase gene. J Pediatr 125:225–7

Kaufman FR, McBride-Chang C, Manis FR et al (1995) Cognitive functioning, neurologic status and brain imaging in classical galactosemia. Eur J Pediatr 154:S2–S5

Knerr I, Coss KP, Kratzsch J et al (2015) Effects of temporary low-dose galactose supplements in children aged 5–12 y with classical galactosemia: a pilot study. Pediatr Res 78:272–9. doi:10.1038/pr.2015.107

Ko D-H, Jun S-H, Park H-D et al (2010) Multiplex enzyme assay for galactosemia using ultraperformance liquid chromatography-tandem mass spectrometry. Clin Chem 56: 764–71. doi:10.1373/clinchem.2009.139618

Komrower GM, Lee DH (1970) Long-term follow-up of galactosaemia. Arch Dis Child 45:367–373

Kossowicz H, Zbieg Sendecka E (1977) Cataracts in galactosemia. Metab Ophthalmol 1:115–116

Kozak L, Francova H, Pijackova A et al (1999) Presence of a deletion in the 5′ upstream region of the GALT gene in Duarte (D2) alleles. J Med Genet 36:576–578

Krabbi K, Uudelepp M-L, Joost K et al (2011) Long-term complications in Estonian galactosemia patients with a less strict lactose-free diet and metabolic control. Mol Genet Metab 103:249–53. doi:10.1016/j.ymgme.2011.03.023

Lai K, Langley SD, Singh RH et al (1996) A prevalent mutation for galactosemia among black Americans. J Pediatr 128:89–95

Lambert C, Boneh A (2004) The impact of galactosaemia on quality of life—a pilot study. J Inherit Metab Dis 27:601–8

Lee DH (1972) Psychological aspects of galactosaemia. J Ment Defic Res 16:173–191

Lee PJ, Lilburn M, Wendel U, Schadewaldt P (2003) A woman with untreated galactosaemia. Lancet 362:446

Li Y, Ptolemy AS, Harmonay L et al (2010) Quantification of galactose-1-phosphate uridyltransferase enzyme activity by liquid chromatography-tandem mass spectrometry. Clin Chem 56:772–80. doi:10.1373/clinchem.2009.140459

Li Y, Ptolemy AS, Harmonay L et al (2011) Ultra fast and sensitive liquid chromatography tandem mass spectrometry based assay for galactose-1-phosphate uridylyltransferase and galactokinase deficiencies. Mol Genet Metab 102:33–40. doi:10.1016/j.ymgme.2010.08.018

Lindhout M, Rubio-Gozalbo ME, Bakker JA, Bierau J (2010) Direct non-radioactive assay of galactose-1-phosphate:uridyltransferase activity using high performance liquid chromatography. Clin Chim Acta 411:980–3. doi:10.1016/j.cca.2010.03.032

Liu G, Hale GE, Hughes CL (2000) Galactose metabolism and ovarian toxicity. Reprod Toxicol 14:377–384

Lynch ME, Potter NL, Coles CD, Fridovich-Keil JL (2015) Developmental outcomes of school-age children with duarte galactosemia: a pilot study. JIMD Rep 19:75–84. doi:10.1007/8904_2014_370

Manis FR, Cohn LB, McBride-Chang C et al (1997) A longitudinal study of cognitive functioning in patients with classical galactosaemia, including a cohort treated with oral uridine. J Inherit Metab Dis 20:549–555

Milánkovics I, Schuler A, Kámory E et al (2010) Molecular and clinical analysis of patients with classic and Duarte galactosemia in western Hungary. Wien Klin Wochenschr 122:95–102. doi:10.1007/s00508-010-1311-7

Moller HE, Ullrich K, Vermathen P et al (1995) In vivo study of brain metabolism in galactosemia by 1H and 31P magnetic resonance spectroscopy. Eur J Pediatr 154:S8–13

Najafi M, Khodadad A, Khatami GR (2005) Cerebral edema: a rare complication in galactosemia. Med J Islam Repub Iran 18:371–373

Nelson CD, Waggoner DD, Donnell GN et al (1991) Verbal dyspraxia in treated galactosemia. Pediatrics 88:346–350

Nelson MD, Wolff JA, Cross CA et al (1992) Galactosemia: evaluation with MR imaging. Radiology 184:255–61. doi:10.1148/radiology.184.1.1319076

Ning C, Fenn PT, Blair IA et al (2000) Apparent galactose appearance rate in human galactosemia based on plasma [(13)C]galactose isotopic enrichment. Mol Genet Metab 70: 261–271. doi:10.1006/mgme.2000.3026

Ning C, Reynolds R, Chen J et al (2001) Galactose metabolism in mice with galactose-1-phosphate uridyltransferase deficiency: sucklings and 7-week-old animals fed a high-galactose diet. Mol Genet Metab 72:306–315

O'Herlihy C, Danks D (1985) Endocrine evidence of prenatal follicular destruction in girls with galactosaemia. Pediatr Adolesc Gynecol 3:1–6

Ounap K, Joost K, Temberg T et al (2010) Classical galactosemia in Estonia: selective neonatal screening, incidence, and genotype/phenotype data of diagnosed patients. J Inherit Metab Dis 33:175–6. doi:10.1007/s10545-010-9045-2

Palmieri M, Mazur A, Berry GT et al (1999) Urine and plasma galactitol in patients with galactose-1-phosphate uridyltransferase deficiency galactosemia. Metabolism 48:1294–302

Panis B, Forget PP, Van Kroonenburgh MJPG et al (2004) Bone metabolism in galactosemia. Bone 35:982–987

Panis B, Bakker JA, Sels JPJE et al (2006a) Untreated classical galactosemia patient with mild phenotype. Mol Genet Metab 89:277–279. doi:10.1016/j.ymgme.2006.03.002

Panis B, Vermeer C, van Kroonenburgh MJPG et al (2006b) Effect of calcium, vitamins K1 and D3 on bone in galactosemia. Bone 39: 1123–9. doi:10.1016/j.bone.2006.05.002

Pesce MA, Bodourian SH (1982) Clinical significance of plasma galactose and erythrocyte galactose1phosphate measurements in

transferasedeficient galactosemia and in individuals with belownormal transferase activity. Clin Chem 28:301–305.
Portnoi PA, MacDonald A (2009) Determination of the lactose and galactose content of cheese for use in the galactosaemia diet. J Hum Nutr Diet 22:400–408
Portnoi PA, MacDonald A (2015) The lactose and galactose content of cheese suitable for galactosaemia: new analysis. JIMD Rep. doi:10.1007/8904_2015_520
Potter NL (2011) Voice disorders in children with classic galactosemia. J Inherit Metab Dis 34:377–385. doi:10.1007/s10545-010-9213-4
Potter NL, Lazarus JA, Johnson JM et al (2008) Correlates of language impairment in children with galactosaemia. J Inherit Metab Dis 31: 524–532. doi:10.1007/s10545-008-0877-y
Potter NL, Nievergelt Y, Shriberg LD (2013) Motor and speech disorders in classic galactosemia. JIMD Rep 11:31–41. doi:10.1007/8904_2013_219
Powell KK, Van Naarden Braun K, Singh RH et al (2009) Long-term speech and language developmental issues among children with Duarte galactosemia. Genet Med 11:874–879. doi:10.1097/GIM.0b013e3181c0c38d
Prestoz LL, Couto AS, Shin YS, Petry KG (1997) Altered follicle stimulating hormone isoforms in female galactosaemia patients. Eur J Pediatr 156:116–20
Rasmussen RK, Andreassen AB, Stromme P, Hansen TW (1996) Learning disabilities and language pathology in patients with galactosemia. Logoped Phoniatr Vocol 21:157–162. doi:10.3109/14015439609098884
Robertson A, Singh RH, Guerrero N V, et al (2000) Outcomes analysis of verbal dyspraxia in classic galactosemia. Genet Med 2:142–148
Rubio-Agusti I, Carecchio M, Bhatia KP et al (2013) Movement disorders in adult patients with classical galactosemia. Mov Disord 28: 804–10. doi:10.1002/mds.25348
Rubio-Gozalbo ME, Hamming S, van Kroonenburgh MJ et al (2002) Bone mineral density in patients with classic galactosaemia. Arch Dis Child 87:57–60
Rubio-Gozalbo ME, Panis B, Zimmermann LJ et al (2006) The endocrine system in treated patients with classical galactosemia. Mol Genet Metab 89:316–322
Rubio-Gozalbo ME, Gubbels CS, Bakker JA et al (2010) Gonadal function in male and female patients with classic galactosemia. Hum Reprod Update 16:177–188
Rutherford PJ, Davidson DC, Matthai SM (2002) Dietary calcium in galactosaemia. J Hum Nutr Diet 15:39–42
Sanders RD, Spencer JB, Epstein MP et al (2009) Biomarkers of ovarian function in girls and women with classic galactosemia. Fertil Steril 92:344–51. doi:10.1016/j.fertnstert.2008.04.060
Sauer MV, Kaufman FR, Paulson RJ, Lobo RA (1991) Pregnancy after oocyte donation to a woman with ovarian failure and classical galactosemia. Fertil Steril 55:1197–1199
Schadewaldt P, Kamalanathan L, Hammen HW, Wendel U (2004) Age dependence of endogenous galactose formation in Q188R homozygous galactosemic patients. Mol Genet Metab 81:31–44. doi:10.1016/j.ymgme.2003.10.007
Schadewaldt P, Hoffmann B, Hammen HW et al (2010) Longitudinal assessment of intellectual achievement in patients with classical galactosemia. Pediatrics 125:e374–81. doi:10.1542/peds.2008-3325
Schadewaldt P, Kamalanathan L, Hammen HW et al (2014) Endogenous galactose formation in galactose-1-phosphate uridyltransferase. Arch Physiol Biochem 120:228–39
Schwarz HP, Schaefer T, Bachmann C (1985) Galactose and galactitol in the urine of children with compound heterozygosity for Duarte variant and classical galactosemia (GtD/gt) after an oral galactose load. Clin Chem 31:420–2
Schweitzer S, Shin Y, Jakobs C, Brodehl J (1993) Long-term outcome in 134 patients with galactosaemia. Eur J Pediatr 152:36–43
Shield JP, Wadsworth EJ, MacDonald A et al (2000) The relationship of genotype to cognitive outcome in galactosaemia. Arch Dis Child 83:248–50
Shriberg LD, Potter NL, Strand EA (2011) Prevalence and phenotype of childhood apraxia of speech in youth with galactosemia. J Speech Lang Hear Res 54:487–519. doi:10.1044/1092-4388(2010/10-0068)
Spencer JB, Badik JR, Ryan EL et al (2013) Modifiers of ovarian function in girls and women with classic galactosemia. J Clin Endocrinol Metab 98:E1257–65. doi:10.1210/jc.2013-1374
ten Hoedt AE, Maurice-Stam H, Boelen CCA et al (2011) Parenting a child with phenylketonuria or galactosemia: implications for health-related quality of life. J Inherit Metab Dis 34:391–8. doi:10.1007/s10545-010-9267-3
The GRADE Working Group (2009) GRADE handbook for grading quality of evidence and strength of recommendation. Version 3.2 [updated March 2009]
Timmers I, Jansma BM, Rubio-Gozalbo ME (2012) From mind to mouth: event related potentials of sentence production in classic galactosemia. PLoS ONE 7:e52826. doi:10.1371/journal.pone.0052826
Timmers I, van den Hurk J, Hofman PA et al (2015a) Affected functional networks associated with sentence production in classic galactosemia. Brain Res 1616:166–176. doi:10.1016/j.brainres.2015.05.007
Timmers I, Zhang H, Bastiani M et al (2015b) White matter microstructure pathology in classic galactosemia revealed by neurite orientation dispersion and density imaging. J Inherit Metab Dis 38:295–304. doi:10.1007/s10545-014-9780-x
Van Calcar SC, Bernstein LE, Rohr FJ et al (2014) A reevaluation of life-long severe galactose restriction for the nutrition management of classic galactosemia. Mol Genet Metab 112:191–197. doi:10.1016/j.ymgme.2014.04.004
Van Erven B, Gubbels CS, van Golde RJ et al (2013) Fertility preservation in female classic galactosemia patients. Orphanet J Rare Dis 8: 107
Vandenplas Y, Castrellon PG, Rivas R et al (2014) Safety of soya-based infant formulas in children. Br J Nutr 111:1340–60. doi:10.1017/S0007114513003942
Varela-Lema L, Paz-Valinas L, Atienza-Merino G et al (2016) Appropriateness of newborn screening for classic galactosaemia: a systematic review. J Inherit Metab Dis 1–17. doi:10.1007/s10545-016-9936-y
Viggiano E, Marabotti A, Burlina AP et al (2015) Clinical and molecular spectra in galactosemic patients from neonatal screening in northeastern Italy: structural and functional characterization of new variations in the galactose-1-phosphate uridyltransferase (GALT) gene. Gene 559:112–8. doi:10.1016/j.gene.2015.01.013
Waggoner DD, Buist NR, Donnell GN (1990) Long-term prognosis in galactosaemia: results of a survey of 350 cases. J Inherit Metab Dis 13:802–818
Waisbren SE, Norman TR, Schnell RR, Levy HL (1983) Speech and language deficits in early-treated children with galactosemia. J Pediatr 102:75–77
Waisbren SE, Potter NL, Gordon CM et al (2012) The adult galactosemic phenotype. J Inherit Metab Dis 35:279–286. doi:10.1007/s10545-011-9372-y
Walter JH, Collins JE, Leonard JV (1999) Recommendations for the management of galactosaemia. UK Galactosaemia Steering Group. Arch Dis Child 80:93–6
Wang BB, Xu YK, Ng WG, Wong LJ (1998) Molecular and biochemical basis of galactosemia. Mol Genet Metab 63: 263–9. doi:10.1006/mgme.1998.2678
Wang ZJ, Berry GT, Dreha SF et al (2001) Proton magnetic resonance spectroscopy of brain metabolites in galactosemia. Ann Neurol 50:266–269
Webb AL, Singh RH, Kennedy MJ, Elsas LJ (2003) Verbal dyspraxia and galactosemia. Pediatr Res 53:396–402

Wehrli SL, Reynolds R, Chen J et al (2002) Metabolism of 13C galactose by lymphoblasts from patients with galactosemia determined by NMR spectroscopy. Mol Genet Metab 77:296–303

Widger J, O'Toole J, Geoghegan O et al (2010) Diet and visually significant cataracts in galactosaemia: is regular follow up necessary? J Inherit Metab Dis 33:129–132. doi:10.1007/s10545-009-9042-5

Widhalm K, Miranda-Da-Cruz B, De Sonneville LMJ (2002) Information processing characteristics and uridine treatment in children with classical galactosemia. Nutr Res 22:257–270. doi:10.1016/S0271-5317%2801%2900401-8

Wiesmann UN, Rosé-Beutler B, Schlüchter R (1995) Leguminosae in the diet: the raffinose-stachyose question. Eur J Pediatr 154:93–96. doi:10.1007/BF02143812

Xu YK, Kaufman FR, Donnell GN, Ng WG (1995) Radiochemical assay of minute quantities of galactose-1-phosphate uridyltransferase activity in erythrocytes and leukocytes of galactosemia patients. Clin Chim Acta 235:125–36

Yager C, Gibson J, States B et al (2001) Oxidation of galactose by galactose-1-phosphate uridyltransferase-deficient lymphoblasts. J Inherit Metab Dis 24:465–76

Yager CT, Chen J, Reynolds R, Segal S (2003) Galactitol and galactonate in red blood cells of galactosemic patients. Mol Genet Metab 80:283–9. doi:10.1016/j.ymgme.2003.08.021
