# Supplementary material for: International clinical guideline for the management of classical galactosemia: diagnosis, treatment, and follow-up
Source: J Inherit Metab Dis. 2016 Nov 17;40(2):171–6. doi: 10.1007/s10545-016-9990-5 (PMC5306419; doi:10.1007/s10545-016-9990-5)
Supplement: Supplementary file 2 — (PDF 441 kb) [file 10545_2016_9990_MOESM2_ESM.pdf]

Search strategies

Diagnostics

EMBASE January 2015
1 s135l.tw. or (duarte and (galactosem* or galt or g-1-p or utp*)).mp.

MEDLINE January 2015
1 s135l.tw. or (duarte and (galactosem* or galt or g-1-p or utp*)).mp.

Biochemical follow-up

EMBASE March 2015

1	galactosemia/
2	(galactosem* or ((galt or utp hexose 1 phosphate uridyltransferase or gale or galk or galactokinase) adj3 deficien*)).tw.
3	1 or 2
4	Reference Values/ or exp monitoring/ or (monitoring or biochemical).mp,kw.
5	Reference Standards/
6	chromatography/ or exp chromatography, gas/ or exp chromatography, liquid/
7	((reference adj3 (value* or standard*)) or chromatograph* or gc-ms or gcms).tw,kf.
8	or/4-7
9	Sugar Acids/
10	Galactosephosphates/
11	exp Galactitol/
12	exp Galactose/
13	(g-1-p or galactose-1-phosphat* or galatonic acid* or galactose or galactonate or galactitol).tw,kf.
14	or/9-13
15	3 and 8 and 14
16	3 and 14
17	exp longitudinal study/ or prospective study/ or retrospective study/ or exp prognosis/
18	(follow up or prospective or retrospective or longitudinal or case control or cohort or long term).tw,kf.
19	17 or 18
20	16 and 19
21	15 or 20

Diet

EMBASE October 2014

1	galactosemia/
2	(galactosem* or ((galt or utp hexose 1 phosphate uridyltransferase or gale or galk or galactokinase) adj3 deficien*)).tw.
3	1 or 2
4	Breast Feeding/ or exp diet therapy/ or artificial milk/ or calcium/ or Dietetics/ or Vegetable/ or exp Fruit/ or cheese/ or Soybean Milk/ or Casein/ or exp Vitamin/ or exp diet restriction/ or Milk/
5	(Breast Feeding or ((Infant or feeding) adj3 formula) or soybean* or calcium or Dietetic* or Vegetabl* or legume* or Fruit* or Milk or Casein* or cheese or Vitamin* or ((galactose or diet*) adj3 restriction)).tw,kw.
6	((Dietary or diet) adj3 (deficien* or compliance or treatment)).tw,kw.
7	or/4-6
8	3 and 7

MEDLINE October 2014

1	galactosemias/
2	(galactosem* or ((galt or utp hexose 1 phosphate uridyltransferase or gale or galk or galactokinase) adj3 deficien*)).tw.
3	1 or 2
4	Breast Feeding/ or dh.fs. or exp diet therapy/ or Infant Formula/ or calcium/ or Dietetics/ or Vegetables/ or exp Fruit/ or cheese/ or Soy Milk/ or exp Milk Substitutes/ or Caseins/ or exp Vitamins/ or (exp Diet/ and deficien*.mp,kf.) or Milk/
5	(Breast Feeding or ((Infant or feeding) adj3 formula) or calcium or Dietetic* or Vegetabl* or legume* or Fruit* or soybean* or Milk or Casein* or cheese or Vitamin* or ((galactose or diet*) adj3 restriction)).tw,kf.
6	((Dietary or diet) adj3 (deficien* or compliance or treatment)).tw,kf.
7	or/4-6
8	3 and 7

MEDLINE March 2015

1	Reference Values/ or exp Monitoring,Physiologic/ or (monitoring or biochemical).mp,kf.
2	Reference Standards/
3	chromatography/ or exp chromatography, gas/ or exp chromatography, liquid/
4	((reference adj3 (value* or standard*)) or chromatograph* or gc-ms or gcms).tw,kf.
5	or/1-4
6	Sugar Acids/
7	Galactosephosphates/
8	exp Galactitol/
9	exp Galactose/
10	(g-1-p or galactose-1-phosphat* or galatonic acid* or galactose or galactonate or galactitol).tw,kf.
11	or/6-10
12	galactosemias/
13	(galactosem* or ((galt utp hexose 1 phosphate uridyltransferase or gale or galk or galactokinase) adj3 deficien*)).tw.
14	12 or 13
15	5 and 11 and 14
16	14 and 11
17	exp epidemiologic studies/ or exp prognosis/
18	(follow up or prospective or retrospective or longitudinal or case control or cohort or long term).tw,kf.
19	17 or 18
20	16 and 19
21	15 or 20

<u>Developmental follow-up/Speech and Language</u>

MEDLINE June 2014

1. galactosemias/

2. (galactosem* or ((galt utp hexose 1 phosphate uridyltransferase or gale or galk or galactokinase) adj3 deficien*)).tw.

3. 1 or 2

4. exp psychologic test/

5. ((intelligence or psychological or language or neuropsychological) adj3 test*).tw,kw.

6. speech therapy/

7. ((language or speech or logoped*) adj3 (therap* or educat* or train*)).tw,kw.

8. exp hearing disorder/

9. (((auditor* or hearing) adj3 (disorder* or problem*)) or apraxia or dyspraxia).tw,kw.

10. executive function/ or (executive adj3 function*).tw,kf.

11. exp intelligence/

12. exp memory disorder/

13. (iq or intelligence quotient or amnesia or memory or problem solving).tw,kw.

14. exp *galactosemia/co

15. (long term adj5 (outcom* or therap* or treatment*)).tw,kw.

16. clinical study/ or case control study/

17. exp language disorders/

18. exp "rehabilitation of speech and language disorders"/

19. exp Cognition Disorders/

20. exp Mental Disorders/et [Etiology]

21. Developmental Disabilities/et [Etiology]

22. exp epidemiologic studies/ or (prospective or retrospective or cohort or case control*).tw,kf.

23. or/4-22

24. 3 and 23

EMBASE June 2014

1. galactosemia/

2. (galactosem* or ((galt utp hexose 1 phosphate uridyltransferase or gale or galk or galactokinase) adj3 deficien*)).tw.

3. 1 or 2

4. exp psychologic test/

5. exp psychometry/

6. ((intelligence or psychological or language or neuropsychological) adj3 test*).tw,kw.

7. exp language disability/

8. speech therapy/

9. ((language or speech or logoped*) adj3 (therap* or educat* or train*)).tw,kw.

10. exp hearing disorder/

11. (((auditor* or hearing) adj3 (disorder* or problem*)) or apraxia or dyspraxia).tw,kw.

12. cognitive defect/ or (cognitive adj3 (defect* or disorder*)).tw,kw.

13. executive function/ or (executive adj3 function*).tw,kw.

14. exp intelligence/

15. exp memory disorder/

16. (iq or intelligence quotient or amnesia or memory or problem solving).tw,kw.

17. exp *galactosemia/co

18. exp mental development/

19. developmental disorder/et [Etiology]

20. (long term adj5 (outcom* or therap* or treatment*)).tw,kw.

21. exp case control study/ or exp longitudinal study/ or prospective study/ or retrospective study/ or (prospective or retrospective).tw.

22. or/4-21

23. 3 and 22

PSYCINFO June 2014

1. (galactosem* or ((galt utp hexose 1 phosphate uridyltransferase or gale or galk or galactokinase) adj3 deficien*)).tw.

Neurology

EMBASE December 2014

1	(uridylyltransferase or uridylyl transferase).mp.
2	galactosemia.mp. or galactosemias.tw.
3	1 or 2
4	exp nuclear magnetic resonance imaging/
5	exp tomography/
6	exp neurological examination/
7	exp evoked response/
8	(mri or tomograph* or (neurological adj3 examinat*) or magnetic resonance or fdg-pet or positron emission or (evoked adj3 (potential* or respons* or discharg*)) or electroencephalograph*).mp,kw.
9	or/4-8
10	3 and 9
11	(neurologic adj3 (sequel* or complication*)).tw,kw.
12	exp Ataxia/
13	exp motor dysfunction/
14	exp Epilepsy/
15	exp dyskinesia/
16	exp Psychomotor Disorders/
17	(atax* or seizure* or tremor* or epileps* or psychomoter* or ((motor or movement) adj3 (dysfunction* or disorder* or impairment* or disturban*)) or dyskines* or diskines*).mp,kw.
18	or/11-17
19	3 and 9 and 18
20	3 and 18
21	10 or 20

MEDLINE December 2014

1	galactosemias/
2	(galactosem* or ((uridyltransferase or gale or galk or galactokinase) adj3 deficien*)).tw,kf.
3	1 or 2
4	exp Magnetic Resonance Imaging/
5	exp Tomography, Emission-Computed/
6	exp Electroencephalography/
7	exp Evoked Potentials/
8	(mri or tomograph* or (neurological adj3 examinat*) or magnetic resonance or fdg-pet or positron emission or (evoked adj3 (potential* or respons* or discharg*)) or electroencephalograph*).mp,kf.
9	or/4-8
10	3 and 9
11	(neurologic adj3 (sequel* or complication*)).tw,kf.
12	exp Ataxia/
13	exp Dyskinesias/
14	exp Epilepsy/
15	Movement Disorders/
16	exp Psychomotor Disorders/
17	(atax* or seizure* or tremor* or epileps* or psychomoter* or ((motor or movement) adj3 (dysfunction* or disorder* or impairment* or disturban*)) or dyskines* or diskines*).mp,kf.
18	or/11-17
19	3 and 9 and 18
20	3 and 18
21	10 or 20

Psychinfo February 2015

1	(galactosem* or galt or uridyltransferase uridyl transferase or gale or galk or galactokinase).mp,id.
2	(mri or magnetic resonance or fdg-pet or positron emission or evoked potential* or electroencephalograph*).mp,kf.
3	exp neuroimaging/ or exp computer assisted diagnosis/ or exp evoked potentials/
4	2 or 3
5	1 and 4

Psychosocial development/Mental health

MEDLINE February 2015

1	exp "behavior and behavior mechanisms"/
2	galactosemias/
3	(galactosem* or ((galt utp hexose 1 phosphate uridyltransferase or gale or galk or galactokinase) adj3 deficien*)).tw.
4	2 or 3
5	1 and 4
6	exp Socioeconomic Factors/
7	4 and 6
8	"Quality of Life"/
9	4 and 8
10	5 or 7 or 9
11	(psychosocial or psycho social or education* or occupation* or emotion* or quality of life or hrqol or coping or employment or schooling or friendship* or communicaion* or self-esteem or body image or anxiety or social adaptation or social behavio?r or iq or intelligence).mp,kf.
12	4 and 11
13	10 or 12
14	exp Neuropsychological Tests/
15	((neuropsychol* or linguistic or language) adj3 test*).tw,kf.
16	psychology.fs.
17	14 or 15 or 16
18	4 and 17
19	13 or 18

EMBASE February 2015

1	exp behavior/
2	galactosemia/
3	(galactosem* or ((galt or utp hexose 1 phosphate uridyltransferase or gale or galk or galactokinase) adj3 deficien*)).tw.
4	2 or 3
5	1 and 4
6	exp economic aspect/ or exp education/ or exp human activities/ or exp "occupation and occupation related phenomena"/ or exp "social aspects and related phenomena"/ or exp social belief/ or exp social change/ or exp socialization/ or exp welfare/
7	4 and 6
8	"Quality of Life"/
9	4 and 8
10	5 or 7 or 9
11	(psychosocial or psycho social or education* or occupation* or emotion* or quality of life or hrqol or coping or employment or schooling or friendship* or communicaion* or self-esteem or body image or anxiety or social adaptation or social behavio?r or iq or intelligence).mp,kw.
12	4 and 11
13	10 or 12
14	exp Neuropsychological Tests/
15	((neuropsychol* or linguistic or language) adj3 test*).tw,kw.
16	14 or 15
17	4 and 16
18	13 or 17

<u>Endocrinology/Fertility follow-up</u>

PUBMED March 2014

(("Endocrine System/pathology"[Mesh] OR "Endocrine System/physiology"[Mesh] OR "Endocrine System/physiopathology"[Mesh] OR "Endocrine System Diseases"[Mesh] OR ("primary ovarian insufficiency"[MeSH Terms] OR ("primary"[All Fields] AND "ovarian"[All Fields] AND "insufficiency"[All Fields]) OR "primary ovarian insufficiency"[All Fields]) OR premature ovarian failure[tiab] OR ("ovarian follicle"[MeSH Terms] OR ("ovarian"[All Fields] AND "follicle"[All Fields]) OR "ovarian follicle"[All Fields]) OR ("ovary"[MeSH Terms] OR "ovary"[All Fields]) OR ovaria[tiab] OR ovarium[tiab]) OR (("Ultrasonography"[Mesh] OR "ultrasonography"[Subheading] OR ultrasound[tiab] OR ultrasonic[tiab] OR (echograph[tiab] OR echographe[tiab] OR echographer[tiab] OR echographers[tiab] OR echographi[tiab] OR echographia[tiab] OR echographic[tiab] OR echographical[tiab] OR echographically[tiab] OR echographics[tiab] OR echographie[tiab] OR echographie'[tiab] OR echographied[tiab] OR echographies[tiab] OR echographique[tiab] OR echographist[tiab] OR echographist's[tiab] OR echographists[tiab] OR echographs[tiab] OR echography[tiab] OR echography's[tiab] OR echographyallow[tiab] OR echographyc[tiab] OR echographycally[tiab])) OR ("Magnetic Resonance Imaging"[Mesh] OR mri[tiab] OR (tomograph[tiab] OR tomograph's[tiab] OR tomographaphy[tiab] OR tomographay[tiab] OR tomographe[tiab] OR tomographed[tiab] OR tomographer[tiab] OR tomographers[tiab] OR tomographers'[tiab] OR tomographgy[tiab] OR tomographic[tiab] OR tomographic'[tiab] OR tomographical[tiab] OR tomographicaliy[tiab] OR tomographically[tiab] OR tomographicangiography[tiab] OR tomographicic[tiab] OR tomographiclike[tiab] OR tomographicpet[tiab] OR tomographics[tiab] OR tomographicscan[tiab] OR tomographie[tiab] OR tomographied[tiab] OR tomographies[tiab] OR tomographing[tiab] OR tomographio[tiab] OR tomographique[tiab] OR tomographis[tiab] OR tomographpy[tiab] OR tomographry[tiab] OR tomographs[tiab] OR tomographs'[tiab] OR tomographv[tiab] OR tomography[tiab] OR tomography'[tiab] OR tomography's[tiab] OR tomography8[tiab] OR tomographyangiography[tiab] OR tomographyare[tiab] OR tomographyc[tiab] OR tomographycally[tiab] OR tomographycomputed[tiab] OR tomographydiffraction[tiab] OR tomographyfor[tiab] OR tomographyi[tiab] OR tomographyic[tiab] OR tomographylike[tiab] OR tomographymra[tiab] OR tomographymrimagnetic[tiab] OR tomographynumber[tiab] OR tomographyosteoabsorptiometry[tiab] OR tomographyrevealed[tiab] OR tomographys[tiab] OR tomographyscan[tiab] OR tomographyscanners[tiab] OR tomographyshowed[tiab] OR tomographywas[tiab]) OR nmr[tiab] OR magnetic resonance[tiab])) OR ("inhibin B"[Supplementary Concept] OR inhibin b[tiab]) OR ("Luteinizing Hormone"[Mesh] OR lh[tiab] OR lutropin[tiab] OR (luteinizing hormone[tiab] OR luteinizing hormones[tiab]) OR interstitial cell stimulating[tiab] OR luteozyman[tiab] OR luteoziman[tiab]) OR ("Follicle Stimulating Hormone"[Mesh] OR fsh[tiab] OR follicle stimulating[tiab] OR follitropin[tiab]) OR ("Anti-Mullerian Hormone"[Mesh] OR "anti-Mullerian hormone receptor"[Supplementary Concept] OR anti mullerian[tiab] OR mullerian inhibiting[tiab] OR AMH[tiab]) OR ("Estrogens"[Mesh] OR "Estrogens"[Pharmacological Action] OR (estrogen[tiab] OR estrogen'[tiab] OR estrogen''[tiab] OR estrogen's[tiab] OR estrogen0[tiab] OR estrogen17beta[tiab] OR estrogenactive[tiab] OR estrogenaemia[tiab] OR estrogenated[tiab] OR estrogenation[tiab] OR estrogenbinding[tiab] OR estrogenci[tiab] OR estrogencontaining[tiab] OR estrogendepleted[tiab] OR estrogene[tiab] OR estrogenecity[tiab] OR estrogenemia[tiab] OR estrogenemic[tiab] OR estrogenes[tiab] OR estrogenesis[tiab] OR estrogenesity[tiab] OR

estrogenexcretion[tiab] OR estrogeni[tiab] OR estrogenia[tiab] OR estrogenias[tiab] OR estrogenic[tiab] OR estrogenic'[tiab] OR estrogenicactivitydatabaseeadb[tiab] OR estrogenical[tiab] OR estrogenically[tiab] OR estrogenicfusarium[tiab] OR estrogenicities[tiab] OR estrogenicity[tiab] OR estrogeniclike[tiab] OR estrogenico[tiab] OR estrogenicos[tiab] OR estrogenics[tiab] OR estrogenicty[tiab] OR estrogenicy[tiab] OR estrogeniety[tiab] OR estrogenindependent[tiab] OR estrogeninduced[tiab] OR estrogenisation[tiab] OR estrogenised[tiab] OR estrogenism[tiab] OR estrogenity[tiab] OR estrogenizating[tiab] OR estrogenization[tiab] OR estrogenization'[tiab] OR estrogenize[tiab] OR estrogenized[tiab] OR estrogenizing[tiab] OR estrogenlike[tiab] OR estrogenlutein[tiab] OR estrogenmimetic[tiab] OR estrogenmonotherapy[tiab] OR estrogennic[tiab] OR estrogeno[tiab] OR estrogenomimetic[tiab] OR estrogenopathies[tiab] OR estrogenophilic[tiab] OR estrogenoprogestational[tiab] OR estrogenotherapy[tiab] OR estrogenous[tiab] OR estrogenpositive[tiab] OR estrogenprimed[tiab] OR estrogenproducing[tiab] OR estrogenprogesterone[tiab] OR estrogenprogestogen[tiab] OR estrogenquinones[tiab] OR estrogenreceptor[tiab] OR estrogenreceptors[tiab] OR estrogens[tiab] OR estrogens'[tiab] OR estrogensim[tiab] OR estrogensulfatase[tiab] OR estrogentherapy[tiab] OR estrogentranquilizer[tiab] OR estrogentreated[tiab] OR estrogentreatment[tiab] OR estrogenuria[tiab] OR estrogenuric[tiab] OR estrogenvs[tiab])) OR (oestrogen[tiab] OR oestrogen'[tiab] OR oestrogen''cervical[tiab] OR oestrogen's[tiab] OR oestrogenaemia[tiab] OR oestrogenaemic[tiab] OR oestrogenand[tiab] OR oestrogencontaining[tiab] OR oestrogendependent[tiab] OR oestrogene[tiab] OR oestrogenecity[tiab] OR oestrogenemia[tiab] OR oestrogenenic[tiab] OR oestrogenes[tiab] OR oestrogeni[tiab] OR oestrogenia[tiab] OR oestrogenial[tiab] OR oestrogenic[tiab] OR oestrogenic'[tiab] OR oestrogenically[tiab] OR oestrogenicities[tiab] OR oestrogenicity[tiab] OR oestrogenine[tiab] OR oestrogenique[tiab] OR oestrogenisation[tiab] OR oestrogenised[tiab] OR oestrogenising[tiab] OR oestrogenism[tiab] OR oestrogenization[tiab] OR oestrogenized[tiab] OR oestrogenlike[tiab] OR oestrogennegative[tiab] OR oestrogeno[tiab] OR oestrogenotherapy[tiab] OR oestrogenprecursor[tiab] OR oestrogenprogestagen[tiab] OR oestrogenprogestogen[tiab] OR oestrogenreceptor[tiab] OR oestrogenreceptors[tiab] OR oestrogens[tiab] OR oestrogens'[tiab] OR oestrogensensitive[tiab] OR oestrogentherapy[tiab] OR oestrogenuria[tiab]) OR ("Progesterone"[Mesh] OR (progesteron[tiab] OR progesterona[tiab] OR progesteronaemia[tiab] OR progesteronal[tiab] OR progesteronbiguazon[tiab] OR progesterone[tiab] OR progesterone'[tiab] OR progesterone's[tiab] OR progesteronecaproate[tiab] OR progesteronee[tiab] OR progesteronegenesis[tiab] OR progesteroneinduced[tiab] OR progesteroneiud[tiab] OR progesteronelike[tiab] OR progesteronematabolism[tiab] OR progesteronemia[tiab] OR progesteronemy[tiab] OR progesteronen[tiab] OR progesteronenor[tiab] OR progesteronep[tiab] OR progesteroneprimed[tiab] OR progesteroneprofile[tiab] OR progesteronereceptor[tiab] OR progesteronereceptors[tiab] OR progesteronereceptortiters[tiab] OR progesterones[tiab] OR progesteronic[tiab] OR progesteronized[tiab] OR progesteronne[tiab] OR progesterono[tiab] OR progesteronr[tiab] OR progesterons[tiab] OR progesteronspiegel[tiab]) OR pregnenedione[tiab]) OR ("Hormone Replacement Therapy"[Mesh] OR hormone replacement[tiab] OR hormone substitution[tiab])) AND (("galactosemias"[MeSH Terms] OR "galactosemias"[All Fields]) OR (galactosemia[tiab] OR galactosemia's[tiab] OR galactosemias[tiab] OR galactosemic[tiab] OR galactosemic'gal7[tiab] OR galactosemics[tiab] OR galactosemis[tiab]) OR galt deficiency[tiab] OR (("utp-hexose-1-phosphate uridylyltransferase"[MeSH Terms] OR "udpglucose-hexose-1-phosphate

uridylyltransferase"[MeSH Terms]) AND ("deficiency"[Subheading] OR "deficiency"[All Fields])) OR (hypergonadotrophic[tiab] AND ("hypogonadism"[MeSH Terms] OR "hypogonadism"[All Fields])))

EMBASE March 2014

1. galactosemia/
2. (galactosem* or (galt adj3 deficienc*) or galtdeficienc* or (utp hexose 1 phosphate uridyltransferease adj3 deficien*) or (hypergonadotroph* adj3 hypogonadism)).tw.
3. 1 or 2
4. exp endocrine system/
5. endocrine disease/
6. exp premature ovarian failure/
7. ((primary or premature) adj2 ovarian adj2 (insufficiency or failure)).tw.
8. exp ovary/
9. (ovary or ovarium or ovaria).tw.
10. or/4-9
11. exp nuclear magnetic resonance imaging/
12. (magenetic resonance or mri or nmr or tomograph*).tw.
13. exp echography/
14. (ultrasound or echograph* or ultrasonic).tw.
15. or/11-14
16. exp inhibin B/
17. luteinizing hormone/
18. follitropin/
19. Muellerian inhibiting factor/
20. exp estrogen/
21. progesterone/
22. exp hormone substitution/
23. ((inhibin adj2 b) or lh or luteinizing hormone or lutropin or lutotropin or luteoziman or fsh or follicle stimulating or follitropin or anti mullerian or mullerian inhibiting or amh or estrogen* or oestrogen* or progesteron* or pregnenedion* or hormone replacement or hormone substitution).tw.
24. or/16-23
25. 10 or 15 or 24
26. 3 and 25

Bone health

EMBASE March 2015

1	galactosemias/
2	(galactosem* or ((galt utp hexose 1 phosphate uridyltransferase or gale or galk or galactokinase) adj3 deficien*)).tw.
3	1 or 2
4	Bone Density/
5	Bone Diseases, Metabolic/
6	exp bone metabolism/
7	exp "Bone and Bones"/
8	Calcium/ec or vitamin d/ec or vitamin k/ec
9	Absorptiometry, Photon/
10	Osteocalcin/
11	((bone adj3 density) or fracture or fractures or bone mass or bone resorption or bone metabolism or bone loss or bone mineral* or osteoporosis or osteolysis or skeletal health or bone turn over or dxa or dual energy x ray).mp,kw.
12	exp Fractures, Bone/
13	calcium blood level/
14	or/4-13
15	3 and 14

MEDLINE 01-03-2015

1	galactosemias/
2	(galactosem* or ((galt utp hexose 1 phosphate uridyltransferase or gale or galk or galactokinase) adj3 deficien*)).tw.
3	1 or 2
4	Bone Density/
5	Bone Diseases, Metabolic/
6	exp bone resorption/
7	exp "Bone and Bones"/
8	(Calcium/ or vitamin d/ or vitamin k/) and (me or bl).fs.
9	Absorptiometry, Photon/
10	Osteocalcin/
11	((bone adj3 density) or bone mass or fractures or fracture or bone metabolism or bone resorption or osteolysis or bone loss or bone mineral* or osteoporosis or osteolysis or skeletal health or bone turn over or dxa or dual energy x ray).mp,kf.
12	exp Fractures, Bone/
13	or/4-12
14	3 and 13

Ophthalmological complications

EMBASE April 2015

1 galactosemia/
2 (galactosem* or ((galt or utp hexose 1 phosphate uridyltransferase or gale or galk or galactokinase) adj3 deficien*)).tw.
3 1 or 2
4 Cataract/
5 Vitreous Hemorrhage/
6 exp Lens/
7 exp Vision Test/
8 (vision or ophthalm* or crystalline or cataract* or ocular or vitreous).tw,kw.
9 or/4-8
10 3 and 9

MEDLINE April 2015

1 galactosemias/
2 (galactosem* or ((galt utp hexose 1 phosphate uridyltransferase or gale or galk or galactokinase) adj3 deficien*)).tw.
3 1 or 2
4 Cataract/
5 Vitreous Hemorrhage/
6 exp Lens, Crystalline/
7 exp Vision Tests/
8 (vision or ophthalm* or crystalline or cataract* or ocular or vitreous).tw,kf.
9 or/4-8
10 3 and 9

Selection process

Diagnostics

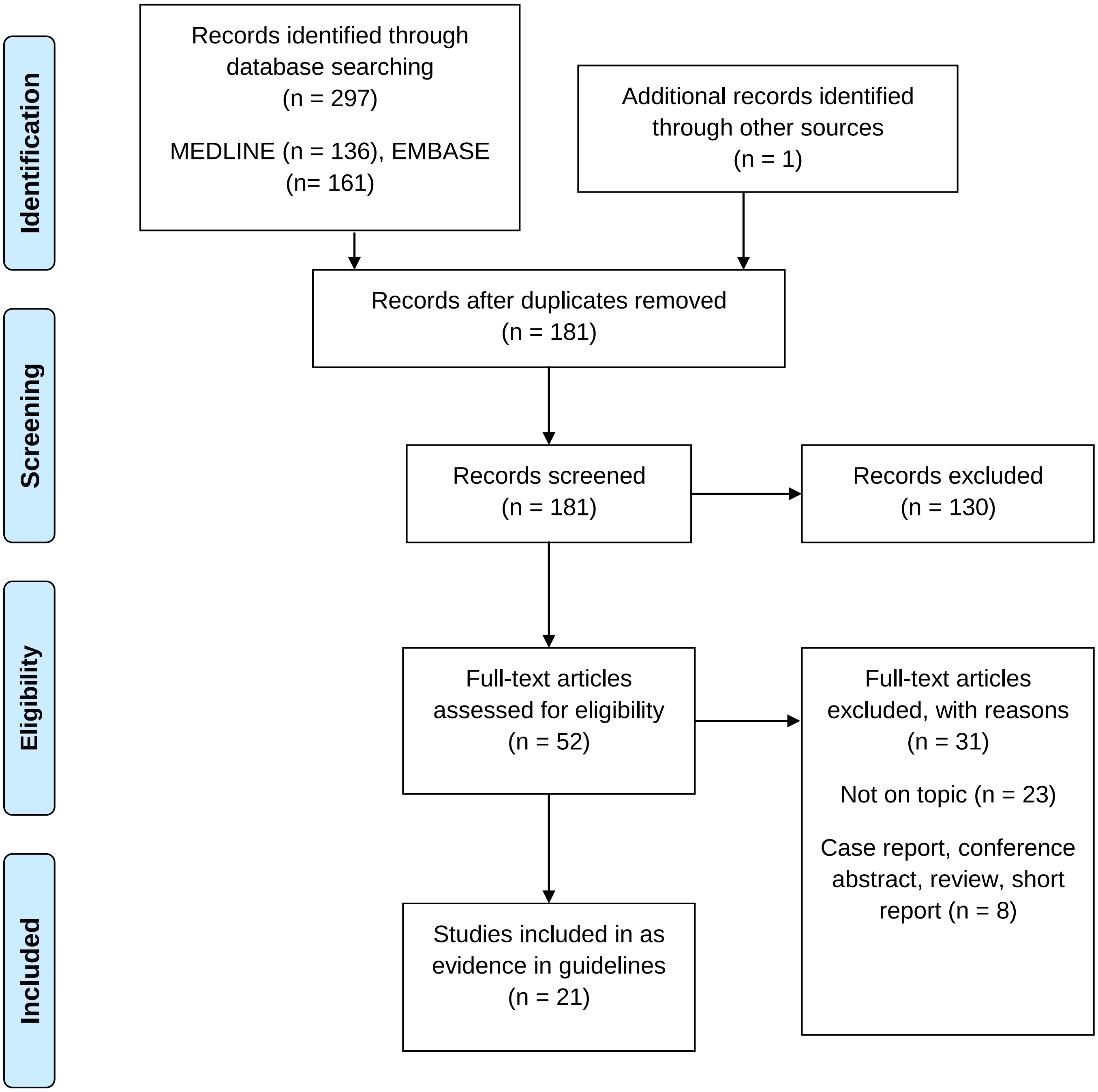

From: Moher D, Liberati A, Tetzlaff J, Altman DG, The PRISMA Group (2009). *P*referred *R*eporting *I*tems for *S*ystematic Reviews and *M*eta-*A*nalyses: The PRISMA Statement. PLoS Med 6(6): e1000097. doi:10.1371/journal.pmed1000097

Biochemical follow-up

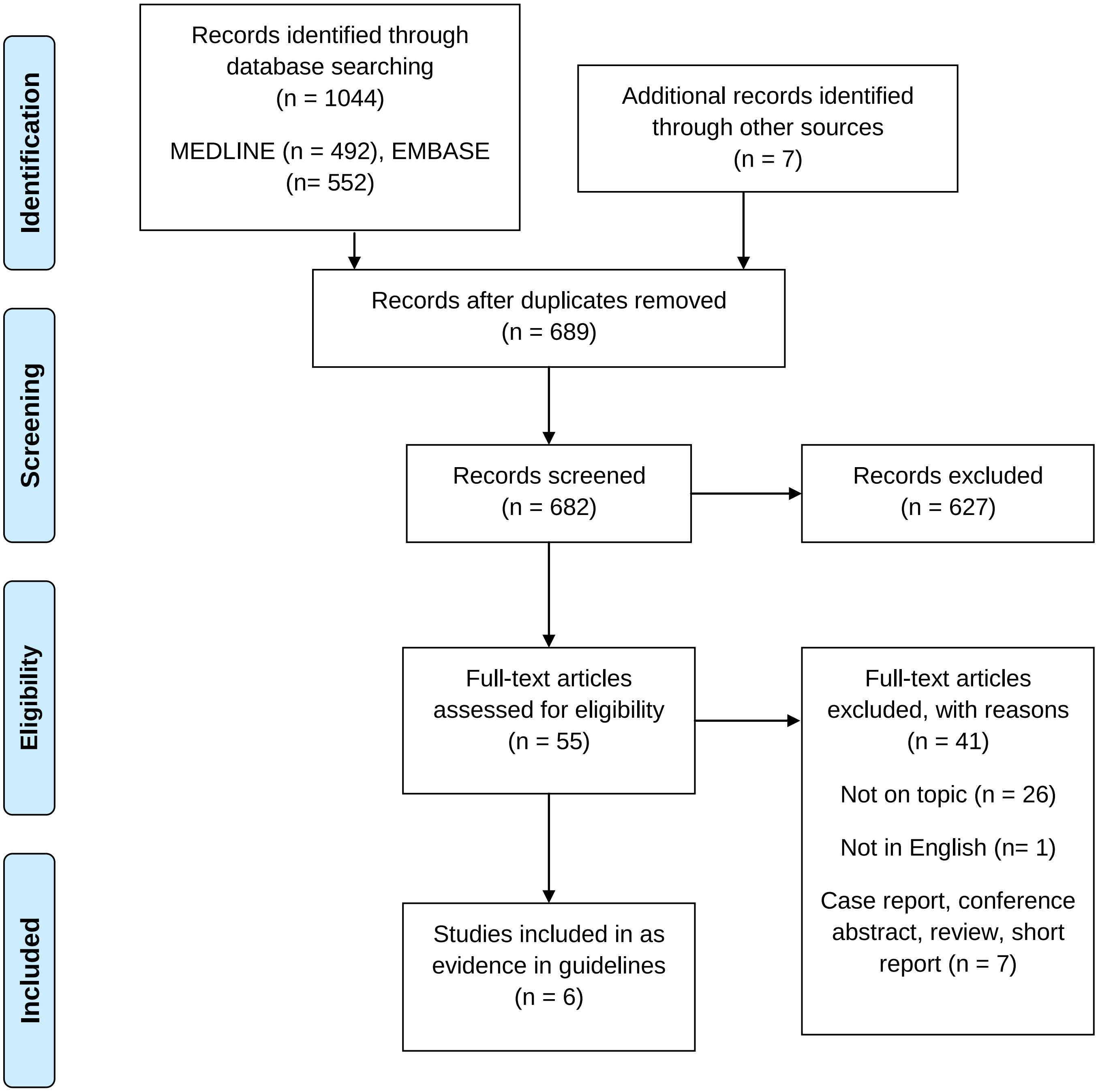

From: Moher D, Liberati A, Tetzlaff J, Altman DG, The PRISMA Group (2009). *P*referred *R*eporting *I*tems for *S*ystematic Reviews and *M*eta-*A*nalyses: The PRISMA Statement. PLoS Med 6(6): e1000097. doi:10.1371/journal.pmed1000097

Dietary management

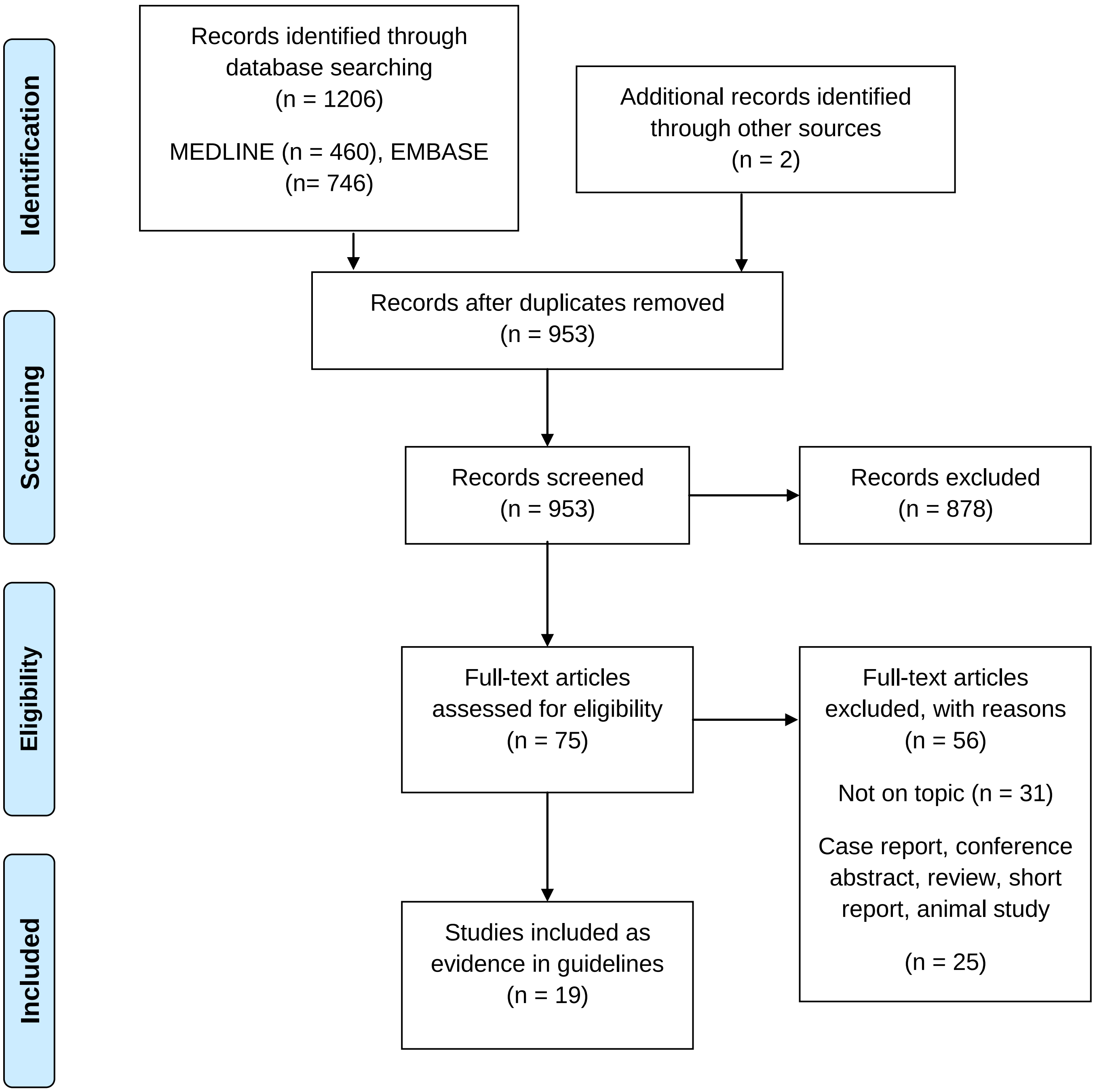

From: Moher D, Liberati A, Tetzlaff J, Altman DG, The PRISMA Group (2009). *P*referred *R*eporting *I*tems for *S*ystematic Reviews and *M*eta-*A*nalyses: The PRISMA Statement. PLoS Med 6(6): e1000097. doi:10.1371/journal.pmed1000097

Developmental follow-up/Speech and Language

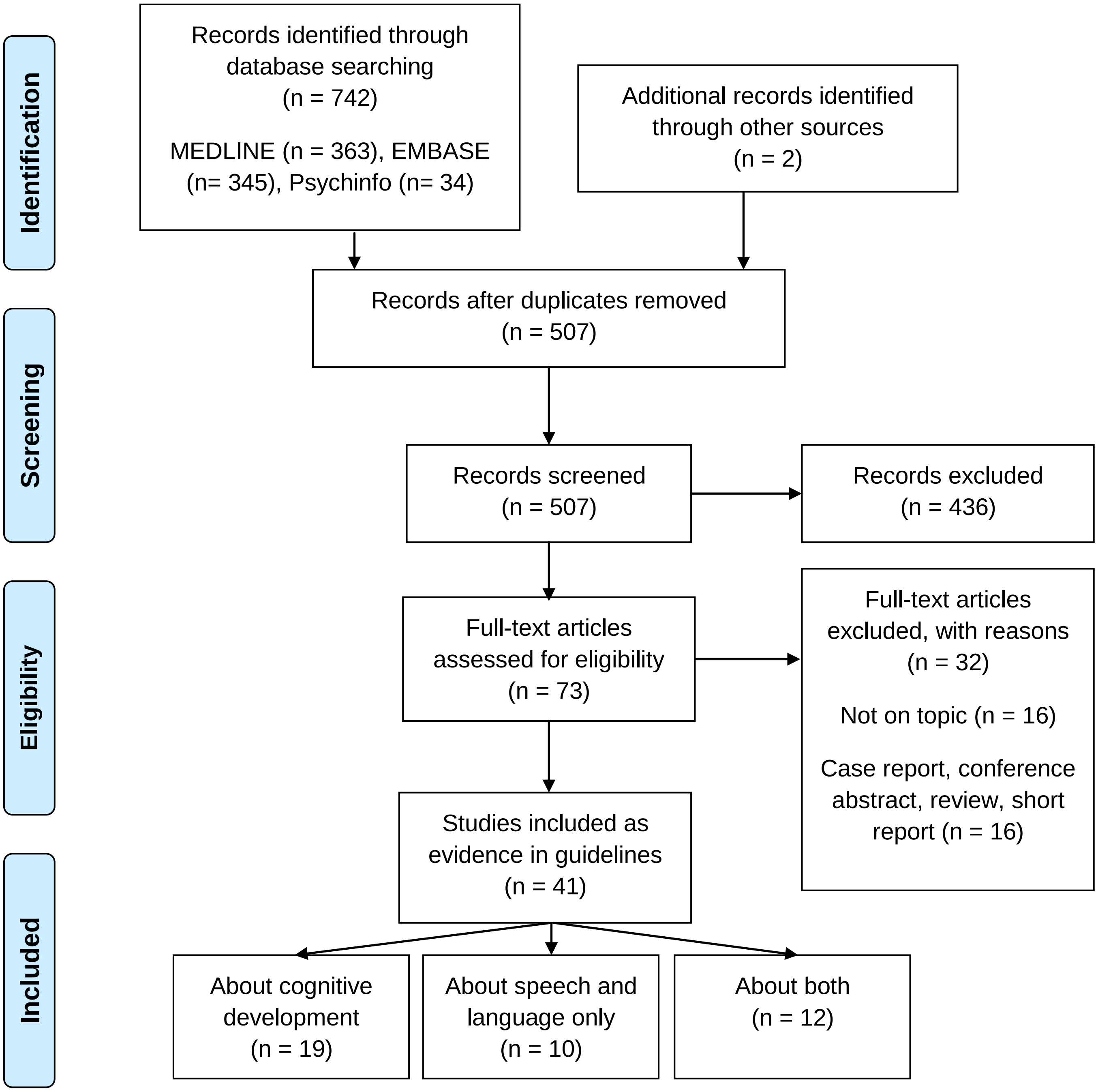

From: Moher D, Liberati A, Tetzlaff J, Altman DG, The PRISMA Group (2009). *P*referred *R*eporting *I*tems for *S*ystematic Reviews and *M*eta-*A*nalyses: The PRISMA Statement. PLoS Med 6(6): e1000097. doi:10.1371/journal.pmed1000097

Neurology

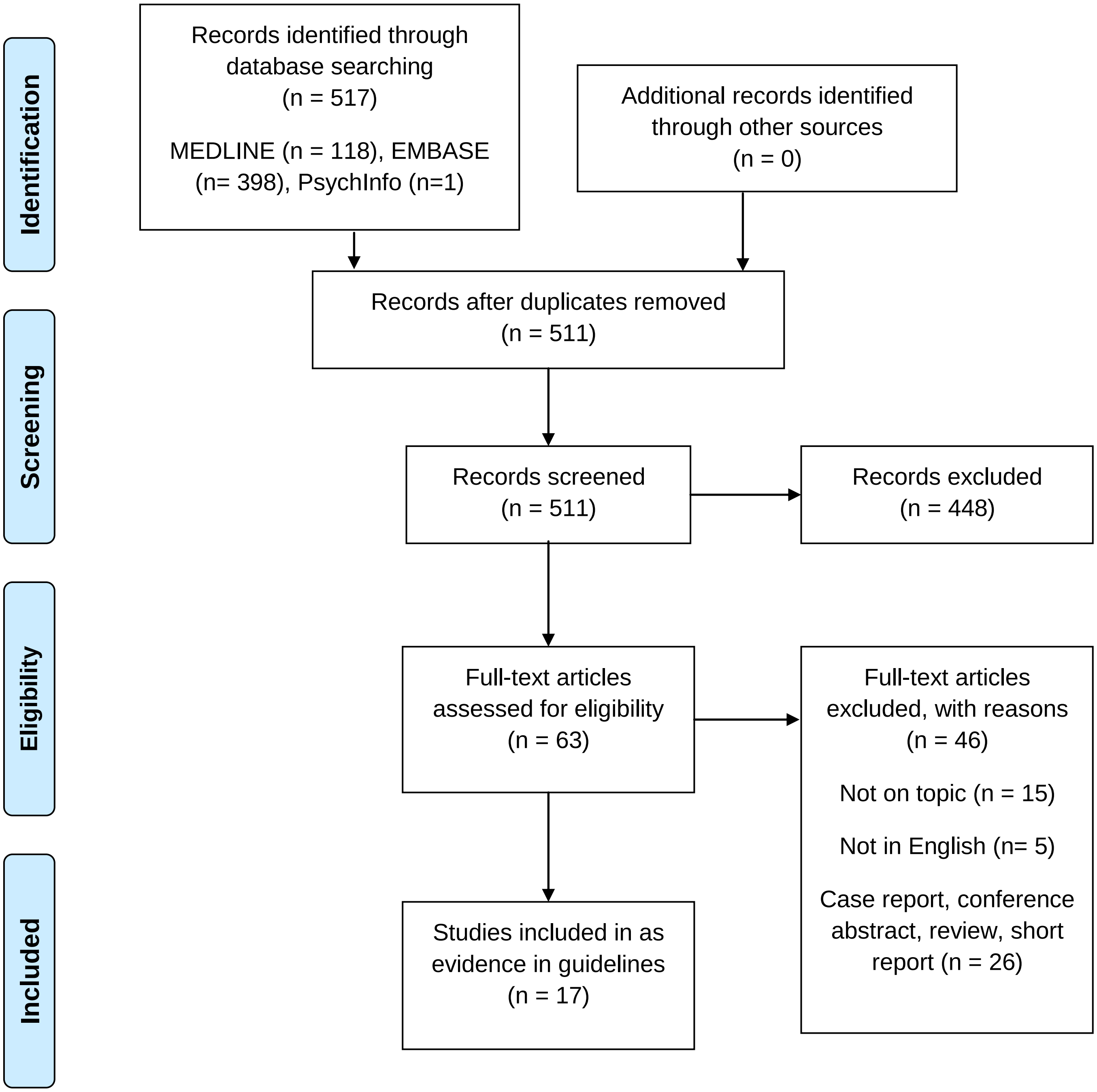

From: Moher D, Liberati A, Tetzlaff J, Altman DG, The PRISMA Group (2009). *P*referred *R*eporting *I*tems for *S*ystematic Reviews and *M*eta-*A*nalyses: The PRISMA Statement. PLoS Med 6(6): e1000097. doi:10.1371/journal.pmed1000097

Psychosocial development/Mental health

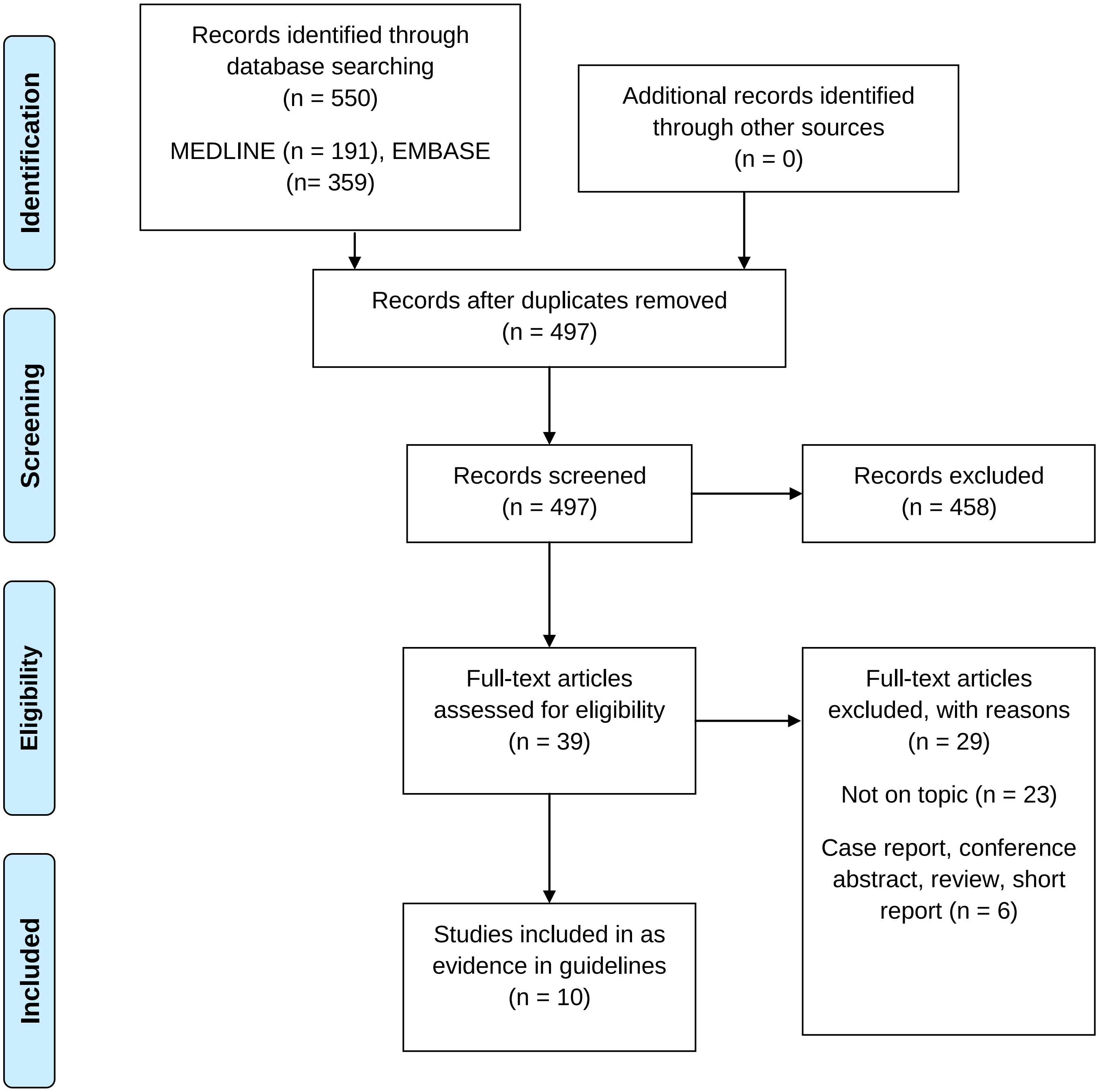

From: Moher D, Liberati A, Tetzlaff J, Altman DG, The PRISMA Group (2009). *P*referred *R*eporting *I*tems for *S*ystematic Reviews and *M*eta-*A*nalyses: The PRISMA Statement. PLoS Med 6(6): e1000097. doi:10.1371/journal.pmed1000097

Endocrinology/fertility follow-up

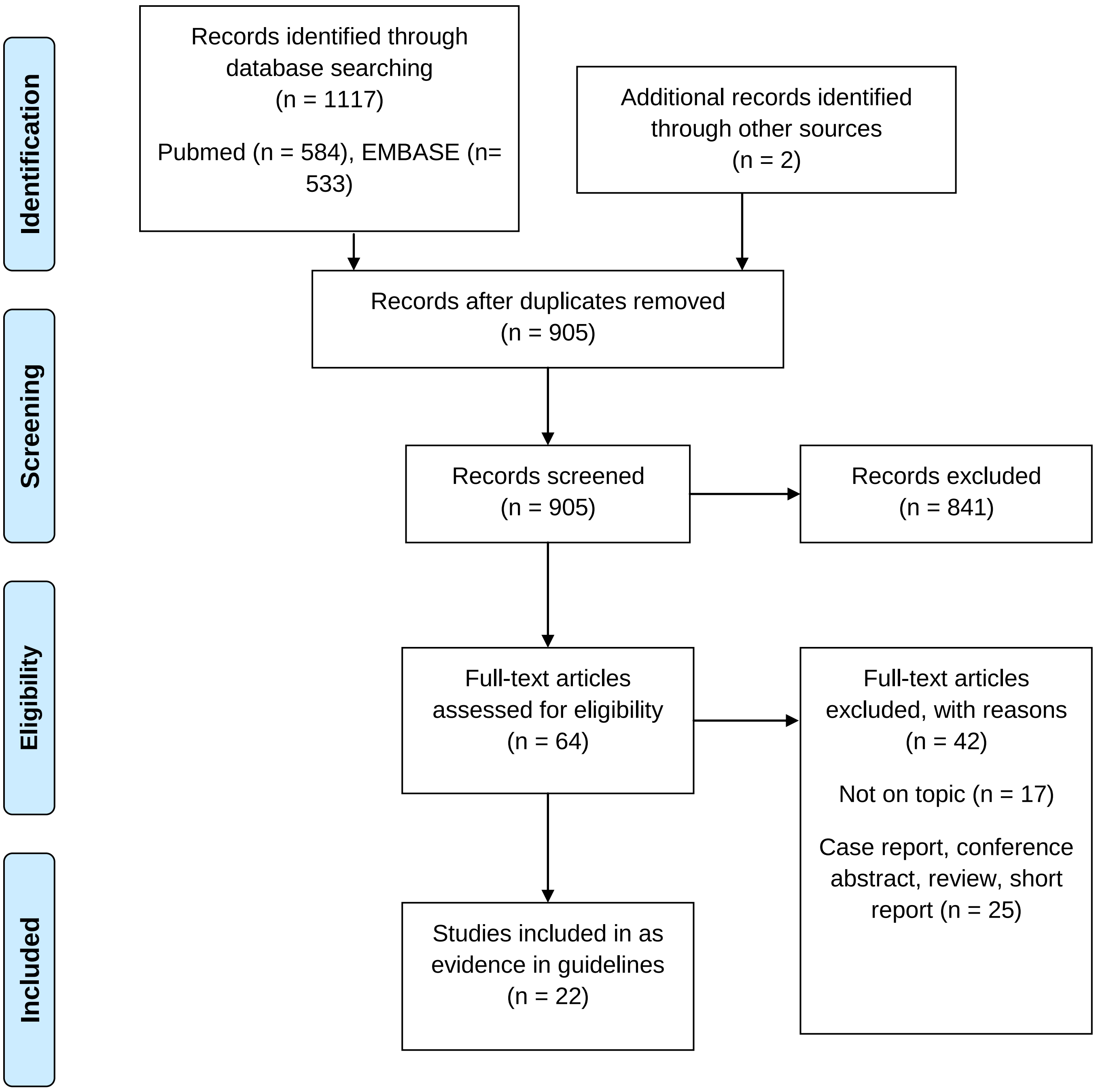

From: Moher D, Liberati A, Tetzlaff J, Altman DG, The PRISMA Group (2009). *P*referred *R*eporting *I*tems for *S*ystematic Reviews and *M*eta-*A*nalyses: The PRISMA Statement. PLoS Med 6(6): e1000097. doi:10.1371/journal.pmed1000097

Bone health

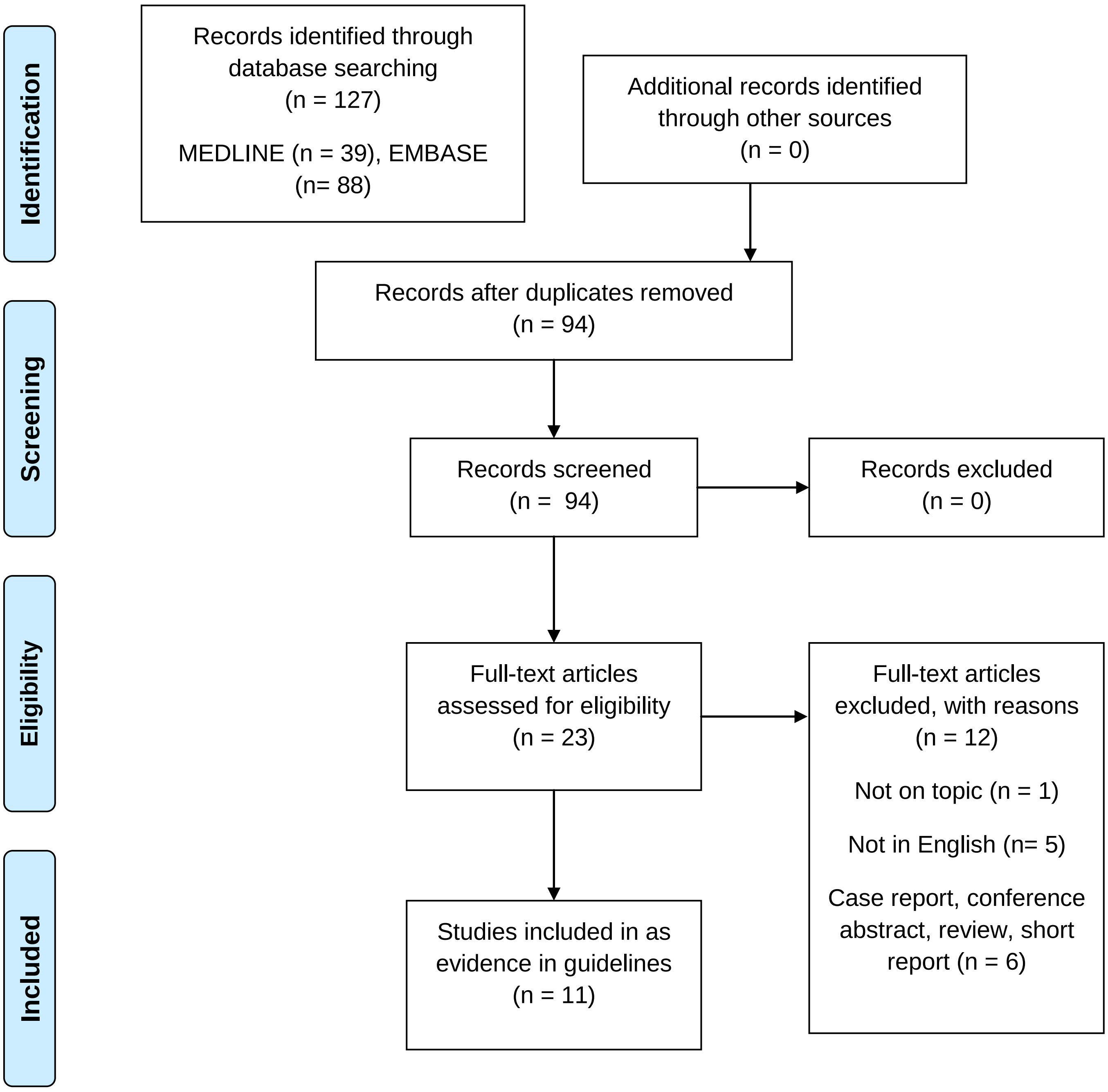

From: Moher D, Liberati A, Tetzlaff J, Altman DG, The PRISMA Group (2009). *P*referred *R*eporting *I*tems for *S*ystematic Reviews and *M*eta-*A*nalyses: The PRISMA Statement. PLoS Med 6(6): e1000097. doi:10.1371/journal.pmed1000097

Ophthalmological complications

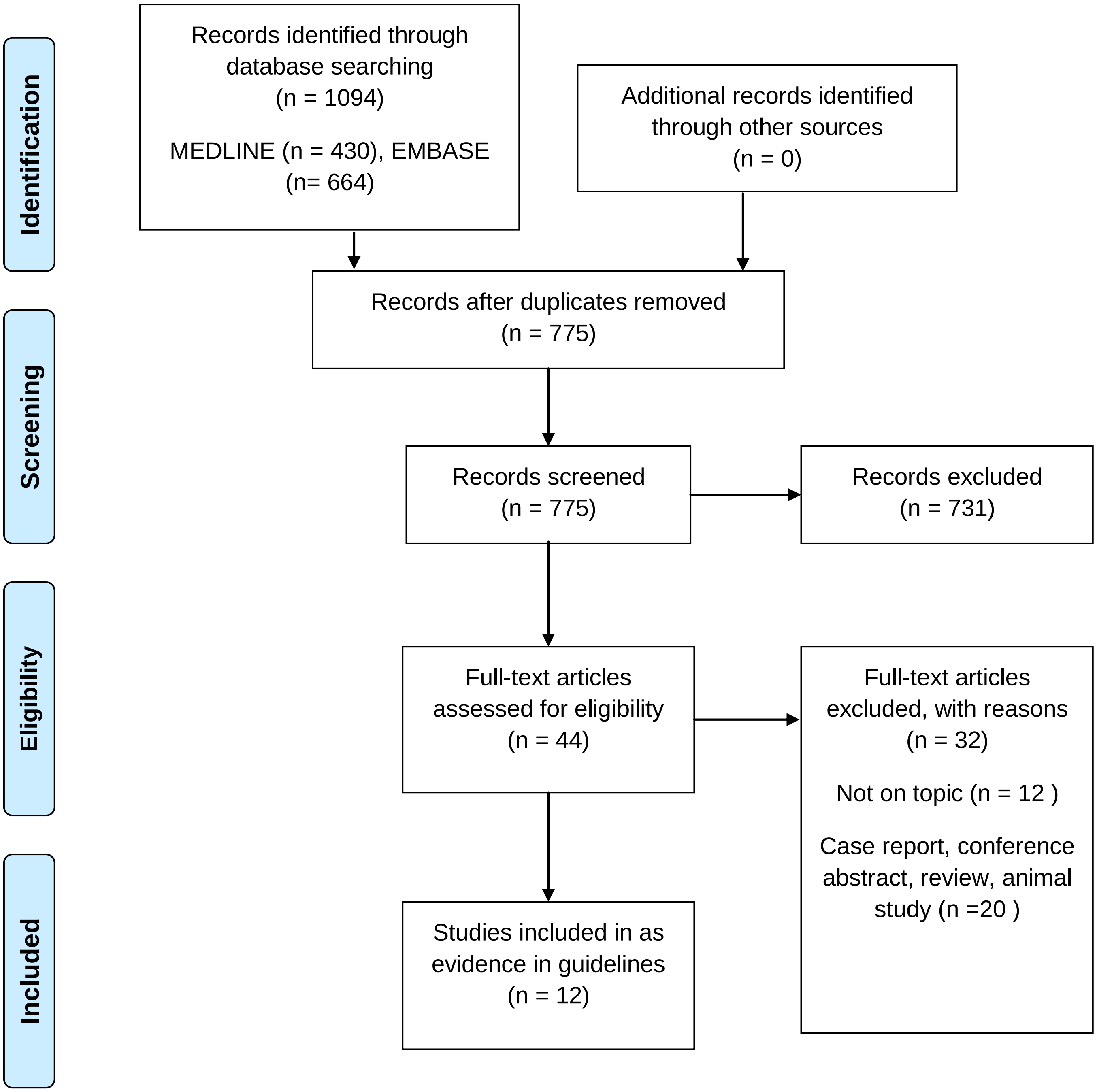

From: Moher D, Liberati A, Tetzlaff J, Altman DG, The PRISMA Group (2009). *P*referred *R*eporting *I*tems for *S*ystematic Reviews and *M*eta-*A*nalyses: The PRISMA Statement. PLoS Med 6(6): e1000097. doi:10.1371/journal.pmed1000097
