# Supplementary material for: International clinical guideline for the management of classical galactosemia: diagnosis, treatment, and follow-up
Source: J Inherit Metab Dis. 2016 Nov 17;40(2):171–6. doi: 10.1007/s10545-016-9990-5 (PMC5306419; doi:10.1007/s10545-016-9990-5)
Supplement: Supplementary file 3 — (PDF 688 kb) [file 10545_2016_9990_MOESM3_ESM.pdf]

Appendix 2 Summary of Evidence Tables

Abbreviations

1.25OHD:	1,25-dihydroxy vitamin D	IFWB:	Isoelectric Focusing/Western Blotting of transferrin
25OHD:	25-hydroxy vitamin D	ITPA:	Illinois Test of Psycholinguistic Abilities
AMH:	Anti-Müllerian hormone	IQ:	Intellectual quotient
AP:	Alkaline phosphatase	IV:	Intravenous
BAI:	Beck Anxiety Inventory	LDL:	Low-density lipoprotein
BDI:	Beck Depression Inventory	LH:	Luteinizing hormone
BMC:	Bone mineral content	Mg:	Magnesium
BMD:	Bone mass/mineral density	MRI:	Magnetic resonance imaging
BAP:	Bone-specific alkaline phosphatase	MRS:	Magnetic resonance spectroscopy
BSAG:	Bristol Social Adjustment Guide	MABC:	Movement Assessment Battery for Children
Ca:	Calcium	NTX:	N-terminal telopeptide
CDT:	Carbohydrate-deficient isoforms of transferrin	NBS:	Newborn screening
CG:	Classical galactosemia	NA:	Not applicable
cOC:	Carboxylated osteocalcin	Non-RCT:	Non-randomized controlled trial
CTX1:	Carboxy terminal telopeptide of type 1 collagen	OC:	Osteocalcin
CUMPCD:	Cumulative percentage dose	PTH:	Parathormone
DG:	Duarte galactosemia	PICU:	Pediatric intensive care unit
DHEAS:	Dehydroepiandrosterone sulfate	PIQ:	Performance intelligent quotient
DQ:	Developmental quotient	PMM2-CDG:	Phosphomannomutase 2-deficient congenital disorder of glycosylation
DXA:	Dual-energy x-ray absorptiometry		
EEG:	Electroencephalogram	P:	Phosphorus
ERT:	Estrogen replacement therapy	PKU:	Phenylketonuria
FDG-PET:	[18F]fluorodeoxyglucose positron emission tomography	POF:	Premature ovarian failure
FIQ:	Full-scale intelligence quotient	POI:	Premature ovarian insufficiency
FSH:	Follicle-stimulating hormone	RNI:	Reference nutrient intake
FT4:	Free T4 (=thyroxine)	RBC:	Red blood cell
Gal-1-P:	Galactose-1-phosphate	SHBG:	Sex-hormone binding globulin
GALT:	Galactose-1-phosphate uridyltransferase	SD:	Standard deviation
HDL:	High-density lipoprotein	TBG:	Thyroid-binding globulin
HH:	Hypergonadotropic hypogonadism	TSH:	Thyroid-stimulating hormone
HRQoL:	Health-related quality of life	ucOC:	Under-carboxylated osteocalcin
IGF-1:	Insulin-like growth factor 1	VIQ:	Verbal intelligence quotient
IGFBP-3:	Insulin-like growth factor binding-protein 3	Z:	Zinc

Recommendation #1

First author, year	#	*Study design*	*Study population (number & age)*	*Results*	*Remarks*
Calderon et al. 2007	1	Samples submitted for confirmatory testing for CG were analysed simultaneously for GALT enzyme activity and allele-specific PCR/fragment analysis for seven mutations and two polymorphisms in the GALT gene	Samples (n=243)	Mutation detection accorded with biochemical analysis in 93% of samples. Subsequently, a total of 34 samples with either discordant results between the above methods or low enzyme activity were fully sequenced, identifying previously reported pathogenic mutations and seven novel variations (p.P185H, p.R201C, p.E220K, p.R223S, p.I278N, p.L289F and p.L218X) in the GALT gene. This approach further increased concordance between genetic and biochemical analysis to 99% of all alleles tested	Mutations: IVS2–2A>G, p.S135L, p.T138M, p.L195P, p.K285N, p.Q188R, p.Y209C; polymorphisms p.N314D, p.L218L

Recommendation #2 (numbers 1 to 4 and 6 to 20)
Recommendation #3 (numbers 1, 2, 3, 4, 6, 7, 8, 9, 10, 15, 16, 17, 19, 20)

First author, year	#	*Study design*	*Study population (number & age)*	*Results*	*Remarks*
				Clinical outcome	
Badawi et al. 1996	1	Retrospective case series of long-term outcome	62 patients: 7 DG patients, sex unknown	All patients detected by newborn screening<br>7/7 DG patients asymptomatic at diagnosis	Long-term outcome reported only for CG patients
Badik et al. 2011	2	Cross-sectional study on AMH and FSH levels	57 female DG patients<br>Age <1 mnth and 10.5 yrs<br>64 female healthy controls<br>Age <1 mnth and 10.5 yrs	FSH and AMH levels in DG patients in neonatal period, ≥3 months to 18 mnths and ≥18 mnths to 10.5 yrs do not differ significantly from healthy controls	
Ficicioglu et al. 2008	3	Cross-sectional study on clinical, biochemical, and developmental outcomes	28 DG patients, 13 male, 15 female<br>Age 1-6 yrs (2.96 ± 1.31 yrs)	Group I and II: no ophthalmologic complications, no difference in FSH levels between group-I and -II subjects. Group 1 lower scores on adaptive scores, but same scores on language or overall IQ scores between<br>0/28: abnormal liver function at time of diagnosis or the study visit	**Group 1**: 17 DG patients (age range: 1.3–6 yrs, median 3.5 yrs) were on a lactose restricted diet in the first year of life<br>**Group II:** 11 DG children (age range: 1.1–5 yrs, median 2.28 yrs) were on regular diet since birth
Ficicioglu et al. 2010	4	Cross-sectional study on relationship of galactose metabolites to RBC Gal-1-P, dietary galactose intake, and neurodevelopmental/ clinical outcomes	30 DG patients, 15 male, 15 female<br>Age 1-6 yrs<br>20 healthy controls, age-matched	Mean values for the 3 outcome variables [adaptive, 103.9 (15.5); language, 109.9 (13.9); and full-scale IQ, 106.5 (10)] were within the reference interval<br>30/30: results in liver function tests and the eye examination within reference interval<br>No relationship between 3 developmental variables and RBC galactitol, galactonate, and Gal-1-P concentrations; plasma galactose and galactitol concentrations; urine galactitol and galactonate concentrations	17/30 on a lactose restricted<br>diet in the first yr of life<br>13/30 on a regular diet since birth

First author, year	#	Study design	Study population (number & age)	Results	Remarks
Milankovics et al. 2010	5	Retrospective case series and cross-sectional study of genotype, correlated to phenotype	Neonatal symptoms in:<br>5 DG patients, 4 male, 1 female<br>Age 2-39 yrs		**High risk of bias due to multiple pathology (cardiac disease, dysmorphic features) in the reported patients, therefore not included as evidence**
Powell et al. 2009	6	Retrospective case series on developmental disabilities and other developmental outcomes that require special education services in childhood in DG children	75 DG patients, age 3-10 yrs	All DG children were treated with a galactose-restricted (primarily by lactose restriction) diet until 1 yr. Five (8.5%) DG children within the 3 to 10 yrs age range were identified as having received special education services, compared to 4.5% in general population (difference not significant). Of all 8 year olds 5 DG patients (15.2%) received special education (in general population 5.9%). 2/5 speech/language disorder, 3/5 assumed to have speech or language disorder	
Schweitzer et al. 1993	7	Case series and cross-sectional study on clinical outcomes	134 patients of which 5 DG patients, sex unknown	5/5: diagnosed as having CG due to positive newborn screening results without clinical symptoms	
			**Galactose metabolism and oxidation**		
Berry et al. 1995	8	In vivo galactose oxidation	2 DG patients, both female<br>Age 6 and 29 yrs<br>7 CG patients, 2 male, 5 female<br>Age 6-31 yrs<br>10 healthy controls, 6-37 yrs	Percentage of dose eliminated as 13CO2 in expired air after 1 and 5 hours respectively<br>Controls: 3-6 and 21-47<br>DG patients: 3-4 and 27-32<br>CG patients: 0.03-0.31 and 1.2-3.6	2 baseline breath samples, then IV bolus of [1-13C]galactose administered

First author, year	#	Study design	Study population (number & age)	Results	Remarks
Berry et al. 1997	9	In vivo galactose oxidation	7 CG patients, 2 male (age 6 and 30 yrs), 5 female (age 7-31 yrs)<br>1 patient with S135L/S135L variant, female, age 12 yrs<br>1 DG patients, female, age 6 yrs<br>1 patient with N314D/N314D, female age 29 yrs<br>1 Q188R heterozygote, female 35 yrs<br>1 S135L heterozygote, female 37 yrs<br>10 controls (6 adults 18-37 yrs, 4 children 6-13 yrs)	Percentage of dose eliminated as 13CO2 in expired air:<br>After 1 hour and 5 hours<br>CG patients: 0.03-0.31 and 1.2-3.6<br>S135L/S135L patient: 2 and 19<br>DG patient: 3 and 27<br>N314D/N314D: 4 and 32<br>Q188R heterozygote: 3 and 28<br>S135L heterozygote: 4 and 31<br>Control adults: 3-6 and 27-47<br>Control children: 4-5 and 21-40	IV bolus of [1-13C] galactose followed by a continuous infusion of [1-13C]galactose
Berry et al. 2000	10	In vivo whole body galactose metabolism	37 CG patients, 15 male, 22 female<br>Age 3-48 yrs<br>20 control subjects, 6 male, 14 female Age 3 -37 yrs	Patients were defined severe if elimination <2% at 2h. These patients were Q188R/Q188R (>50%) or Q188R/ (L195P, K285N, E308K, V151A, Q344K, M142K), 1 patient Q188R/UNK, 1 K285N/UNK<br>Patients were defined variant if elimination >2% at 2h. Variants were divided in 2 groups:<br>Group 1 variant (n=9): no enzyme activity in RBC. Elimination at 2h 8-22%, which overlapped with the normal range. No patients with Duarte allele except 1 patient with IVSC mutation in cis with N314D. 1 homoallelic S135L, 5 with 1 S135L with missence, 1 patient R258/Y209C subject with greater than 5% residual activity<br>Group 2 (n=15): RBC enzyme activity 10-25%, elimination at 2h 7.3-25.85 which overlapped with the normal range. All compound heterozygotes with the Duarte N314D allele plus second CG allele such as Q188R or K285N	Both PO and IV breath tests<br>IV breath tests. Based on the % of elimination of [1-13C]galactose dose as 13CO2 at 120 min

First author, year	#	Study design	Study population (number & age)	Results	Remarks
Lai et al. 1996	11	In vivo galactose oxidation	1 patient S135L/S135L, female, age 14 yrs<br>1 patient Q188R/Q188R, female, age14 yrs<br>1 healthy control, female, age 14 yrs	Galactose oxidation:<br>CUMP at 300 min for D-[1-13C]/ D-[2-13C] galactose respectively<br>Q188R/Q188R: 3.54/4.87<br>S135l/S135L: 18.89/26.213<br>Control: 21.09/28.87	Administration of galactose. D-[1-13C]-Galactose IV (7 mg/kg) on day l. On day 2, after a second 12-hour fast, administration of D-[2-13C]-galactose
Wehrli et al. 2002	12	In vitro study of galactose metabolism in lymfoblasts	2 patients with S135L/S135L<br>6 patients with Q188R/Q188R<br>Sex and age unknown<br><br>10 healthy controls	After incubation with 1-13C galactose no difference observed in gal-1-p levels between Q188R/Q1888R and S135L/S135L lymphoblastic cells, but both had significantly higher Gal-1-P levels compared to control cells<br>UDPgal level in the S135L/S135L cells significantly lower than in controls, no significant difference in the UDPglu levels. No difference in the UDPgal and UDPglu levels between S135/S135L and Q188R/Q188R	
Yager et al. 2001	13	In vitro study of galactose oxidation	Lymphoblast cell lines of 15 CG patients, age 2-31 yrs<br>6 Q188R/Q188R<br>5 Q188R/other<br>2 S135L/S135L<br>1 S135L/H315H<br>1 del/del	Lymphocytes transformed with EBV virus<br>All patients absent RBC GALT activity<br>Oxidation of [1-14C]galactose by cultured lymphoblasts after 2.5h/5h of incubation (% of normal):<br>Q188R/Q188R: 1.6-4.3%/6.8-9.8%<br>Q188R/Other: 2.6-13.7%/9.1-17.9%<br>S135L/S135L: 15.3-17.3%/15.9-19.5%<br>S135L/H315H: 4.0%/13.0%<br>Del/del: 5.7/6.1%	
			**Biochemical parameters**		

First author, year	#	Study design	Study population (number & age)	Results	Remarks
Coss et al. 2014	14	Cross-sectional studies on N-glycan abnormalities	1 S135L/S135L patient, male, age 4 yrs<br>9 Q188R/Q188R patients, 3 male, 6 female,<br>age 11 mnths-9 yrs	The S135L/S135L patient, on a galactose intake of 300 mg/day, had high G-ratios similar to the CG patients on diet of <50 mg galactose/day.<br>All G-ratios significantly higher for Q188R/Q188R patients compared with the healthy controls. High G0/G1, G0/G2, and (G0/G1)/G2 ratios indicate a higher level of agalactosylated structures	G-ratios are informative indicators of the level of galactose incorporation<br>Determined ratios: G0/G1, (G0/G1)/G2 and G0/G2<br>G0 = agalactosylated<br>G1 = monogalactosylated<br>G2 = digalactosylated
Ficicioglu et al. 2005	15	Retrospective case series/cross-sectional study on biochemical phenotype of dietary treated<br><br>and untreated DG patients detected during<br><br>newborn screening	Samples of:<br>Group I: 22 DG patients, newborns<br>14/22 (age range 7–60 days, median 14 days) on regular diet, 8/22 (age range 9– 69 days, median 22.5 days) on lactose-free formula<br><br>Group II: 18 DG patients, age 12-18 mnths, put on a regular diet after a yr of galactose restriction<br><br>Group III: 18 healthy controls, age 9 dys-20 mnths<br>Group IV: 12 age-matched CG patients, age 7-20 mnths, all Q188R/Q188R	RBC galactitol, galactonate and Gal-1-P were much higher in DG newborns on regular formula/breast milk than in in DG newborns on dietary treatment and normal control newborns<br>No difference in RBC galactitol, galactonate and Gal-1-P between DG newborns on restricted diet and the control group<br>DG children on regular diet after a yr of lactose restriction: 8/18 higher RBC galactitol and galactonate levels than healthy controls, 10/18 RBC galactitol and galactonate comparable to healthy controls. Levels of Gal-1-P comparable between these 2 groups<br>DG subjects >1 yr of age who responded to a normal diet: galactitol values comparable to CG patients on a diet, RBC galactonate 7-fold higher than in CG patients, Gal-1-P in RBC in the normal range (while elevated in CG)<br>Ratio of galactitol to galactonate <1 in RBC of all of the D/G patients and healthy controls, in CG the ratio was above >1 (1.74± 0.11)	

First author, year	#	Study design	Study population (number & age)	Results	Remarks
Ficicioglu et al. 2008	16	Cross-sectional study on clinical, biochemical, and developmental outcomes of DG patients who received a galactose free diet in the first yr of life and those on an unrestricted diet	28 DG patients, 13 male 15 female<br>Age 1-6 yrs (2.96 ± 1.31 yrs)<br>**Group 1**: 17 DG patients (age range: 1.3–6 yrs, median 3.5 yrs) were on a lactose restricted diet in the first year of life.<br>**Group II:** 11 DG children (age range: 1.1–5 yrs, median 2.28 yrs) were on regular diet since birth	DG newborns on regular diet: higher levels of RBC Gal-1-P, and urine galactitol than those on diet. Very high levels of RBC Gal-1-P gradually decreased without any dietary interventions during the first year of life. At age 1 yr, untreated DG patients had near normal levels of RBC Gal-1-P and urine galactitol<br>DG patients on a lactose restricted diet in the first yr of life: normal RBC Gal-1-P and urine galactitol levels during infancy	25/28: N314D/Q188R<br>3/28:N314D and S135L or K285N or deletion
Ficicioglu et al. 2010	17	Cross-sectional study of relationship of galactose metabolites to RBC<br>Gal-1-P, dietary galactose intake, and neurodevelopmental/ clinical outcomes	30 DG patients, 15 male, 15 female, age 1-6 yrs<br>20 healthy controls, age-matched	17/30 had previously been on a lactose restricted diet in the first year of life<br>13/30 had been on a regular diet since birth<br>Compared to controls DG patients have a 6-fold higher mean RBC galactonate concentration, a 2-fold higher mean RBC galactitol concentration, a 2-fold higher mean plasma galactose concentration, and a 2-fold higher mean plasma galactitol concentration. Gal-1-P values are higher than in controls, but are in the reference range. Increased levels of other galactose metabolites, including RBC galactitol and galactonate, that correlate with galactose intake	Data for RBC Gal-1-P and urine galactitol were<br>included in a prior report

First author, year	#	Study design	Study population (number & age)	Results	Remarks
Landt et al. 1997	18	Cross-sectional study of GALT activity levels and<br>GALT protein content in correlation to genotype and clinical phenotype in black patients	8 black CG patients, sex unknown<br>Age 2-18 yrs<br>2/8: S135L/S135L<br>2/8: S135L/Q188R<br>3/8: S135L/unknown<br>1/8: Q188R/Q188R<br><br>7 white patients, sex and age unknown<br><br>10 heterozygotes<br><br>14 healthy controls	S135L in 9/16 alleles of black patients and 0/14 alleles of white patients. Q188R allele in 10/14 white patients and in 4/16 black patients<br>**RBC GALT activity**: no clear difference between specific mutations (Q188R, S135L, unknown)<br>**Leukocyte GALT activity**: S135L/S135L patients much higher residual GALT activity than other genotypes. Activity in patients with S135L/unknown or S135L/Q188R not significantly different from all non-S135L allelic CG children<br>No clear pattern of association of the S135L allele with mild disease	
Palmieri et al. 1999	19	Cross-sectional study of urinary and plasma galactitol	Samples of 9 patients compound heterozygotes for N314D, age 0-36 yrs<br>2 patients N314D/N314D, 0-31 yrs<br>4 patients S135L/S135L, 4 patients S135L/other, age 0-34 yrs<br>67 CG patients, age 0-47 yrs<br>95 healthy controls, age 0-45 yrs	Normal range of urine galactitol was age-related in healthy controls<br>All S135L/S135L patients had lower galactitol excretion than Q188R/Q188R or Q188R/other patients, levels were still above the normal range<br>Except for 4 patients in the 1 to 6-year age group, patients with N314D/Q188R, as well as N314D/other, had galactitol excretion rates in the normal range	
Schwarz et al. 1985	20	Cross-sectional study on urinary galactitol and galactose excretion after oral galactose challenge	24 DG patients<br>Age 0.3-9.7 yrs (mean 3.5 yrs, median 2.2 yrs)<br>16 healthy controls, age 2-10.7 yrs (mean 5.9 yrs, median 5.4 yrs)	1 gram D-galactose oral, urine collected for 3 hours<br>Most DG patients excreted considerably more galactitol and galactose than controls. More pronounced differences with increasing age. Urinary excretion of galactose and galactitol increased linearly with age in all subjects after galactose load, but was much higher for patients than controls	The 6 patients < 2 yrs on a galactose-restricted diet, the others on a normal diet

Recommendation #4

First author, year	#	*Study design*	*Study population (number & age)*	*Results*	*Remarks*
Schweitzer et al. 2003	1	Retrospective study to evaluate CG patients born before and after introduction of NBS	49 patients were born between 1955 and 1977 before NBS for CG had been introduced in former West-Germany and 99 patients were born afterwards	Of 20 deceased patients, 19 died between 1955 and 1977 due to their underlying disease, 15/19 before the diagnosis was established, 2/19 after start of diet. 4 patients died between 7–14 days of age and 5 between 15–21 days. 9 patients survived longer but died between 4-9 weeks of age and 1 patient at the age of 3 yrs. In the screened group, only 1 patient died (1980, even after galactose restriction)<br>33/49 patients born before 1978, CG had been suspected due to clinical symptoms<br>28/ 99 patients born after 1978 had been suspected of having CG because of clinical abnormalities before the positive NBS test result was received	

Recommendation #5 (numbers 1, 2, 3, 4, 5, 6, 7, 8, 9)
Recommendation #6 (numbers 1, 2, 3, 4, 5, 6, 9)

First author, year	***#***	***Study design***	***Study population (number & age)***	***Results***	***Remarks***
**Berry et al. 1993**	1	Experimental study to evaluate the effect of dietary fruits and vegetables on urinary galactitol excretion in	2 CG patients, 1 female 26 yrs, 1 male 18 yrs	Galactitol excretion on a lactose-free diet (intake of galactose: 4-27 mg per day):<br>Patient 1: 9 month period range 128-165 µmol/mmol creatinine<br>Patient 2: 2.5 month period range 121-155 µmol/mmol creatinine<br>3 weeks exposure to 200 mg of galactose per day<br>Urinary galactitol excretion baseline-> 3 weeks: Patient 1: increase 17%, patient 2 increase 14%.<br>No effect on RBC galactose-1-phosphate levels<br>Patient 1: galactose enriched diet from fruit and vegetables for 3 weeks: 54 SD 11 mg/day galactose<br>Galactitol: 1591->1647 umol/day	
**Bosch et al. 2004**	2	Experimental study to determine the tolerance for exogenous galactose	3 CG patients, 2 females (age 15 and 18 yrs), 1 male (age 16 yrs)	Total daily dietary galactose intake: 17-22 mg during the usual galactose restricted diet, 11-15 mg during the period of the very strict galactose restricted diet<br>All but 1 RBC Gal-1-P values remained below the upper limit of the recommended range (0.58 mmol/g Hb). In patient 2 one unexpected high value of 0.80 mmol/g Hb was measured one week after returning to her normal diet (ingestion of cows' milk two days earlier). 3 weeks later it returned to a low value of 0.38 mmol/g Hb<br>No physical or ophthalmological abnormalities detected	During weeks 1 and 2: patients remained on their regular galactose restricted diet. From week 3-10: all patients on a very strict galactose restricted diet. From week 5: patients received an oral dose of galactose. Weeks 5 and 6 200 mg, weeks 7 and 8 400 mg, weeks 9 and 10 600 mg

First author, year	#	*Study design*	*Study population (number & age)*	*Results*	*Remarks*
**Coss et al. 2012**	3	Experimental study to investigate whether differential glycosylation patterns in IgG N-glycans could provide a more informative clinical marker in CG compared to the current biochemical tests	10 CG patients, 8 male, 2 female<br>Age 18–26 years, 5 included in tests and 5 as controls	No clinical changes in the diet relaxed group on physical examination; ophthalmology assessments, coagulation testing, biochemical, clinical or psychological evaluations.<br><br>Mean RBC Gal-1-P and urinary galactitol in:<br><u>CG controls on a galactose intake of <300 mg/day:</u><br>Gal-1-P mean 0.55 μmol/gHb (r=0.43–0.77 μmol/gHb, n=15)<br>Galactitol mean 124.4 μmol/mmol creatinine (range 69–216 μmol/mmol creat, n=15)<br><u>Diet relaxed group:</u><br>G1P mean 0.51 μmol/gHb (range 0.33–0.81 μmol/gHb, n=21)<br>Galactitol mean 120.2 μmol/mmol creatinine (range 65–209, n=21)<br>No significant change in Gal-1-P levels or galactitol levels with each increment	Both groups on galactose restricted diet<br>Galactose relaxation up to 4000 mg over 14 weeks
**Gitzelmann et al. 1965**	4	Experimental study to assess the handling raffinose and stachyose	1 CG patient, male, age unknown (boy)<br>1 healthy control, male, age unknown (boy)	CG patient: galactose restricted diet. After control period of 7 days: raffinose supplements of 6.6g and 13.2g (added to main meals) for 5.5 and 6 consecutive days respectively. Gal-1-P unchanged after 5 and 10 mg stachyose, and after prolonged administration of raffinose 3 times 4 gram/day<br>Healthy control: after ingestion of raffinose or melibiose: blood galactose and Gal-1-P unchanged	

First author, year	*#*	*Study design*	*Study population (number & age)*	*Results*	*Remarks*
**Huidekoper et al. 2005**	5	Experimental study to assess the effect of exogenous galactose on galactose appearance rate	2 CG patients, 1 female 21 yrs, 1 male 29 yrs<br>3 healthy controls, 2 female, 1 male<br>Age 26-35 yrs	GAR is significantly lower in healthy control subjects than in CG patients:<br>GAR1 (initial rate): patients 2.44-2.48, controls 0.34-0.46<br>GAR2 (doubled galactose infusion): patients 2.13-2.43, controls 0.38-0.57<br>No significant differences between GAR in the initial steady state (GAR1) and GAR during doubled or quadrupled galactose infusion (GAR2 and GAR4) in both patients with classical galactosemia as well as in healthy subjects (endogenous production not affected by exogenous galactose intake)	GAR in µmol/kg/h
Krabbi et al. 2011	6	Retrospective case series on long-term complications and results of urinary galactose/galactitol excretion in patients in Estonia who have been on a less restricted diet since the suspicion and confirmation of the diagnosis	Galactose and galactitol in:<br>23 preserved urine samples from 5 CG patients<br>1 male, 4 female, age at study 7-14 yrs<br>Age at testing unknown	Galactose: range 60-600 mmol/mol creat (normal 4–6; pathological value >10), which is 10–100 times higher than the reference range.<br>Galactitol: range 70-1200 mmol/mol creat (normal 2–4, pathological value >10), also 17–300 times higher than the reference range<br>Concentrations of urinary galactose and galactitol varied from one sample to another	Patients were on a less restricted diet (no restriction of mature cheeses, fruits and Vegetables) since the suspicion and confirmation of the diagnosis
**Schadewaldt et al. 2004**	7	Experimental study to assess age dependence of endogenous galactose formation in patients homozygous for Q188R mutation	18 CG patients, 6 male, 12 female<br>Age 4.4-36.6 yrs<br>7 healthy controls, 6 male, 1 female, age mean 25 yrs	Endogenous galactose rate of appearance in µmol/h per g body weight<br>CG patients: range 2.05-4.58<br>Controls: mean 0.29±0.04<br>Highly significant inverse correlation between galactose appearance rate as well as galactitol and galactonate excretion and age	IV priming dose of 8 µmol D-[1-13C]galactose per kg body weight , thereafter 0.8 µmol D-[1-13C]galactose per kg body weight per h infused continuously

First author, year	#	*Study design*	*Study population (number & age)*	*Results*	*Remarks*
**Schadewaldt et al. 2014**	8	Experimental study to asses endogenous galactose release in a group of non-Q188R homozygous patients	17 CG patients, 9 male, 8 female<br>Age 4-34 yrs<br>7 controls, 6 male, 1 female<br>Age mean 25 yrs SD 3 yrs	16/17: compound heterozygous genotype<br>Endogenous galactose rate of appearance (in mmol/h per kg body weight): patients range 1.73-4.61. Controls: mean 0.29±0.04.<br>Exponential decrease of endogenous galactose release with age until adulthood (from 9.6 µmol/kg/body weight per h in newborns to 3.4 µmol/kg body weight in adults, equivalent to about 14 and 42 mg galactose equivalents/kg body weight per day respectively)	Priming dose D-[1-13C]galactose 8 mmol/kg body weight, continuous infusion 0.8 mmol/kg body weight per h)
**Wiesmann et al. 1995**	9	Experimental study to evaluate the effect of a raffinose- and stachyose-poor diet for a period of 14 days	6 CG patients, sex unknown, age 6-24 yrs	5/6 somewhat reduced red cell Gal-l-P during raffinose- and stachyose-poor diet of 14 days, however the decrease was statistically insignificant<br>Plasma galactose concentrations: unchanged	

Recommendation #7

First author, year	#	*Study design*	*Study population (number & age)*	*Results*	*Remarks*
El-Bassyouni et al. 2006	1	Cross-sectional study to evaluate the effect of diet on anti-oxidant status	8 patients, 7 male, 1 female, age unknown, 5 CG patients, 3 GALE patients<br>10 healthy age and sex matched controls	Significant lower copper, Ca, P, selenium, manganese and β-carotene compared to controls	
Gajewska et al. 2006	2	Cross-sectional study to evaluate bone turnover markers in prepubertal patients	35 CG patients, 17 male, 16 female<br>Age 1-10 yrs	Normal levels of (compared to reference range):<br>Ca: mean 2.27 mM (range 2.27-2.77)<br>P: mean 1.65 mM (range 1.32-1.92)<br>25-OHD: mean 31.6 μg/L (range 17.3-59.3)	
Gajewska et al. 2008	3	Cross-sectional study to assess bone formation and<br>resorption processes with bone turnover markers in children and adolescents	62 CG patients, 32 male, 30 female<br>Age 2-20 yrs	In children and adolescents normal median values of:<br>Ca: 2.46 mM (2.20-2.77)<br>P: 1.56 mM (1.01-1.92)<br>25OHD: 27.3 μg/L (17.2-59.3)	
Panis et al. 2004	4	Cross-sectional study to evaluate bone metabolism	40 CG patients, 13 male, 327 female<br>Age 3.0-17.3 yrs, mean 8.9±4.1 yrs	The recommended dietary allowances for Ca, P, Mg, Z, vitamin D, and protein were met in all patients. They all routinely used vitamin- and mineral-enriched soy products.<br>Normal levels of:<br>Ca: 2.4±0.1 mmol/l (range 2.3-2.6)<br>1.25OHD: 154.3±44.3 pmol/l (range 100-284)<br>Also normal levels of Mg and Z	
Panis et al. 2006	5		Suspected major overlap with cohort of Panis et al. 2004		
Rubio-Gozalbo et al. 2002	6	Cross-sectional study to measure BMD and to assess blood parameters involved in bone formation and resorption	11 CG patients, 5 male, 6 female<br>Age 2.5-18 yrs	Dietary Ca intake for all patients appeared sufficient for all subjects, mean 839 mg/day, range 500-1070 mg<br>Normal serum concentrations of Ca, P and vitamin D	

First author, year	#	Study design	Study population (number & age)	Results	Remarks
Rutherford et al. 2002	7	Cross-sectional study on dietary calcium intake	19 CG patients, 5 male, 14 female, 7 children, 12 adults, age unknown	RNI for calcium was met only in 26% of patients and in 10 the total Ca intake was less than 70% of the RNI. Ca requirements of the patients could not be met by diet alone as the median Ca intake from non-milk sources was only 44% of the RNI<br>RNI for P was >200% in 26% of patients<br>Median P intake: 162% of the RNI. 18/19 took >100% of the RNI for protein, which contributed to the high P intake<br>Despite use of infant soya formula or Ca supplemented soya milk and regular counselling, median intake of milk substitute was only 200 ml/day	The nutritional intake recorded prospectively using a 3-day dietary assessment based on estimated weights
Waisbren et al. 2012	8	Cross-sectional on the adult phenotype, with nutritional evaluation	33 CG patients, 17 male, 16 female<br>Age 18-59 yrs (mean 32.6±11.7 yrs; median 31 yrs)	Average intake (without supplementation) of 675 mg Ca, 3.8 mcg vitamin D, 1110 mg P, and 282 mg Mg (dietary records)<br>80% intakes below daily recommended intake (DRI) for Ca and 75% intake below the DRI for vitamin D.<br>Mean plasma 25OHD level: 27±11 ng/ml (reference range 32–100 ng/ml), with 80% below sufficient range<br>Two-thirds take Ca supplements and 38% vitamin D supplements, but irregularly	
Wiesmann et al. 1995	9	Experimental study to evaluate the effect of a raffinose- and stachyose-poor diet for a period of 14 days	6 CG patients, sex unknown<br>Age 6-24 yrs	Regular diet and the experimental diet low in stachyose and raffinose was low in P and Ca:<br>5/6: intake of Ca 20%-50% and of P 60%-70% of the daily recommended allowance	

Recommendation #8

First author, year	***#***	***Study design***	***Study population (number & age)***	***Results***	***Remarks***
Cangemi et al. 2012	1	Clinical validation of Gal-1-P measured with GC-MS	Gal-1-P determined:<br>58 CG patients<br>10-58 age 0-7 mnths<br>48/58 age 1-45 yrs<br>Measurements: 10/58 at diagnosis, the remainder were analysed only during follow-up	At diagnosis (age 23 days – 7 months), on a diet: 10.5 – 17.1 mg/dL<br>During follow-up on a galactose-restricted diet: ranging from the limit of quantification to 3.5 mg/dL<br>5 patients associated with poor compliance or incidental galactose intake: high Gal-1-P (5 mg/dL)<br>Gal-1-P decreased with age and reached a base level	
Hughes et al. 2009	2	Retrospective case series to compare outcomes in siblings	30 CG patients, 14 pairs of siblings, 2 patients with 2 siblings<br>Age 6 - 26 mnths	Mean Gal-1-P values:<br>Firstborns 178±µ87mol/L, second-born 171±51 µmol/L (difference not significant)<br><br>Mean galactitol values:<br>First-born 214±80 µmol/L, second-born 243±78 µmol/L (difference not significant)<br>Peak Gal-1-P values:<br>First-born 2586±2003 µmol/L, second-born 759±770 µmol/L<br>No simple correlation was noted when the mean Gal-1-P and galactitol values were plotted against IQ	

First author, year	**#**	***Study design***	***Study population (number & age)***	***Results***	***Remarks***
Hutchesson et al. 1999	3	Retrospective case series on RBC Gal-1-P and urinary galactitol concentrations	32 CG patients, studied over periods of up to 10.9 yrs (median 3.45 yrs)<br>Age 0-28 years<br><br>438 samples for RBC Gal-1-P<br>383 samples for urine galactitol	After start of galactose restriction: Gal-1-P concentrations fall rapidly from pre-treatment levels of 1000-3000 µmol/L RBC over a 3-month period.<br>Galactitol excretion even more rapid reduction from pre-treatment levels of 2660-11 300 µmol/mmol creatinine, mean levels falling 10-fold within 2 months<br>Concentrations of both Gal-1-P and galactitol then continue to fall exponentially with times to half-disappearance of 6.31 and 6.45 yrs, respectively, before stabilizing at mean levels of 104 µmol/L RBC for Gal-1-P and 193 µmol/mmol creat for urine galactitol at about 7-8 yrs age.<br>Galactitol and Gal-1-P excretion remained above the reference range in all patients (except for Gal-1-P in 1 patient)<br>Large intra-individual variability, persisting after correction for patients age, indicating that single measurements of Gal-1-P/galactitol are unlikely to be useful<br>Gal-1-P: biological variation within individuals is less than that between them, and that serial monitoring is more informative than comparison with population-derived reference data. The same appears true for urinary galactitol/creatinine ratios<br>Correlation between Gal-1-P and galactitol is strong in some, and extremely poor in others	

First author, year	***#***	***Study design***	***Study population (number & age)***	***Results***	***Remarks***
Pesce and Bodourian 1982	4	Cross-sectional study on galactose and Gal-1-P levels in correlation to clinical symptoms	Galactose and Gal-1-P levels in:<br>6 CG patients (under dietary treatment), 5 male, 1 female, age 2-15 yrs<br>1 CG patients (not under dietary treatment), age 3.5 months<br>12 individuals with below-normal GALT activity (not under dietary treatment), GALT-activity 7-17 U/g Hb<br>6 male, 6 female, age 4 months-32 yrs		**Due to a very heterogeneous sample of patients, with high risk of bias, it was decided not to include this article as evidence**
Schadewaldt et al. 2003	5	Cross-sectional study on quantitative assessment of the age dependence of endogenous galactose formation, with assessment of biochemical characteristics of subjects	51 CG patients, 28 male, 23 female, age 2-34 yrs<br>49 obligate heterozygous parents, 25 male, 24 female, age 25-71 yrs<br>215 healthy subjects, 100 male, 115 female, age 3-58 yrs	Postabsorptive G1P: range 2.36- 6.17 mg/dLRBC (91–238 µmol/LRBC) in patients versus <0.05 mg/dLRBC (<2 µmol/LRBC) in healthy subjects.<br>In plasma, galactose (2.9±0.4 lmol/L) and galactitol (11.0±0.9 lmol/L) concentrations were increased about 25-fold and 70-fold over control values, respectively, with a rather low interindividual variability<br>In urine, galactose, and galactitol content ranged from 3.3 to 9.5 and from 101 to 289 µmol/mmolcreat, respectively, and was about 10-fold and 100-fold higher, respectively, than the control values	Urine samples were collected in the postabsorptive state

First author, year	#	Study design	Study population (number & age)	Results	Remarks
**Waggoner et al. 1993**	6	Questionnaire among specialists about long-term complications	750 questionnaires distributed to over 40 specialists<br>371 questionnaires were returned from 29 contributors, patients 51% male, 49% female, age range 2 weeks – 37 yrs, mean 9.5 yrs	Gal-1-P levels from liquid blood samples were available for 177 cases. Gal-1-P levels (mean ± SD):<br>At diagnosis: 43.7± 43.8 (n=58)<br>0-1 mnth: 10.5± 7.9 (n=18)<br>1-6 mnts: 4.8± 3.0 (n=66)<br>6-24 mtns: 3.3± 2.0 (n=88)<br>2-12 yrs: 2.6± 1.4 (n=120)<br>>12 yrs: 2.5± 1.4 (n=50)<br>The mean levels of Gal-1-P were compared among those with and without specific complications (developmental delay, speech problems, ovarian function and growth), and except for lower hormone levels in the few women with normal ovarian function, there were no consistent relationships between Gal-1-P and other problems	

Recommendation #9: No literature available to support this recommendation

Recommendation #10: No literature available to support this recommendation

Recommendation #11: No literature available to support this recommendation

Recommendation #12

First author, year	#	*Study design*	*Study population (number & age)*	*Results*	*Remarks*
Antshel et al. 2004	1	Cross-sectional study on neuropsychological and behavioral profile	25 CG patients, 15 male, 10 female<br>Age 8-14 yrs (mean 10 yrs, 9 months, SD 2 yrs 2 months)<br><br>20 controls, 11 male and 9 female<br>Age 8-13 yrs (mean 10 yrs 8 months, SD 1 year 1 month)	All patients homozygous for Q188R.<br>Patients full IQ mean 84.3 SD 8.3, controls 99.0 SD 15.5<br>Mean IQ is lower in CG than in control subjects, with CG patients functioning generally within the low average IQ range	
Badawi et al. 1996	2	Retrospective case series on screening and outcome	55 CG patients, 7 DG patients<br>Follow-up with cognitive evaluation in:<br>32 patients, sex unknown, age 2 mnths-20 yrs	5/32: IQ range of 63-74, 1/32: IQ of 84<br>18/32: IQ range of 91 -119	
Coss et al. 2013	3	Retrospective case series on Irish birth incidence of CG and long-term clinical outcomes	(130 CG patients)<br>85 patients screened for cognitive outcome: 48 male, 37 female<br>Age average 18.8 yrs (median 18.5 yrs), age range 6-39 yrs	No significant difference in IQ scores between older and younger patients<br>Patients 6-12 years (n=25): 100% IQ ≤89<br>Patients >12 years (n=60): 73.3 % IQ ≤89<br>30.6 % of patients ≥6 years: IQ range <70<br>25.9% of patients ≥6 years: IQ range 70-79<br>24.7 % of patients ≥6 years: IQ range 80-89<br>Majority of patients IQ ≤79 (56.5 %)<br>11.8 % in average and 7.1 % in high-average ranges	Both Travellers and non-Travellers included.<br>Cognitive outcomes were not compared between the two groups as there may be unintentional bias in IQ scores between the Traveller and non-Traveller groups
Donnell et al. 1961	4	Retrospective case series on growth and development	16 CG patients, 9 male, 7 female<br>Age 2-12 yrs	IQ/DQ: average 79, median 84 (range 11-108)<br>8/16 ≤ 85, 4/16 85-100, >100 3/16	

First author, year	#	Study design	Study population (number & age)	Results	Remarks
Doyle et al. 2010	5	Cross-sectional study on the neuropsychological profile	28 CG patients, 20 female, 8 male<br>Age 15-53 yrs (mean 29.3, SD=10.3)	Verbal IQ mean 88.9, SD 16.8<br>Performance IQ mean 85.1, SD 12.9<br>Mean scores mostly in the low average range<br>Considerable variability for each of the cognitive variables, with some individuals in the average range (25th–74th percentile) or above (75th-90th percentile, none >91th percentile) and others in the borderline (2nd–8th percentile) or extremely low (below 2nd percentile) range	No total IQ scores reported
Fishler et al. 1966	6	Retrospective case series on intellectual and personality development	34 CG patients, 18 male, 16 female<br>Group I: n=7, age 12-17 yrs<br>Group II: n=11, age 5-9 yrs<br>Group III: n=16, age 2 months-5 yrs	Note: no ages at testing available<br>Group I: IQ initial 64-94, stable over 8 yrs, last IQ 66-99<br>Group II: IQ initial 60-100, last IQ 56-111<br>Group III: DQ initial 94-121, last DQ 57-112. Some show gains and some losses in DQ in time. This probably reflects instability of early infant test scores. All but one score fall in normal range<br><br>Overall: 29/34 tested patients have normal IQ >90. Mean DQ/IQ score for males (n=18) = 95.8, mean DQ/IQ score for females (n=16) = 91	Not clear what exact age at testing is
Fishler et al. 1972	7	Retrospective case series on developmental aspects	45 CG patients, 22 male, 23 female<br>Group I: n=8, 1 month-5.5 yrs<br>Group II: n=30, 6-15 yrs<br>Group III: n=7, age >16 years	Group I: DQ/IQ range: 78-122, mean 105<br>Group II: IQ range: 40-117, mean 87<br>Group III: IQ range: 78-104, mean 92<br>Mean IQ entire sample 45 patients: 91<br>Younger sibs with CG with direct start of dietary treatment after birth: IQ 95, no significant difference with older sibs	Additional data to report of 1966

First author, year	#	Study design	Study population (number & age)	Results	Remarks
Fishler et al. 1980	8	Retrospective case series on developmental aspects	60 CG patients, 29 male, 31 female<br>Group I: n=13, age 5 mnths-1.5 yrs<br>Group II: n=25, age 6-17<br>Group III: n=22, age 17-29 yrs<br>11 pairs of CG siblings: index case and younger sibling	Group I: DQ/IQ range: 70-125, mean 102 SD 12.8<br>Group II: IQ 50-117, mean 91 SD 17.5<br>Group III: IQ 72-119, mean 94 SD 18.2<br>Mean IQ entire sample 60 patients: 95<br>11 pairs of sibs: index case IQ mean 97, SD 17.6, younger sibling mean IQ 96, SD 15.1, no significant difference	Additional data to report of 1966 and 1972
Hansen et al. 1996	9	Cross-sectional study on neurodevelopmental and linguistic performance	16 CG patients, assessment of cognitive function with IQ score in 6 patients, 5 male, 1 female<br>Age 3 yrs-19 yrs 2mnths	IQ range 60-117	
Hoffmann et al. 2011	10	Cross-sectional study on speech and cognitive performance	32 CG patients, male 20, female 12<br>Mean age 21.2±7.2 (range: 9.9–37.4) yrs<br>7 girls and 12 boys <18 yrs	Mean full scale intelligence quotient (FSIQ): mean 76.2±14.8, range 52–109<br>Mean performance IQ: 74.4±16.3 (range 41–111)<br>Mean verbal IQ: 82.2±13.5 (range 61–105).<br>IQ scores don't differ significantly between patients <18 years and adults or between females and males<br>Only 9 patients (28.1%) had an FSIQ of ≥85 (71.9% IQ <85)	36 of these patients had taken part in a previous investigation of the long-term outcome of galactosemia (Schweitzer et al. 1993)
Kaufman et al. 1994	11	Retrospective data on correlation of cognitive, neurologic, and ovarian outcome with the Q188R mutation	41 CG patients<br>24 patients homogyzous for Q188R, mean age 19.5 yrs, SD 12.1<br>12 heterozygous for Q188R/other (not otherwise described), mean age 15.2 yrs, SD 9.1<br>5 patients without Q188R, mean age 11.2 yrs, SD 7.4	Broad Cognitive scores:<br>Homozygous patients: 75, SD 16, range 25-95<br>Heterozygous patients: 67 SD 25, range 13-100<br>No Q188R: 88, SD 21, range 61 to 115<br>No statistically significant differences were observed among the groups	

First author, year	#	Study design	Study population (number & age)	Results	Remarks
Kaufman et al. 1995	12	Cross-sectional study on cognitive functioning, neurologic status and brain imaging	45 CG patients, 23 male, 22 female<br>Age 4.0-39 yrs<br><br>Broad cognitive scores in 40 patients,<br>19 aged 4-18 yrs, 21 aged 18-34 yrs	Score 4-18 yrs: 73.0 SD 20.9 (range 25-110)<br>Score 18-34 yrs: 71.9 SD 18.6 (range 13-95)<br>7/40 scored in range 90-115, 8/40 in range 80-89, 11/40 in range 70-79 and 14/40 in range < 70<br>No significant difference between the sexes on the mean broad cognitive score: 73.1 for males and 71.6 females	
Komrower et al. 1970	13	Retrospective case series on physical and mental health	60 CG patients, 22 male, 38 female<br>Age 2 yrs 2 mths-17 yrs 7 mths, mean 8 years 5 months	All patients: mean IQ 80 (range 30-118)<br>Male: mean IQ 89 (range 54-118)<br>Female: mean IQ 73 (range 30-111)<br>The difference between the mean IQ of boys and girls is significant<br>0-5 years (mean age 3 yrs 4 mths), n=21: mean IQ 90, range 61-118<br>5-10 years (mean age 8 yrs 3 mths), n=25: mean IQ 79, range 30-112<br>10-15 years (mean age 13 yrs 7 mths), n=14: mean IQ 70, range 45-107	
Lee et al. 1972	14		Same data used as in Komrower et al. 1970		

First author, year	#	Study design	Study population (number & age)	Results	Remarks
Manis et al. 1997	15	Non-RCT on effect of uridine treatment on long-term neurocognitive function	35 CG patients, 18 male, 17 females<br>Age 1 week - 16.2 yrs<br><br>Uridine treatment: 29 patients, treatment with oral uridine 150 mg/kg per day for a period ranging from 1.0 to 5.9 yrs (mean 3.1 yrs). 7 patients <14 mths, 22 patients 3.5-14.1 yrs (mean 7.1 yrs)<br><br>Comparison: 6 patients only dietary restriction, age 6.3-16.2 years (mean 13.4 yrs) followed for a period ranging from 2 to 5 yrs (mean 3.9 yrs)	Patients 3.5-14.1 years:<br>Uridine (n=22): initial 77.6 (SD 17.8), final 82.7 (SD 18.3)<br>Comparison (n=6): initial 86.2 (SD 13.7), final 91.3 (SD 9.8)<br>Patients <14 months<br>After uridine (n=7): 89.6 (SD 15.4)<br>Baseline cohort (n=9): 84.9 (SD 11.7)	
Rasmussen et al. 1996	16		Same data used as Hansen et al. 1996		
Rubio-Agusti et al. 2013	17	Cross-sectional study on frequency and phenotype of motor dysfunction. Information regarding cognitive assessment was collected from the medical chart	Movement disorders and IQ in:<br>47 CG patients, 18 male, 29 female<br>Age 20-38 yrs, median 26 yrs	15/47 (32%): history of cognitive problems. All had learning difficulties, and 9 patients had a performance and/or verbal IQ≤80	

First author, year	#	Study design	Study population (number & age)	Results	Remarks
Schadewaldt et al. 2010	18	Cross-sectional study on intra-individual development of cognitive function with age	23 CG patients, previously evaluated by Schweitzer et al. 1993 (study 1), 12 male, 11 female<br><br>Age study 1: mean age 10.8 yrs SD 5.2<br>Age study 2: 25.7 yrs SD 5.3	Total IQ study 1: 78 SD 14 (73 [58 –106])<br>Total IQ study 2: 73 SD 15 (70 [55–109])<br>PIQ study 1: 73 SD 17 (70 [45–103])<br>PIQ study 2: 74 SD 17 (68 [47–111])<br>VIQ study 1: 86 SD 11 (84 [69 –113])<br>VIQ study 2: 77 SD13 (76 [57–105])<br>Mean TIQ and PIQ scores of both tests were not significantly different. The mean VIQ score showed a variable but statistically significant decline	
Shield et al. 2000	19	Cross-sectional study of cognitive outcome and its relationship to genotype	34 CG patients, sex unknown<br>Median age 6.4 yrs (range 4.0–8.6 yrs)	Mean IQ: 79 (range = 49–116).<br>In 32 cases: mean performance score was 79 (range = 49–121) and mean verbal score 82 (range = 52–121).<br>10/34 IQ > 85. 21/34 IQ 56–85. 3/34 IQ <56 (71% <IQ 85)	
Schweitzer et al. 1993	20	Specialist questionnaire on long-term outcome	Long-term outcome in 134 CG patients, complete clinical evaluations in 83 patients of which 5 DG patients (49 male, 34 female; age 9 months-33 yrs, mean 9.5 + 7.1 yrs) and retrospective evaluations only in 31 patients (15 male, 16 female; age 9 months-27 yrs, mean 10.2± 8.8 yrs)	DQ/IQ < 85 in 4/34 patients <6 years (12%), in 10/18 patients between 7 and 12 yrs (56%) and in 20/24 patients >12 yrs of age (83%).<br>34/76 DQ/IQ < 85 (45%).	

First author, year	#	Study design	Study population (number & age)	Results	Remarks
Waggoner et al. 1990	21	Specialist questionnaire on long-term prognosis	371 questionnaires on long-term outcome, 350 cases included<br>178 male, 172 female<br>Age 2 weeks-37 yrs, mean 9.5 yrs<br>Scores on developmental or intelligence tests in 184 cases<br><br>Various developmental and intelligence tests had been given at different ages, therefore DQ/IQ scores grouped by age	< 1 years: n=35, DQ 109 SD 11, range 89-136<br>1-2 years: n=71, DQ 97 SD 17, range 45 123<br>3-5 years: n=85, IQ 92 SD 19, range 50-138<br>6-9 years: n=88, IQ 87 SD 19, range 39-142<br>10-16 years: n=67, IQ 80 SD 17, 26-115<br><br>Mean IQ scores of females as a group significantly lower than males at 10-16 yrs and >16 yrs. No consistent declines in the scores of individuals who had been tested repeatedly with the same IQ test. A greater incidence of developmental delay among cases who were not treated until after 2 months of age. However, IQ scores were not highly correlated with the age when treatment began	
Waggoner et al. 1993	22		Data based on previous dataset (Waggoner et al. 1990)		
Waisbren et al. 1983	23	Cross-sectional study on intelligence and speech and language development	8 CG patients, 2 male<br>Age 3 yrs 7 mnth-11 yrs 7 mnths	FIQ mean 97 SD 13, range 76-122<br>PIQ mean 102 SD 11, range 88-121<br>VIQ mean 92 SD 14, 68-118<br>7/8 verbal score less than the performance score.	
Waisbren et al. 2012	24	Cross-sectional study to assess the adult phenotype	33 CG patients, 17 male, 16 female<br>Age 18 to 59 years (mean age 32.6±11.7 yrs; median age 31 yrs)	IQ 55–122, mean of 88±20<br>13/33 (39%) IQ ≤85, 8/33 (24%) IQ ≤70 .	

First author, year	#	Study design	Study population (number & age)	Results	Remarks
Widhalm et al. 2002	25	Non-RCT on effect on urine treatment on indices of the basic cognitive processes underlying intellectual skills	12 CG patients, 7 male, 5 female<br>Mean age 11.2 SD 2.2 yrs<br><br>29 healthy, age matched controls, 18 male and 11 female<br>Mean age 12.6 SD 0.6 yrs	Uridine was administered orally in a dose of 150 mg/kg/day for 540 SD 3 days. Only 7 CG patients completed entire program<br>All patients homozygous for Q188R<br>IQ controls: 110.1 SD 9.1<br>IQ patients: 80.9 SD 17. This difference is significant<br><br>7 CG patients before and after 18 months of uridine treatment: baseline IQ 82.9 SD 20.9 and post treatment IQ 81.0 SD 21.0. Not statistically different	

Recommendation # 13: No literature available to support this recommendation

Recommendation #14

First author, year	#	*Study design*	*Study population (number & age)*	*Results*	*Remarks*
Antshel et al. 2004	1	Cross-sectional study on neuropsychological and behavioural profile	25 CG patients, 15 male, 10 female<br>Age 8-14 yrs (mean 10 yrs, 9 mnths, SD 2 years 2 mnths)<br><br>Twenty controls, 11 male and 9 female<br>Age 8-13 yrs (mean 10 yrs 8 mnths, SD 1 year 1 mnth)	CG patients exhibit less well-developed executive functions<br>On the WCST, CG patients completed a fewer number of categories, and had a higher percentage of perseverative errors. Compared with parents of control participants, parents of participants with galactosemia reported on the BRIEF their children have more difficulty getting started on projects and using their working memory	Wisconsin Card Sorting Test (WCST)<br>Behavior Rating Inventory of Executive Function (BRIEF)
Doyle et al. 2010	2	Cross-sectional study on the neuropsychological profile	28 CG patients, 20 female, 8 male<br>Age 15-53 yrs (mean 29.3 yrs SD 10.3)	Executive functioning in CG patients, measured by working memory indices and specific executive functioning measures (Hayling and Brixton Tests), are low average and borderline, respectively.<br>Mean scores of executive functions are below the average	
Waisbren et al. 2012	3	Cross-sectional study to assess the adult phenotype	33 CG patients, 17 male, 16 female<br>Age 18-59 yrs, mean 32.6±11.7 yrs, median 31 yrs	BRIEF:<br>5/33 (15%): deficits in executive functioning	Behavior Rating Inventory of Executive Function (BRIEF)
Widhalm et al. 2002	4	Non-RCT on effect on urine treatment on indices of the basic cognitive processes underlying intellectual skills	12 CG patients, 7 male, 5 female, mean age 11.2 SD 2.2 yrs<br>29 healthy, age matched children, 18 male and 11 female, mean age 12.6 yrs SD 0.6	CG patients show a markedly comprised development with regard to attention and information processing, specifically deficits in central processing stages (suggesting a reduced processing capacity) and a reduced ability to sustain attention	

Recommendation #15

First author, year	#	*Study design*	*Study population (number & age)*	*Results*	*Remarks*
Badawi et al. 1996	1	Retrospective case series on screening and outcome	32 CG patients, sex unknown, age unknown	7/32: speech complaints<br>3/7: requiring formal therapy	No method of assessment described, speech complaints not otherwise described
Coss et al. 2013	2	Retrospective case series on Irish birth incidence of CG and long-term clinical outcomes	117 CG patients, 54 Travelers population, 63 non-Travelers, age >2.5 yrs	Speech and language problems (including verbal dyspraxia, speech delay or difficulties, no speech or severe speech delays or some verbal comprehension problems):<br>28/54 Travelers (51.9%)<br>30/63 non-Travelers (47.6%)	All children 2.5 yrs or older with speech and language disorders either noted by the parents or medical personnel were assessed by clinical speech and language therapists. Not mentioned what assessments
Hansen et al. 1996	3	Cross-sectional study on neurodevelopmental and linguistic performance	16 CG patients, 8 patients assessed for speech problems, 5 male and 1 female<br>Age 3-19 yrs	3/6 mild-moderate verbal dyspraxia (not otherwise described)<br>Speech and language development affected in 5/8	A psycholinguistic test (Reynell or ITPA) was administered to the 6 oldest patients. All had comprehensive language development evaluation performed by a speech therapist
Hoffmann et al. 2011	4	Cross-sectional study on speech and cognitive performance	32 CG patients, 20 male, 12 female (including 7 girls and 12 boys <18 yrs)<br>Mean age 21.2±7.2, range 9.9–37.4 yrs	The Hierarchische Wortlisten word-repetition test<br>84.4% of the patients passed the word-repetition test with errors indicating some degree of speech impairment, which is present already in childhood and persists into adulthood	

First author, year	#	Study design	Study population (number & age)	Results	Remarks
Hughes et al. 2009	5	Retrospective case series on outcomes of siblings	30 CG patients, 14 sibling pairs, 2 families had a 3rd sibling, 19 male, 11 female<br>Age 6-26 mnths<br>Speech and language test in 13 index cases, 13 younger sibs	Abnormal speech and language:<br>Index case: 9/13 (69%)<br>Younger sibling: 11/13 (85%)<br>Total 77%	The presence or absence of speech and language problems was documented. Not clear how this was assessed
Lee et al. 1972	6	Retrospective case series on intellectual and emotional status	60 CG patients, 22 male, 38 female<br>Age 2 yrs 2 mths-17 yrs 7 mths, mean 8 yrs 5 mnths	15/60 (25%) speech impediments	No method of assessment described, speech impediment not otherwise described
Milankovics et al. 2010	7		23 CG patients, sex unknown<br>Age >4 yrs<br>5 patients with residual enzyme activity (level unknown), sex unknown<br>Age >4 yrs		**High risk of bias due to multiple pathology (cardiac disease, dysmorphic features) in the reported patients. No method of assessment described, speech problems not otherwise described. Therefore not included as evidence**
Nelson et al. 1991	8	Cross-sectional study to assess speech characteristics	24 CG patients, 11 male, 13 female<br>Age 2-23 yrs	13/24 (54%) characteristics of verbal dyspraxia (1 mild, 6 moderate, 6 severe)<br>2/24 (8%) articulation disorders without verbal dyspraxia<br>9/24 (38%) normal articulation<br>IQ scores of patients with dyspraxia (IQ 78 SD 18) are significantly lower than those of nondyspraxic patients (IQ 99 SD 13)	Patients evaluated and classified on the basis of accepted (but adapted) speech assessment protocols.<br>Severity of apraxia defined

First author, year	#	Study design	Study population (number & age)	Results	Remarks
**Karadag et al. 2013**	9	Retrospective case series of features and outcome of CG patients diagnosed in newborn period	22 CG patients, 18 male, 4 female<br>Age unknown<br>Follow-up after admission PICU, follow-up 1-6 yrs	Speech deficits 4/22 (18%)<br>Verbal dyspraxia 4/22 (18%)	No method of assessment described, speech deficits not otherwise described
**Potter et al. 2008**	10	Cross-sectional study to assess receptive and expressive language Status and assemble findings on associations among cognition	33 patients, 22 male 11 female<br>Age 4-16 yrs	All patients in the sample started speech-language therapy prior to age 4. At time of testing, 76% of the 4-16 yrs patients still received therapy<br>56% of patients with average cognition and 88% patients with borderline-low cognition have co-occurring speech and language disorders. Children with CG have a 4- to 6-fold greater relative risk for co-occurring language disorders. CG patients with the Q188R/Q/188R genotype are at greater risk than participants with Q188R/other genotypes for lower IQ and language impairment<br>Language impairments appear to arise in the absence of familial risk factors and to persist even with early speech-language intervention	Listening Comprehension Scale (receptive language measure) and Oral Expression Scale (expressive language measure) from the Oral and Written Language Scales

First author, year	#	Study design	Study population (number & age)	Results	Remarks
Potter et al. 2011	11	Cross-sectional study to examine phonatory function and voice quality	33 CG patients, age 4-16 yrs<br><br>130 controls (typically developing children), 5 females and 5 males<br>Age 4-16 yrs	58% of children with CG and speech disorders have reduced respiratory-phonatory support for speech, and 33% have disturbed vocal quality to laryngeal insufficiency<br>Vocal tremors in 9%<br>Maximum phonation times in CG patients with or without speech disorders are reduced (32% and 62%) compared to controls, not correlated to expressive language, receptive language, or IQ standard scores	Speech and language were assessed through a battery of formal and informal tests (Potter et al. 2008; Shriberg et al., 2010). Expressive language with the Oral Expression subtest and receptive language with the Listening Comprehension subtest of the Oral and Written Language Scales (OWLS; Carrow-Woolfolk 1995)
Potter et al. 2013	12	Cross-sectional study to examine motor and speech disorders	32 CG patients, 21 male, 11 female<br>Age 4-16 years<br>130 control children, 5 males and 5 females from each 6-month age group from 4–16 years of age	CG patients have weaker tongue strength compared to controls. 66% of CG patients (children) with speech disorders have co-occurring coordination disorders and children with CG and CAS (childhood apraxia of speech) or dysarthria have poorer balance and dexterity. The number of days on milk during the neonatal period is associated with worse speech outcomes in males, not females, with CG	Tongue strength were assessed using the Iowa Oral Performance Instrument (IOPI)<br>The Madison Speech Assessment Protocol and the Speech Disorders Classification System (Shriberg et al. 2010)
Powell et al. 2009	13	Retrospective case series on long-term speech and developmental issues	75 DG patients, age 3-10 yrs	Higher percentage of DG patients receiving special education services compared with the general population of children for both 3 to 10 years and 8 yrs. Results not statistically significant	

First author, year	#	Study design	Study population (number & age)	Results	Remarks
Rasmussen et al. 1996	14	Retrospective case series on learning disabilities and language pathology	8 CG patients, 5 male, 3 female<br>Age 9 months-19.1 yrs<br><br>6 patients speech evaluation (2 were too young), age 3-19.1 yrs	Developmental dyspraxia of speech in 3/6 patients (1 mild, 2 moderate)<br><br>6/8 patients scored below chronological age at psycholinguistic age	Verbal dyspraxia assessed with checklist according to Pollock et al. 1991.<br>Language evaluation with age appropriate test: Griffith's Developmental Scale (sub-scale hearing and speech), Reynell Developmental Language Scales, ITPA
Rubio-Agusti et al. 2013	15	Cross-sectional study on frequency and phenotype of motor dysfunction. Information regarding cognitive assessment was collected from the medical chart	Movement disorders and outcome in:<br>47 CG patients, 18 male, 29 female<br>Age 20-38 yrs, median 26 yrs	16/47 (34%): speech problems including delay in speech development and speech apraxia.	
Shriberg et al. 2011	16	Cross-sectional study to assess childhood apraxia of speech	33 CG patients with prior or persistent speech sound disorder, 21 male, 11 female (1 unknown), 4-16 years<br><br>11 patients excluded from initial analyses because no prior or persistent speech sound disorder	8/33 (24%) met contemporary diagnostic criteria for childhood apraxia of speech. 1 of these 8 had dysarthria, excluded from other analyses.	Preliminary version of the Madison Speech Assessment Protocol (MSAP)<br>Expanded version of the MSAP

First author, year	#	Study design	Study population (number & age)	Results	Remarks
Schweitzer et al. 1993	17	Specialist questionnaire on long-term outcome	66 CG patients, age >3 years, examined<br><br>31 CG patients in history group (no assessments)	Speech abnormalities were observed in 43 out of 66 examined probands over 3 years (65%): 15 had isolated dyslalia, 9 multiple dyslalia, 9 dyspraxia, 7 dysgrammatism and one stuttered<br>Speech therapy in 21/66<br><br>12/31 patients of the history group had speech abnormalities, 5/12 received speech therapy. The status of speech development was not known in the remaining 19 cases	Speech documentation by tape recording
Timmers et al. 2012	18	Cross-sectional study to assess event related potentials of sentence production	22 CG patients, 7 male, 15 female<br>Age mean 14.9 years (SD 2.2 years, range 10.8–19.1 years)<br><br>21 controls, 7 male, 14 female<br>Age mean 14.2 years (SD 1.8 years, range 11.4–17.0 years).	Patients with CG show difficulties in language production tasks, both behaviourally (less accurate and slower) and in their event-related potentials (ERP), compared to healthy controls. The ERP differences continue throughout the consecutive linguistic preparation phases, which indicates an affected lexical access and impaired syntactic planning	
Waggoner et al. 1990	19	Specialist questionnaire on long-term prognosis	371 questionnaires<br>243 cases, age >3 years for speech evaluation	Problems with speech were reported in 56% (136/243)<br>Not known how many cases had had formal speech evaluations.<br>92% of the cases with speech problems described as having delayed vocabulary and 90% had articulation problems: 34 cases were said to have 'disordered' articulation, which we have defined as verbal dyspraxia.<br>At least 65% of the cases with speech problems had received speech therapy. Cases with speech problems had significantly lower DQ and IQ scores at all ages than those with normal speech	Questionnaire, not known how many cases had had formal speech evaluations.

First author, year	#	Study design	Study population (number & age)	Results	Remarks
Waggoner et al. 1993	20		Data based on previous dataset (Waggoner et al. 1990)		
Waisbren et al. 1983	21	Cross-sectional study on intelligence and speech and language development	2 female, 6 male CG patients<br>Age 3.6-11.6 years	* Deficit = 1 SD below mean or one year below chronologic age<br>7/8 had speech and language deficits in at least one category, most marked in the area of expressive language<br>5/8 Speech production (articulation) deficits<br>1/8 normal scores on all performed tests<br>4/8 receptive language deficit (comprehension)	Assessment of speech and language functioning with a variety of tests of language comprehension, memory, expressive language, and articulation
Waisbren et al. 2012	22	Cross-sectional study to assess the adult phenotype	33 patients, 17 males and 16 females<br>Age 18 to 59 years (mean age=32.6±11.7 years; median age=31 years)	Deficits in motor speech function is defined by scores of 1 SD or more below the mean of healthy controls on standardized measures<br>Reduced tongue strength (Iowa Oral Performance Instrument) in 24/33 (73%) .<br>Breath support for speech (measured by decreased phonation duration) decreased in 21/33 (64%)<br>Articulation proficiency reduced in 4/33 (12%)<br>Dysarthria in 8/33 (24%).<br>Apraxia of speech (affecting the planning and programming of speech movements) in 3/33 (9%).<br>Receptive vocabulary reduced in 14/33 (42%).<br>3 men (and no women): mild-moderate high frequency hearing loss (two unilateral; one bilateral)	Tongue strength (Stierwalt and Youmans 2007), Maximum phonation duration (Kent 1994), Goldman-Fristoe Test of Articulation-2 (Goldman and Fristoe 2000), Dysarthria and apraxia of speech (Duffy 2005), Peabody Picture Vocabulary Test-IV (Dunn and Dunn 2007)

First author, year	***#***	***Study design***	***Study population (number & age)***	***Results***	***Remarks***
Webb et al. 2003	23	Cross-sectional study/Case series to assess potential biochemical risk indicators for verbal dyspraxia	24 CG patients speech evaluations, 17 male, 7 female<br>Age mean 9 years 2 months SD 6 years 2 months (range 2.5–30 years)<br><br>(From total study population of 42 CG patients, age >2.5 years, 13 healthy controls)	15/24 CG patients have dyspraxia of speech (62.5%)<br>Determining total body oxidation of 13C-D-galactose to 13CO2 (CUMPCD, breath test):<br>The average CUMPCD value of patients with dyspraxia: 2.84 +- 5.76%. This value is significantly lower than the CUMPCD of patients without dyspraxia: 11.51 +- 7.67%.<br>CUMPCD values <5% are strongly and significantly associated with risk for dyspraxic outcome after controlling for confounders [adjusted OR=21.1 (1.68, 265).	The Apraxia Profile<br>Three patients were evaluated for dyspraxia through the Atlanta Area School System

Recommendation #16: No literature available to support this recommendation

Recommendation #17: No literature available to support this recommendation

Recommendation #18 (numbers 1, 3, 6, 7, 8, 9, 10, 12, 13, 14, 15, 16, 17)

Recommendation #19 (numbers 2, 4, 5, 14)

First author, year	***#***	***Study design***	***Study population (number & age)***	***Results***	***Remarks***
Coss et al. 2013	1	Retrospective case series on Irish birth incidence of CG and long-term clinical outcomes	130 CG patients, 74 male, 50 female, 6 unknown<br>63 travellers, 67 non-travellers<br>Age 0.5-45 yrs	Ataxia (in patients >2.5 yrs): 7/130 (2/54 Travellers, 5/63 non-Travellers)	Some of these subjects are included in Hughes et al. 2009
Donnell et al. 1961	2	Retrospective case series on growth and development	15 CG patients, 8 male, 7 female<br>Observations from 18 mnths 12 yrs, mean 5 yrs 9 mnths	0/15: specific neurological abnormalities<br>EEG performed in 14/15 subjects<br>5/14: abnormal EEG<br>4/5 diffusely abnormal<br>1/5: spike-wave focus in the right occipital area<br>EEG findings could not be correlated to intellectual capacity of the individual children	

First author, year	*#*	*Study design*	*Study population (number & age)*	*Results*	*Remarks*
Dubroff et al. 2008	3	Cross-sectional study to assess cerebral glucose metabolism with FDG-PET	5 CG patients, 3 male, 2 female<br>Age 20-40 yrs, mean age 2 yrs<br>8 healthy controls, age 22-52 yrs, mean age 35 yrs	Neurology exam<br>5/5: tremor (4 'fine'), 1/5:cerebellar ataxia, 2/5 dystonic posturing / movements, 1/5 dysmetria, 1/5: abnormal gait<br>FDG-PET:<br>CG patients have significant decreases in the superior temporal lobes, temporal poles (cognitive impairment), orbital frontal lobes, midparietal regions (deficits in<br>spatial processing), cerebellum (cerebellar functions like cerebellar ataxia and tremors), and primary visual cortices. Also a significant metabolic increase in the anterior cingulate gyrus (movement and attention abnormalities). Statistical parametric mapping analysis: similar decreases observed in the cerebellum, the sensorimotor areas, and the left caudate nucleus. Relative increases in cerebral metabolism in the basal ganglia (tremor and motor findings). Non-specific areas of decreased metabolism in the white matter regions (cognitive dysfunction)	
Fishler et al. 1972	4	Retrospective case series on intellectual and personality development	45 CG patients, 22 male, 23 female<br>Group I: n=8, age 0-5 yrs<br>Group II: n=30, age 6-15 years<br>Group III: n=7, age 16-23 yrs	EEG results<br>Group I: 12% abnormal, 37% normal<br>Group II: 40% abnormal, 60% normal<br>Group III: 43% abnormal, 57% normal<br>Total sample: 45% normal, 55% abnormal	No definition of normal or abnormal EEG

First author, year	#	Study design	Study population (number & age)	Results	Remarks
Fishler et al. 1980	5	Retrospective case series on developmental aspects	60 CG patients, 29 male, 31 female<br>Group I: n=13, age 5 mnths-1.5 yrs<br>Group II: n=25, age 6-17 yrs<br>Group III: n=22, age 17-29 yrs<br>11 pairs of CG siblings: index case and younger sibling	EEG results<br>Group I: 1/5 abnormal<br>Group II: 12/24 abnormal<br>Group III: 5/22 abnormal	
Hughes et al. 2009	6	Retrospective case series to assess outcomes in siblings with CG, including neurologic and neuroimaging findings	30 CG patients from 14 families, 19 male, 11 female<br>Age 6 mnths-26 yrs	7/30 (23%): abnormal neurologic examination findings, including ataxia, marked tremor, and abnormal gross motor skills; 23/30 (77%) had normal examinations.<br>No major differences in outcomes between the first-born and second-born siblings. No difference in neurology between those having <20mg/day vs. >20mg/day galactose intake	
Karadag et al. 2013	7	Retrospective case series of features and outcome of CG patients diagnosed in newborn period	22 CG patients, 18 male, 4 female<br>Patiënts followed for 6 yrs (mean 3 yrs)	0/22 developed ataxia or dysmetria	
Kaufman et al. 1994	8	Retrospective data on correlation of cognitive, neurologic, and ovarian outcome with the Q188R mutation	67 CG patients, 32 male, 35 female<br>Age unknown	12/67: presence of tremor, ataxia, and dysmetria<br>Abnormal neurology not related to Q188R mutation	
Kaufman et al. 1995	9	Cross-sectional study on cognitive functioning, neurologic status and brain imaging	45 CG patients, 23 male, 22 female<br>Age 4-39 yrs	12/45: tremor, ataxia and dysmetria. No correlation between these neurologic findings and cognitive outcome.	Patients probably also reported in Kaufman et al. 1994

First author, year	***#***	***Study design***	***Study population (number & age)***	***Results***	***Remarks***
Krabbi et al. 2011	10	Retrospective case series on long-term complications in patients with a less restricted lactose-free diet	5 CG patients, 1 male, 4 female<br>Age 7-14 yrs	1/5: Ataxia, spastic syndrome, and stereotypic movements<br>1/5: Mild cognitive deficit and epilepsy<br>3/5: no neurological findings	
Milankovics et al. 2010	11		23 CG patients, sex unknown<br>Age >4 yrs<br>5 patients with residual enzyme activity sex unknown<br>Age >4 yrs		**No method of assessment described, motor abnormalities not otherwise defined. High risk of bias due to multiple pathology (cardiac disease, dysmorphic features) in the reported patients. Not included as evidence**
Nelson et al. 1992	12	Retrospective case series to assess magnetic resonance imaging appearance of the brain	63 CG patients and 4 variant patients, 31 male, 36 female<br>Age 1 mnth-42 yrs, median 10 yrs	10/63: ataxia	Definition variant: measurable but low RBC GALT activity confirmed with a sensitive radioactive multipoint assay. All 4 variant patients have <1% of normal GALT activity

First author, year	#	Study design	Study population (number & age)	Results	Remarks
Rubio-Agusti et al. 2013	13	Cross-sectional study on frequency and phenotype of motor dysfunction	47 CG patients, 18 male, 29 female<br>Age 20-38 yrs, median 26 yrs	31/47 (66%): motor dysfunction detected on examination, only 13/31 had symptoms<br>23/47 49%): tremor. All bilateral arm action tremor, 2 patients rest tremor, 4 patients head tremor<br>23/47 (49%): dystonic features mostly involving upper half of the body, generalized in 4 patients, segmental in 10, focal in 5, multifocal in 4<br>Dystonic features and tremor often combined (16 patients)<br>8/13 symptomatic patients: progressive worsening of symptoms (reported during interview)<br>6/47: cerebellar signs (6 ataxic gait, 4 limb dysmetria, 3 cerebellar dysarthria)<br>4/47: pyramidal signs (all brisk tendon refex and clonus, 3 extensor plantar response, 3 spastic paraparesis, 1 pseudobulbar signs)<br>4/47: eye movement abnormalities<br>3/47 (7%) history of epilepsy presenting in teenage yrs	
Schweitzer et al. 1993	14	Specialist questionnaire on long-term outcome	134 CG patients, complete clinical evaluations in 83 patients of which 5 DG patients (49 male, 34 female; age 9 months-33 yrs, mean 9.5 + 7.1 yrs)<br>Retrospective evaluations only in 31 patients (15 male, 16 female; age 9 months-27 yrs, mean 10.2 + 8.8 yrs)	54/83 (49/78 CG patients and 5/5 DG patients): actual neurological examination normal. Severe clumsiness present in 12 cases, 11 had intentional tremor, 3 had mild ataxia and 3 severe ataxia including<br>1 patient had familial epilepsy with grand real seizures<br>No correlation between neurological disturbances and onset of therapy<br>Among 31 patients of the history group, 20 gave no information concerning neurological status.<br>7/11: normal neurological development<br>3/11: epilepsy	

First author, year	#	Study design	Study population (number & age)	Results	Remarks
Viggiano et al. 2015	15	Retrospective case series on mutational analysis, residual enzyme activity and long-term clinical outcome in patients identified through NBS in northeastern Italy	14 CG patients, 8 male, 6 female<br>Age last follow-up 1-34 yrs<br><br>2 DG patients, 1 male, 1 female<br>Age last follow-up 17 and 20 yrs<br>1 N314D/N314D, male<br>Age last follow-up 20 yrs<br>1 D314D/normal, female<br>Age at last follow-up 31 yrs	7/14 older than 12 yrs:<br>1/7: anomalies of motor function<br>2/7: constructive apraxia<br>1/7: intellectual disability<br>4/7: apraxia of speech<br>1/7: anxiety<br>7/14 younger than 12 yrs:<br>1/7: apraxia of speech (9 yrs)<br>4/4 other genotypes: no neurological abnormalities	Not specified how any of the long-term outcomes were measured or defined
Waggoner et al. 1990	16	Specialist questionnaire on long-term prognosis	371 questionnaires on long-term outcome, 350 cases included<br>178 male, 172 female<br>Age 2 weeks-37 yrs, mean 9.5 yrs	In response to specific questions about co-ordination and gait, 37 out of 206 cases (18%) who were more than 3.5 years old were described as having problems with co-ordination (26 cases), gait (14), balance (7), fine motor tremors (9), and severe ataxia (2 teenage cases).No observable differences in treatment or biochemical factors between 56 cases who had normal intellectual, speech and motor function compared to 25 cases who were developmentally delayed and had speech and motor problems	
Waisbren et al. 2012	17	Cross-sectional study to assess the adult phenotype	33 CG patients, 17 male, 16 female<br>Age 18-59 yrs, mean 32.6±11.7 yrs	15/33 (46%): tremor (8/14 intention, 5/14 postural, 2/14 both)<br>5/33 (15%): ataxia<br>2/33 (6%): dystonia	

Recommendation #20

First author,	#	*Study design*	*Study population (number & age)*	*Results*	*Remarks*
Hughes et al. 2009	1	Retrospective case series to assess outcomes in siblings with CG, including neurologic and neuroimaging findings	12 CG patients, 19 male, 11 female<br>Age 3-25 yrs (average age at MRI: 12.1 yrs)	15 scans in 12 patients<br>In 10/12: abnormal deep cerebral white matter, most often involving delayed or absent myelination<br>2/12: some evidence of white matter volume loss<br>7/12 : prominent cerebellar sulci consistent with some cerebellar atrophy<br>2/12 : mildly dilated lateral ventricles<br>All MRS studies were normal for age<br>2 subjects had essentially the same very abnormal white matter on repeat studies but exhibited progression of the cerebellar atrophy between studies	
Kaufman et al. 1995	2	Cross-sectional study on cognitive functioning, neurologic status and brain imaging	45 CG patients, 23 male, 22 female<br>Age 4-39 yrs<br>MRI scan in 40 patients	37/40: abnormal white matter signal<br>7/40: mild cerebral atrophy<br>16/40: large ventricular size<br>8/40: focal white matter lesions of 1-15 mm scattered throughout the cerebral white matter that tended to cluster in the vicinity of the horns of the lateral ventricles<br>MRI finding do not correlate with age at diagnosis, severity of illness at presentation or cognitive outcome	

First author,	#	Study design	Study population (number & age)	Results	Remarks
**Nelson et al. 1992**	3	Retrospective case series to assess magnetic resonance imaging appearance of the brain	63 CG patients and 4 variant patients, 31 male, 36 female<br>Age 1 mnth-42 yrs, median 10 yrs	67/67: appropriate myelination for age on T1-weighted images<br>8/8 CG patients ≤1 yrs of age: normal white matter signal<br>52/55 CG patients >1 yrs of age: peripheral and cerebral white matter signal did not become as hypointense on intermediate and T2-weighted images as in normal controls. The internal capsule and corpus callosum remained of normal low signal intensity on T2-weighted images<br>9/12 <2 yrs had follow up MRI 1-2 yrs after initial MRI: 9/9 developed abnormal peripheral myelin pattern<br>15 patients 2-25 yrs had follow-up **MRI after 1-4 yrs: abnormal peripheral myelin pattern evident at first MRI in all and unchanged at follow-up**<br>22/63: mild lateral ventricular enlargement **(4/22 had follow up after 1-2 yrs: size unchanged). 0 of other 20 patients developed enlarged ventricles on follow-up.**<br>8/63: enlargement of 4th ventricle and cerebellar sulci, suggesting cerebellar atrophy<br>11/63: 2 or more 2-15 mm diameter lesions scattered through cerebral white matter<br>3/4 variant patients: no abnormalities on MRI<br>4/4 variant patients: no focal white matter lesions, normal sizes cerebral sulci, cerebellar fissures and ventricular systems	

Recommendation #21 (numbers 1, 2, 4, 6, 8, 9, 10
Recommendation #22 (numbers 1, 3, 4, 5, 7, 9, 10)
Recommendation #23 (number 5, 7)

First author, year	*#*	*Study design*	*Study population (number & age)*	*Results*	*Remarks*
Antshel et al. 2004	1	Retrospective case series on the neuropsychological and behavioural profile of children and adolescents	25 CG patients, 15 male, 10 female<br>Age 8-14 yrs, mean 10 yrs 9 mnths±2 yrs 2 mnths<br><br>20 controls, 11 male, 9 female<br>Age 8-13 yrs, mean 10 yrs 8 mnths±1 yr 1 mnth)	**Child behaviour checklist**<br>CG patients: no externalizing symptoms but more internalizing features. Internalizing scale: CG patients rated as higher than control participants<br>**Children's depression inventory**<br>CG patients don't report elevated levels of dysphoric mood, compared with control participants.	

First author, year	#	Study design	Study population (number & age)	Results	Remarks
**Bosch et al. 2004**	2	Cross-sectional study of health-related quality of life, educational levels and specific galactosemia-related concerns, with questionnaires	63 CG patients, 24 male, 39 female<br>Age 1-41 yrs	75 CG patients received questionnaire, 63 from 58 families returned it (84%)<br>**TAPQOL questionnaire**<br>Higher frequency of abdominal pain and colic, and more problems with understanding what others say, problems with speaking clearly, and more difficulties with active and passive use of language<br>**TACQOL questionnaire**<br>Significantly lower HRQoL on the domains of motor function and cognitive function<br>**TAAQOL questionnaire**<br>Significantly lower scores on the domains of cognitive function and social function<br>**Quality of Life Survey**<br>Unvalidated questionnaire to obtain an impression of the effects of the disorder on the daily lives of the patients and their families<br>**Patients ≥8 yrs**CG is seen as a burden by 39% of patients. Some (34%) feel different because of having CG, and 22% believe that their disease is not well understood by others. Few patients worry frequently about their future, most patients (91%) believe that one can live a good<br>life with CG. Most patients (80%) report being treated by their parents the same way as their healthy siblings. Worries about possible infertility are reported by 28% of the girls ≥8 yrs	

First author, year	#	Study design	Study population (number & age)	Results	Remarks
**Bosch et al. 2009**	3	Cross-sectional study on 'course of life' using a questionnaire	15 CG patients, 3 male, 12 female<br>Age mean 24.2± 3.9 yrs, range 18-35 yrs<br><br>32 PKU patients, 10 male, 22 female<br>Age mean 24.6± 3.6 yrs, range 18-30.4 yrs<br><br>Also comparison to reference group of peers	Course of life questionnaire<br>CG patients achieved fewer developmental milestones (or at older age) in the psychosexual and social domain than PKU patients and reference group of peers from the general population<br>**Social development**<br>CG patients differed (lower scores) on 6/12 items from the PKU patients and/or their peers from the general population<br>**Psychosexual development**<br>Significant differences between CG patients and the other groups on 2 items of psychosexual development (older at time of first boy/girlfriend, older at time of first sexual intimacy)<br>**Sociodemographic outcomes**<br>Percentage of patients living together or being married significantly lower in the reference group. Patients significantly less frequently employed than the reference group	
**Gubbels et al. 2011**	4	Cross-sectional study to assess psychosocial development in males	18 male CG patients<br>Age mean 24.2±4.2 yrs, range 18.1-35 yrs	Course of life Questionnaire<br>**Social development:**<br>CG males scored significantly lower on 8/12 milestones than the reference group.<br>**Psychosexual development and marital status**<br>Patients scored significantly lower on all 4 milestones of psychosexual development than the reference group. 1/18C CG men was married (lower than reference group but not significant)<br>**Autonomy development**<br>No significant differences on the milestones of Autonomy development (regular chores/tasks in the family, paid jobs, vacation without adults, leaving the parent's home)	

First author, year	#	Study design	Study population (number & age)	Results	Remarks
**Hoffman et al. 2012**	5	Cross-sectional study on living situation, occupation and health-related quality of life	41 CG patients, male 18, female 23<br>Age mean 26.2±7.2 yrs (18.3-49.3)	The majority of patients were singles, with males more than twice as often as females. 2 males compared to 13 females were married or living in a stable partnership, more males compared to females still living with their parents or under supervised living conditions (66.7 % vs. 52.1%)<br>1 male but 9 females were living with their partner or with friends. Own children were reported by 2 (female) patients only. Desire for children reported by nearly 50% of the patients, with a higher proportion in males than in females (66.7% vs. 44.4%)<br>**Employment**<br>24.2%: not employed at all, 54.5% were either working part-time or full-time, no differences between males and females<br>**HRQoL**<br>Highest scores reported for the dimension "negative mood" , followed by "psychological functioning" and the dimension of "social well-being"<br>Lowest scores reported for "positive mood"<br>Positive mood in patients with galactosemia significantly lower compared to both, the general German population and PKU patients<br>Social well-being and social functioning also lower in patients with CG compared to PKU patients<br>"Negative mood" showed good correlation with burden of the diet<br>**Disease specific information**<br>> 75% of the patients rated their coping with CG as 'very good' or 'good', the remainder had difficulties in getting along with the disease<br>58%: not a problem at all keeping the diet, but at least partly straining for nearly every third patient<br>56.1% did not feel burdened to inform other people about the disease, 61.0% of patients reported that it does not affect them to compare their life with others'<br>>60% reported that CG does not at all impair their company with other people. 30% reported that this was in part true, 9.8% reported that the disease impairs their company with other people<br>"diet/nutrition" is the primary aspect of life influenced by the disease, followed by "school/work" and "friends/leisure". Affection of family life was reported by	

First author, year	#	Study design	Study population (number & age)	Results	Remarks
Komrower et al. 1970	6	Retrospective case series on long-term outcome and psychological and emotional state	60 CG patients, 22 male, 38 female<br><br>Bristol Social Adjustment Guide sent to schools of:<br>30 CG children, 18 male, 12 female<br>Age >5 yrs	Scales on which children score most heavily: unforthcomingness, depression and hostility to adults<br>Parent interviews provided a general impression regarding a sensitive temperament and difficulties making friends	
Lambert et al. 2004	7	Cross-sectional study to assess the impact of CG on quality of life	13 CG patients, 5 male, 8 female<br>Age >6 yrs<br>12 individual parents of CG patients	CG patients do not differ from their peers in their physical activities, mobilization, overall health and their self esteem<br>Despite feeling 'not as good as most people', all patients had been happy at some point in the 4 weeks preceding the interview.<br>CG patients have difficulties in their relationships with others<br>Many patients experience difficulty sleeping at night and all patients reported some level of difficulty staying awake during the day<br>4/14: got 'really mad very often' and 4/13: got 'really mad' sometimes	
Lee et al. 1972	8	Retrospective case series on psychological aspects	60 CG patients, 22 male, 38 female<br>Age mean 8 yrs 5 mnths, range 2 yrs 2 mnths-17 yrs 7 mnths<br><br>BSAG completed for 30 CG patients, 12 male, 18 female<br>Age mean 10.0 yrs	BSAG:<br>15/30: symptoms of emotional disturbance<br>23%: sufficiently high scores to be regarded as maladjusted and 27% unsettled.<br>Scales on which children score most heavily: unforthcomingness, depression and hostility to adults	

First author, year	#	Study design	Study population (number & age)	Results	Remarks
Rubio-Agusti et al. 2013	9	Cross-sectional study to determine the frequency and phenotype of motor dysfunction with retrospective data on non-motor features including neuropsychiatric symptoms	47 CG patients, 18 male, 29 female<br>Age 20-38 yrs, median 26 yrs	11/47 (23%): neuropsychiatric symptoms including: 5/47 anxiety, 4/47 depression (1 suicide attempt), 3/47 obsessive-compulsive disorder, 2/47 autistic spectrum disorder, 1/47 social phobia, 1/47 paranoid delusions	
Waisbren et al. 2012	10	Cross-sectional study on the adult phenotype, with behavior assessment and executive functions	33 CG patients, 17 male, 16 female<br>Age 18 to 59 years, mean 32.6±11.7 yrs, median 31 yrs	7/33 (21%): scores < 85 on the ABAS<br>15/33 (46%): live independently, including 9 who were either married or living with partners.<br>Average schooling was 1–2 years of college<br>7/33 (21%)): unemployed (and not in school).<br>4/33(12%): depression on BDI (2/4 treated with anti-depressant medication. 6/33 reported having received antidepressant medication at some point<br>3/33: suffered from depression in the past<br>13/33 (39%): observed or reported depression, compared to 16.2% lifetime prevalence in the general adult population in the US<br>17/33 (52%): anxiety on BAI (3/17 taking medication for anxiety), compared to 16.4% yearly prevalence of anxiety in the general adult population in the US<br>5/33: received medication for anxiety in the past<br>22/33 (67%): history of anxiety	

Recommendation #24 (numbers 1, 2, 3, 4, 5, 6, 7, 8, 9, 10, 11)
Recommendation #25: No literature available to support this recommendation
Recommendation #26 (numbers 12, 13, 14, 15)
Recommendation #27 (number 17)
Recommendation #28: No literature available to support this recommendation

First author, year	#	*Study design*	*Study population (number & age)*	*Results*	*Remarks*
Coss et al. 2013	1	Retrospective case series on Irish birth incidence of CG and long-term clinical outcomes	130 CG patients<br>34 females age ≥13 yrs endocrine function	HH (based on LH and FSH): 31/34 females (91.2%)<br>11/11 Travellers (100%), 20/23 non-Travellers (87%)	
Guerrero et al. 2000	2	Retrospective case series and cross-sectional study to identify risk factors of POF	53 female CG patients<br>Age >1 yrs	POF (raised LH and FSH) present in 77.4%.<br>Elevated FSH levels in 85% of girls <10 yrs	
Kaufman et al. 1981	3	Retrospective case series on gonadal function	Evaluation of HH in 18 female CG patients<br>Age 9-29 yrs	HH in 12/18 (high FSH/LH, low estradiol). 5/12 primary amenorrhea, 6/12 secondary amenorrhea, 1/12 oligomenorrhea	
Kaufman et al. 1986	4	Retrospective case series on gonadal function	26 female CG patients, age 1-30 yrs<br>8 new female patients, age 1-12 yrs	12 initial patients with HH: continued evidence of ovarian failure 4 years later(either primary or secondary amenorrhea).<br>5/6 females with normal gonadal function in the original study: evidence of HH on repeat evaluation 4 years later: 2/6 secondary amenorrhoea, 2/6 primary Amenorrhoea, 2/6 oligomenorrhea.<br>0/8 females age 1-12 yrs had elevated FSH/LH. 7/8 abnormal response to LHR.	This study follows the original 18 female patients (Kaufman et al. 1981) for 4 yrs and expands the number of female patients studied to 26

First author, year	#	Study design	Study population (number & age)	Results	Remarks
Kaufman et al. 1994	5	Retrospective case series to determine the correlation of cognitive, neurologic and ovarian outcome with the Q188R mutation	67 CG patients, 35 females, tests in females >13 yrs, number unknown	22/? patients elevated FSH and amenorrhea: 8/22 primary amenorrhea and failure to complete pubertal development, 14/22 had secondary amenorrhea<br>No statistical relationship between the frequency of Q188R homozygosity, heterozygosity, or negative status and the development of primary or secondary amenorrhea	
O'Herlihy et al. 1985	6	Cross-sectional study to evaluate ovarian function	6 female CG patients<br>Age 8-17 yrs	Elevated FSH in 5/6 and LH in 3/6<br>1 patient was given exogenous HPG intramuscularly for 3 days with no response	
Rubio-Agusti et al. 2013	7	Cross-sectional study on frequency and phenotype of motor dysfunction, and correlation with POF	47 CG patients, age 20-38 yrs, median 26 yrs<br>29 female CG patients	26/29 (90%): POF<br>3/29, all without motor dysfunction, had normal gonadotropin levels and regular periods. 1 of them had 2 natural pregnancies	Premature ovarian failure was defined by an FSH level>40 IU/L on 2 consecutive determinations
Rubio-Gozalbo et al. 2006	8	Cross-sectional study to evaluate the endocrine system	25 female CG patients<br>Age 5-19 yrs	15/18 patients (not on estrogen replacement) elevated FSH, estradiol not elevated	
Schweitzer et al. 1993	9	Cross-sectional study/Retrospective case series on long-term outcome of early diagnosed patients	134 CG patients<br>Evaluation of HH in 11 female patients age >12 yrs	5/11 HH, primary amenorrhoea and delayed puberty	
Waggoner et al. 1990	10	Specialist questionnaire on long-term prognosis	371 questionnaires returned by 41 specialists<br>Evaluation of ovarian function<br>Evaluation of menstrual history	FSH elevated in 80% of 75 females <18 yrs<br>FSH elevated on repeated tests in 67% of 40 females<br>Mean age at menarche: 14 yrs, range 10-18 yrs<br>8/34 females >17 years have primary amenorrhea<br>5/17 >22 yrs have normal menstruation<br>In 47 patients ≥15 yrs : 81% signs of abnormal ovarian function (absent or abnormal menstrual cycles or elevated FSH)	

First author, year	#	Study design	Study population (number & age)	Results	Remarks
Waisbren et al. 2012	11	Cross-sectional study to assess the adult phenotype	Endocrine evaluation in 16 female CG patients<br>Age 18 to 55 yrs	All females previously diagnosed with POI<br>Estradiol: range 10 pg/mL- 129 pg/mL (mean=33±36; reference range=21–649 pg/mL)<br>FSH: range 0.14-109.44 (mean=32.77±32.99; reference range=1.38-16.69 mlU per mL)<br>11/16 spontaneous menarche. Average age of menarche was 15.1±1.8 yrs for women, including those receiving hormonal treatments to induce menstruation	POI not otherwise described
			AMH		
Gubbels et al. 2013a	12	Cross-sectional study on POI and the role of FSH dysfunction	24 female CG patients, age 6-43 yrs, AMH levels measured<br>3 female patients stimulated with exogenous FSH, age unknown<br>9 female patients stimulated with FSH and LH 3 days, 3 patients stimulated 21 days, age unknown	24/24 undetectable AMH levels (<0.1 µg/l)<br>0/3 responded to exogenous FSH<br>3/9 had an increase of 17-beta-estradiol after stimulation with 3 days FSH and LH, and 2/3 showed a response after 21 days stimulation	
Sanders et al. 2009	13	Cross-sectional study to evaluate biomarkers of ovarian function	35 CG patients, age <1-46 yrs<br>43 control girls and women, age <1-51 yrs	AMH levels in CG patients lower than in healthy controls across all age groups<br>FSH levels in CG patients higher than in controls across all age groups, however <8 years almost half of patients have normal levels of FSH, but most have abnormal levels of AMH<br>No evidence for diminished bioactivity of FSH in CG	

First author, year	#	Study design	Study population (number & age)	Results	Remarks
Spencer et al. 2013	14	Cross-sectional study to assess modifiers of ovarian function	AMH measurements in 300 plasma samples representing 158 female CG patients, and 96 plasma samples representing 96 controls, both groups, age <1 mnth-30 yrs	AMH in CG patients is significantly lower when compared to controls in all age groups<br>At age 18 mnths for the vast majority of CG girls, AMH remained very low to undetectable, for a small number AMH rose into the detectable or normal range.	
Waisbren et al. 2012	15	Cross-sectional study to assess the adult phenotype	AMH measurements in 16 female CG patients<br>Age 18 -55 yrs	AMH levels of all females: mean=0.025±0.022 ng/ml (range=0.01-0.07 ng/ml).	
			Adrogens		
Kaufman et al. 1987	16	Cross-sectional study to assess androgen production	5 female CG patients with HH, age 15-27 yrs<br>3 controls (healthy postmenopausal) females, age 49-63	Basal serum levels of androstenedione, testosterone and estrogen in CG patients are lower than normal, comparable to postmenopausal controls. Normal DHEAS levels. Suppression of androstenedione and testosterone after dexamethasone	HH not otherwise described
			Duarte Galactosemia		
Badik et al. 2011	17	Cross-sectional study to evaluate AMH and FSH levels in DG patients	57 female patients with DG (89 female patients with CG and 64 female controls)<br>Age <1 mnth-10.5 yrs	AMH levels DG patients in neonatal period, ≥3 mnths to 18 mnths and ≥18 mnths to 10.5 yrs not different from control girls	

Recommendation #29
Recommendation #30

First author, year	#	*Study design*	*Study population (number & age)*	*Results*	*Remarks*
Gubbels et al. 2008	1	Cross-sectional study with semi-standardized interview concerning fertility and pregnancies	22 female CG patients<br>Age >18 yrs	9 patients tried to conceive, 5 were unsuccessful and 4 gave birth to a child (44%). The time to pregnancy ranged from 1 mnth to over 2 yrs	
Waggoner et al. 1990	2	Specialist questionnaire on long-term prognosis	371 questionnaires returned by 41 specialists<br><br>Evaluation of pregnancies in 37 CG patients, age >17 yrs	14 pregnancies in 9/37 women<br>Information about menstrual history was provided for 6/9 mothers. 2/6: never had normal menstrual periods<br>3/6: developed menstrual irregularities after their children were born<br>1/6: normal menstruation both before and after giving birth (in text referred to as black woman)	
Waisbren et al. 2012	3	Cross-sectional study to assess the adult phenotype	Long-term outcome in 16 female CG patients.<br>Age 18-55 yrs	1/4 patients who tried to conceive became pregnant after 60 mnths of trying and gave birth to a healthy child	

Recommendation #31 : No literature available to support this recommendation
Recommendation #32 : No literature available to support this recommendation

Recommendation #33

First author, year	#	*Study design*	*Study population (number & age)*	*Results*	*Remarks*
Gubbels et al. 2011b	1	Cross-sectional study to assess the male reproductive system	26 male CG patients, age 19-59 yrs<br>46 male control patients, age 25-45 yrs	Cryptorchidism 11.6% in CG compared to 1.0% at age 3 mnths in control. Lower semen volume in CG compared to controls. Testosterone, inhibin B, free androgen index and sperm concentrations lower and SHBG higher than in control subjects. Most values of testosterone and inhibin B were in reference range. Gonadotropin levels normal.	
Kaufman et al. 1981	2	Retrospective case series on gonadal function	8 male CG patients<br>Age 13 yrs-28 yrs	8/8 normal pubertal development, serum FSH/LH and testosterone levels	
Kaufman et al. 1986	3	Retrospective case series on gonadal function	Evaluation of gonadal function in 12 male CG patients<br>Age 5-32 yrs	12/12 normal pubertal development and serum levels of FSH, LH, and testosterone<br>2/12 (32 and 21 yrs) had semen analyses; normal volume and sperm morphology, motility and number<br>3/12 (32, 25 and 18 yrs) administration of LRH: normal pubertal rise in LH, FSH and testosterone levels	This study follows the original 8 male patients (Kaufman et al. 1981) for 4 years and expands the number of patients studied to 12
Rubio-Gozalbo et al. 2006	4	Cross-sectional study to assess the endocrine system	12 male CG patients, age 6-19 yrs	Cryptorchidism in 3/12 CG patients compared to <1% in the normal population at 1 yr of age<br>Levels of IGF-1 and IGFBP-3 normal. No clear abnormalities in large subset of screened hormones (total cholesterol, triglycerides, HDL, LDL, prolactin, TSH, TBG, FT4, basal testosterone, free testosterone, andostenedione and DHAES). 12/12 normal FSH, LH and estradiol.	
Schweitzer et al. 1993	5	Cross-sectional study/Retrospective case series on long-term outcome of early diagnosed patients	134 CG patients<br>Evaluation of puberty in 18 male patients, age >12 yrs	1/18: delayed puberty, assessed according to Tanner (confirmed by hormone analysis)	

First author, year	#	Study design	Study population (number & age)	Results	Remarks
Waisbren et al. 2012	6	Cross-sectional study to assess the adult phenotype	17 male CG patients<br>Age 20-59 yrs	Average age of self-reported puberty 13.7±1.8 yrs (in general population at time of study about 14 yrs for boys)<br>FSH: range 1.31-8.49 mlU per mL, mean 4.33±2.6 (reference range= 0.95-11.95 mlU per mL)<br>Cryptorchidism: 1/17 (at birth)<br>Testicular size: within normal range (mean= 28 ±4.06 cc, range=25-35 cc)<br>8 men provided a semen sample: 1/8 low sperm count and 2/8 low percent normal morphology	

Recommendation #34

First author, year	#	*Study design*	*Study population (number & age)*	*Results*	*Remarks*
Batey et al. 2013	1	Cross-sectional study on skeletal outcome	33 CG patients, 17 male, 16 female<br>Age 18-59 yrs, mean 32.0±11.8 yrs	**BMD assessed with DXA**<br>BMD lumbar spine/total hip:<br>Females: 0.903/0.799 cm^2<br>Males: 0.991/0.896 cm^2<br>**BMD Z-scores lumbar spine/total hip:**<br>Females: −1.19±1.14/ −1.25±0.79<br>Males: −0.80±1.28/ −0.81±0.70<br>Absolute BMD lower on average in women than in men for both lumbar spine and hip<br>BMD Z-score <2.0 for females and males: 33 vs. 18 % at the lumbar spine, 27 vs. 6 % at the hip<br>Greater self-report of fractures in females compared to males: 63 % of the women and 31 % of men had sustained at least one lifetime fracture<br>Body weight and BMI moderately correlates with BMD-Z at the hip in women, and BMI correlated moderately with BMD Z-score at the spine in men	This study includes same cohort of subjects as Waisbren et al. 2012
Coss et al. 2013	2	Retrospective case series of cases detected by newborn screening since 1972 and analysis of outcomes of all cases followed clinically	130 CG patients, male 74, female 50, 6 unknown, age 0.5-45 yrs<br><br>DXA in 72 patients, sex and age unknown	Osteopenia or osteoporosis in:<br><br>Traveller population: 5/34 (14.7%)<br><br>Non-Traveller population: 19/48 (39.6%) (significant difference between Travellers and non-Travellers)	Presence of osteopenia documented by Z-scores generated from DXA scans used to determine BMD in patients ≥10 yrs, osteopenia and osteoporosis not defined

First author, year	#	Study design	Study population (number & age)	Results	Remarks
Doulgeraki et al. 2014	3	Cross-sectional study on whole body composition, including BMD	14 CG patients, 8 male, 6 female<br>Age 6.17-16.58 yrs	BMD assessed with DXA<br>BMD lumbar spine Z-score: mean -0.65 (-2.5 – 1.4)<br>BMD total body Z-score: mean -0.3 (-1.7 – 0.6)	
Karadag et al. 2013	4	Retrospective case series of features and outcome of CG patients diagnosed in newborn period	22 CG patients<br>Median follow-up time 3 yrs, range 1-6 yrs	DXA and qualitative ultrasound once per year<br><br>Decreased bone mineral density: 0/22 (0%)	Decreased bone mineral density not defined
Kaufman et al. 1993	5	Cross-sectional study of BMD in relationship to dietary history and, in female patients, with gonadal function	40 CG patients, 22 male, 18 female<br>Age 3.4-44.2 yrs<br><br>40 healthy controls, age and sex matched	Quantitative CT measurements of lumbar bone density (mg/cm³) patients/controls:<br>**Significance P<0.001**<br>All females: 123.7±25.2/161.2±16.7<br>All males: 133.2±21.3/156.6 ±21.3<br>All patients: 130±25.0/158.7±12.2<br>Adult women: 120.6±26.9/163.9±18.0<br>Adult women without estrogens: 92.4 ±14.3/160.2±20.2<br>**Significance 0.002:**<br>Adult women with estrogens: 136.3±17.3/166.0±17.5<br>**Significance 0.008:**<br>Prepubertal children: 133.5±20.6/150.4±10.4<br>Adult men: 133.2±20.8/163.7±26.7<br>**Z-scores patients/controls (mean ± range):**<br>Children (15): -0.9 (-3.0-0.8) / 0.1 (-1.2-0.6)<br>Females (14): -1.9 (-4.2-0.2) / 0 (-1.6-0.5)<br>Males (11): -1.4 (-3.0-0.2) / 0 (-1.6-1.7)<br>No correlation of BMD with calcium intake in prepubertal patients and women not receiving replacement sex steroids vs. positive correlation in postpubertal men and women receiving replacement sex steroids	

First author, year	#	Study design	Study population (number & age)	Results	Remarks
Panis et al. 2004	6	Cross-sectional study of bone metabolism and BMD	40 CG patients,13 male, 27 female<br>Age 3.0-17.3 yrs, mean 8.9±4.1 yrs	BMD assessed with DXA<br>Areal BMD Z-score of femoral neck: -0.3±0.9 (-1.6-1.4)<br>Volumetric BMD of femoral neck: 0.28±0.03 g/cm³ (0.22-0.35)<br>Areal BMD Z-score of lumbar spine: -0.6±0.8 (-2.2 to 1.4)	
Panis et al. 2006	7	Randomized-controlled trial on effect of calcium, vitamin K1 and D3 supplementation on bone mineral content	40 CG patients with a diminished bone mineral content (BMC of femoral neck or lumbar spine <0.5 SD), 13 male, 27 female<br>19 in the treatment group and 21 in the placebo group, age 3-17 yrs	Significant increase in BMC of lumbar spine in treatment group compared to placebo group, but only in prepubertal children	2-year, double-blind, placebo-controlled clinical trial randomized in 2 groups<br>Group 1: receiving daily two tablets, each containing 375 mg calcium, 0.5 mg vitamin K1 and 5.0 mg vitamin D3<br>Group 2: receiving placebo
Rubio-Gozalbo et al. 2002	8	Cross-sectional study on BMD and blood parameters involved in bone formation	11 CG patients, 5 male, 6 female<br>Age 2.5-18 yrs	BMD assessed with DXA<br>Areal BMD (total body) Z score (6 patients, >5 yrs): mean -0.99, range -0.5 to -1.4.<br>Volumetric BMD (femoral neck) Z score 11 patients: mean -1.76, range -0.7 to -3.3<br>Conclusion<br>Areal BMD of the total body and volumetric BMD of the femoral neck Z-scores are significantly decreased	
Waisbren et al. 2012	9	Cross-sectional study on long-term outcome	33 CG patients, 17 male, 16 female<br>Age 18-59 yrs, mean 32.6±11.7 yrs	DXA total hip, femoral neck, lumbar spine<br>Low BMD, defined as z-scores ≤-2: 8/33 (24%)<br>Results do not differentiate between measurement sites	This study includes same cohort of subjects as Batey et al. 2013

Recommendation #35: No literature available to support this recommendation

Recommendation #36 (numbers 1, 4, 5, 6)
Recommendation #37 (numbers 2, 3, 5, 6, 7)

First author, year	#	*Study design*	*Study population (number & age)*	*Results*	*Remarks*
Batey et al. 2013	1	Cross-sectional study on skeletal outcome	33 CG patients, 17 male, 16 female<br>Age 18-59 yrs, mean 32.0±11.8 yrs	Circulating Ca level is a predictor of hip and spine BMD in both sexes, and osteocalcin and gonadotropin levels are inversely correlated with spinal BMD in the women<br>Ca intake correlated with lumbar spine BMD in women only<br>C-telopeptides and OC: moderate and significant inverse correlation with BMD Z-scores at both hip and lumbar spine in women<br>No correlation noted between bone density and estradiol concentrations in women<br>Higher gonadotropin levels associated with lower spinal BMD in women	
Gajewska et al. 2006	2	Cross-sectional study on serum bone turnover markers	35 CG patients, 17 male, 18 female<br>Age 1-10 yrs, mean 5.0 yrs<br><br>55 healthy controls, 30 male, 25 female<br>Age 1-10 yrs, mean 7.0 yrs	Similar to controls:<br>BAP: mean 106.4 U/l (range 39.8-164.1)<br>OC: 119.1 µg/L (range 35.0-180.6)<br>20% lower than controls:<br>CTX1: 12075 pmol/l (range 5082-24787)<br>Normal values of:<br>Calcium: mean 2.27 mM (range 2.27-2.77)<br>P: mean 1.65 mM (range 1.32-1.92)<br>25-OH-D: mean 31.6 µg/L (range 17.3-59.3)<br>AP: mean 232 U/L (range 139-319)	

First author, year	#	*Study design*	*Study population (number & age)*	*Results*	*Remarks*
Gajewska et al. 2008	3	Cross-sectional study on serum bone turnover markers	62 CG patients, 32 male, 30 female<br>Age range 2-20 years,	**Patients aged 2-9 years**<br>Values of BALP and OC similar to controls, values of CTX-1 20% lower than controls<br>**Patients aged 10-20 years:**<br>Values of BALP, OC and CTX1 higher by about 30%, 35% and 15% respectively than in controls<br><br>Values of BAP, OC and CTXI about 30%, 20% and 20% lower respectively in healthy children compared to adolescents. These values were comparable between CG children and adolescents<br>CG patients: Ca 2.46 mM (2.20-2.77), P 1.56 mM (1.01-1.92), 25OH-D vitamin 27.3 µg/L (17.2-59.3) in normal ranges	
Kaufman et al. 1993	4	Cross-sectional study of BMD in relationship to dietary history and, in female patients, with gonadal function	40 CG patients, 22 male, 18 female<br>Age 3.4-44.2 yrs<br><br>40 healthy controls, age and sex matched	No correlation of bone density with dietary calcium intake in prepubertal patients.<br>Significant correlation between bone density and dietary calcium intake for women receiving ERT, but not for women not receiving ERT.<br>Significant correlation of bone density and calcium intake in postpubertal male patients. No difference in protein intake between males and females and no correlation with bone density.	
Panis et al. 2004	5	Cross-sectional study of bone metabolism and BMD	40 CG patients, male 13, female 27<br>Age 3.0-17.3 yrs, mean 8.9±4.1 yrs	Normal concentrations of Ca, P , Mg, Z, 1.25OHD, PTH, 17-beta-oestradiol<br>cOC, NTX, CTX, and IGF-1 Z-scores are significantly decreased in CG patients	

First author, year	#	*Study design*	*Study population (number & age)*	*Results*	*Remarks*
Panis et al. 2006	6	Randomized-controlled trial on effect of calcium, vitamin K1 and D3 supplementation on bone mineral content	40 CG patients with a diminished bone mineral content (BMC of femoral neck or lumbar spine <0.5 SD), 13 male, 27 female<br>19 in the treatment group and 21 in the placebo group, age 3-17 years	No patients showed increased urinary Ca excretion<br>PTH at baseline normal in all patients<br>Significant decrease in the treatment group over the 2-year study period in ucOC concentration in the total group, prepubertal and pubertal groups	2-year, double-blind, placebo-controlled clinical trial randomized in 2 groups<br>Group 1: receiving daily two tablets, each containing 375 mg calcium, 0.5 mg vitamin K1 and 5.0 mg vitamin D3<br>Group 2: receiving placebo
Rubio-Gozalbo et al. 2002	7	Cross-sectional study on BMD and blood parameters involved in bone formation	11 CG patients, 5 male, 6 female<br>Age 2.5-18 yrs	Ca, P, PTH, vitamin D metabolites all within normal laboratory reference values<br>No significant differences between CG patients and healthy controls for OC (ucOC and cOC) and BAP.<br>NTX levels in blood were significantly lower ($p < 0.001$) than in healthy controls	

Recommendation #38 (numbers 1, 2, 4, 5, 6, 8, 9, 10, 12)
Recommendation #39 (numbers 8, 9, 11)
Recommendation #40 (numbers 3, 4)

First author, year	#	*Study design*	*Study population (number & age)*	*Results*	*Remarks*
Badawi et al. 1996	1	Retrospective case series on clinical outcome in patients detected by NBS 1972-1992	32 patients CG followed up from diagnosis, sex unknown<br><br>Age 2 weeks-20 yrs	Mean age start diet: 7.6 days<br>2/32 cataract at presentation (day 8 and 19)<br>3/32: persistent opacities, not visually significant.<br>8 other patients developed lens opacities, which regressed totally on strict adherence to diet	
Beigi et al. 1993	2	Case series on ophthalmic findings	33 CG patients, 15 male, 18 female<br>Ophthalmological examination from diagnosis. Mean observation time 8.5 yrs, 16 patients observed for 10 yrs<br><br>All, except for 3, diagnosed <4 weeks of age. 1 at 6 weeks, 1 at 3 months, 1 at 8 yrs<br><br>Divided into 3 groups: very good, good, and fair biochemical control, on the basis of their mean erythrocyte Gal-1-P	12/33 developed lens opacities in the form of posterior subcapsular, cortical, and nuclear minute opacities. 1/33 had familial autosomal dominant nuclear cataract (stationary)<br><br>**Very good control (23 patients):** 13/23 no cataract, 1/23 congenital cataract, 2/23 developed lens opacities during study (still present at end of study), 6/23 lens opacities at beginning of study and cleared later, 1/23 developed peripheral axial lens opacities at age 2-5 years during a 3 mnth period off the diet. Regressed completely over 6 mnths after reintroduction diet. Lens opacities developed 2 yrs later after another period of poor compliance and regressed over 3 yrs with tighter biochemical control<br><br>**Good control (8 patients):** 2/8 cataracts, 1/8 lens opacities at start but cleared, 1/8 developed peripheral axial lens opacities at 3-7 yrs following a 3 mnth period off all dietary restrictions. Good control re-established and the opacities regressed totally over a 9 mnths<br><br>**Fair control (2 patients):** 1/2 lens opacities initially that cleared later	16 patients also included previously in Burke et al. 1989

First author, year	#	Study design	Study population (number & age)	Results	Remarks
Burke et al. 1988	3	Retrospective case series plus cross sectional ophthalmic examination	18 CG patients, 7 male, 11 female<br>Follow-up time: 1.9-8.3 yrs<br><br>24 parents, mean age 34.9 yrs (range 23-54 yrs)<br>Diagnosis all <42 days, except 1 case diagnosed at 12 yrs	**CG patients**<br>11/18: no lens opacities on examination (cross-sectional), 0/11 cataract at diagnosis<br>7/18: lens opacities, 4/7 lens opacities at diagnosis, 2/7 developed lens opacities during 3-month off diet and regressed in 1 of the 2 after improved dietary control, 1/7 stationary pulverant cataract identical to that of her mother	Short communication
Burke et al. 1989	4	Retrospective case series on ophthalmic findings	17 CG patients, 7 male, 10 female<br>Age 1.5-15.8 yrs<br>Mean follow-up period: 6.1 yrs, range 1.5-15.8 yrs	Diet started <42 days of age in all patients<br>4/17 patients: lens opacities at examination, not visually significant<br>1/17: peripheral axial lens opacities at 3.7 yrs follow-up following 3 month off dietary treatment. Resolved after 9 mnth period with dietary control<br>4/17: cataract at diagnosis, 1/4 bilateral inner foetal nuclear cataract with sparing of the embryonic nucleus. Abnormalities unchanged since diagnosis. Mother has identical opacities consistent with familial autosomal dominant non-galactosemic cataract	
Coss et al. 2013	5	Retrospective case series of cases detected by newborn screening since 1972 and analysis of outcomes of all cases followed clinically	130 CG patients detected by newborn screening, sex unknown<br>Age 0.5-45 yrs	Eye assessment with slit-lamp evaluation was performed routinely on all affected newborns Subsequently, reviews were performed every 6 months for the first 3 yrs of life and then yearly<br>Cataracts complete cohort: 10/130 (7.7%)<br>Traveller cohort: 1/63 (1.6%)<br>Non-Traveller cohort: 9/67 13.4%<br>No information about follow-up	
Henderson et al. 2002	6	Retrospective case series of the clinical and laboratory features present at the time of diagnosis in	17 CG patients, clinical notes available of 9 CG patients<br><br>Mean age at diagnosis: 5.1 mnths (range: 4 days-6.5 mnths)	Bilateral cataracts: 6/9 patients<br>Of the 17 patients, 6 had S135L/S135L genotype, 3 Q188R/Q188R, 1 Q188R/?	All children diagnosed with CG in the Cape Metropole between 1980 and 2001

First author, year	#	Study design	Study population (number & age)	Results	Remarks
Karadag et al. 2013	7	Retrospective case series of features and outcome of CG patients diagnosed in newborn period	22 CG patients<br>Median follow-up time 3 yrs, range 1-6 yrs	Nuclear cataract in 15/22 patients in newborn period (day 3-23)<br>18/22 early diagnosis (<17 days), 4/22 diagnosis >17 days<br>Nuclear cataract in 13/18 patients with early diagnosis, and in 4/4 patients with late diagnosis<br>Cataracts resolved in 13 patients and 4 patients needed surgery (all diagnosed after age 17 days)<br>None of the patients showed any new cataracts during follow up	Ophthalmological examinations with slit lamp were performed at time of diagnosis, at age 6 mnths, and afterwards once per year
Kossowicz et al. 1975	8	Retrospective case series of cataracts in galactosemia	12 CG patients, 4 male, others unknown, age unknown	4/12 cataract, 3 at birth, 1 after 2 ys. Age at start treatment: 2-10 mnths. In ¼ the peripheral opacities regressed during one year of treatment, the zonular ones being a little less intensive but still impairing visual acuity<br>8/12 no cataract. Age at start treatment all but 1 <2 mnths, 1 patient started at 11 mnths	
Schweitzer et al. 1993	9	Case series and cross-sectional study of clinical outcomes	134 patients, 20 of these patients deceased at time of study<br><br>Complete clinical evaluations in 83 patients, 49 male, 34 female, age 9 months-33 yrs, mean 9.5±7.1 yrs<br>Retrospective evaluations only in 31 patients (patient or parents refused examination), 15 male, 16 female, age 9 months-27 yrs, mean 10.2±8.8 yrs	CG patients (not DG or 'milder' patients):<br>Cataracts had developed in 15 patients with reversible clouding of the lenses after initiation of galactose free diet<br>6 children had mild persistent cataracts<br>A 33- year-old man was blind due to cataract formation<br>1 patient never had ophthalmological examination, but was clinically unaffected	3/83: some residual enzyme activity associated with "mild" galactosemia<br>5/83: DG patients

First author, year	**#**	***Study design***	***Study population (number & age)***	***Results***	***Remarks***
Waggoner et al. 1990	10	Survey among specialist to determine the prevalence of long term complications and their relationships to neonatal and treatment variables	350 CG patients, 51% male, 49% female<br>Age 2 weeks-37 yrs, mean 9.5 yrs	Cataracts in 30% of 314 cases. Nearly half the cataracts described as 'mild', 'transient' or 'neonatal' and resolved with dietary treatment, and 8 treated by surgery<br>Dietary treatment had begun at a mean age of 77 days for those with cataracts compared to 20 days for those without cataracts<br>1/8 who required surgery was an infant who had been treated from birth	
Waisbren et al. 2012	11	Cross-sectional study with chart reviews of ophthalmic findings	33 CG patients, 17 male, 16 female<br>Age 18-59 yrs, mean 32.6±11.7 yrs, median 31 yrs	Cataracts in adulthood noted in medical records or reported during the medical history by 21% of subjects<br>Nystagmus, irregular pupilatory border, and need for bifocals in 1 subject each	
Widger et al. 2010	12	Retrospective case series on incidence and severity of cataracts	100 CG patients<br>14 CG diagnosed with cataracts, 9 male, 5 female<br>13 controls without cataract, age and sex matched	14/100: diagnosed with cataracts<br>Diagnosis of cataract: from the first 4 weeks of life to 19 yrs.<br>Average age at diagnosis of cataract 6.2 yrs: 3 diagnosed in the first 4 weeks, 2 of these regressed spontaneously (one at 6 mnths and the other at 3 yrs of age)<br>Cataracts persisted in 6 patients but do not affect vision<br>With regard to diet: no statistically significant difference in the persistence of cataracts between compliant and poorly compliant patients<br>No significant difference when comparing dietary compliance between the study group and the control group	Compliant: galactose intake <50 mg/day
